# Remote Postdischarge Treatment of Patients With Acute Myocardial Infarction by Allied Health Care Practitioners vs Standard Care: The IMMACULATE Randomized Clinical Trial

**DOI:** 10.1001/jamacardio.2020.6721

**Published:** 2020-12-30

**Authors:** Mark Y. Chan, Karen W. L. Koh, Sock-Cheng Poh, Stephanie Marchesseau, Devinder Singh, Yiying Han, Faclin Ng, Eleanor Lim, Joseph F. Prabath, Chi-Hang Lee, Hui-Wen Sim, Ruth Chen, Leonardo Carvalho, Sock-Hwee Tan, Joshua P. Y. Loh, Jack W. C. Tan, Karishma Kuwelker, R. M. Amanullah, Chee-Tang Chin, James W. L. Yip, Choy-Yee Lee, Juvena Gan, Chew-Yong Lo, Hee-Hwa Ho, Derek J. Hausenloy, Bee-Choo Tai, A. Mark Richards

**Affiliations:** 1 Cardiovascular Research Institute, Yong Loo-Lin School of Medicine, National University of Singapore, Singapore; 2 National University Heart Centre, Singapore; 3 National University Hospital, Singapore; 4 Medsavana S.L., Madrid, Spain; 5 Clinical Imaging Research Centre, National University of Singapore, Singapore; 6 Department of Cardiology, Tan Tock Seng Hospital, Singapore; 7 Universidade Federal de São Paulo, Sao Paolo, Brazil; 8 National Heart Center, Singapore; 9 Betanien Hospital, Skien, Norway; 10 Cardiovascular & Metabolic Disorders Program, Duke-National University of Singapore Medical School, Singapore; 11 Cardiovascular Research Center, College of Medical and Health Sciences, Asia University, Taichung, Taiwan; 12 Saw Swee Hock School of Public Health, National University of Singapore, Singapore; 13 Christchurch Heart Institute, University of Otago, Christchurch, New Zealand

## Abstract

**Importance:**

There are few data on remote postdischarge treatment of patients with acute myocardial infarction.

**Objective:**

To compare the safety and efficacy of allied health care practitioner–led remote intensive management (RIM) with cardiologist-led standard care (SC).

**Design, Setting, and Participants:**

This intention-to-treat feasibility trial randomized patients with acute myocardial infarction undergoing early revascularization and with N-terminal–pro-B-type natriuretic peptide concentration more than 300 pg/mL to RIM or SC across 3 hospitals in Singapore from July 8, 2015, to March 29, 2019. RIM participants underwent 6 months of remote consultations that included β-blocker and angiotensin-converting enzyme inhibitor/angiotensin receptor blocker (ACE-I/ARB) dose adjustment by a centralized nurse practitioner team while SC participants were treated face-to-face by their cardiologists.

**Main Outcomes and Measures:**

The primary safety end point was a composite of hypotension, bradycardia, hyperkalemia, or acute kidney injury requiring hospitalization. To assess the efficacy of RIM in dose adjustment of β-blockers and ACE-I/ARBs compared with SC, dose intensity scores were derived by converting comparable doses of different β-blockers and ACE-I/ARBs to a scale from 0 to 5. The primary efficacy end point was the 6-month indexed left ventricular end-systolic volume (LVESV) adjusted for baseline LVESV.

**Results:**

Of 301 participants, 149 (49.5%) were randomized to RIM and 152 (50.5%) to SC. RIM and SC participants had similar mean (SD) age (55.3 [8.5] vs 54.7 [9.1] years), median (interquartile range) N-terminal–pro-B-type natriuretic peptide concentration (807 [524-1360] vs 819 [485-1320] pg/mL), mean (SD) baseline left ventricular ejection fraction (57.4% [11.1%] vs 58.1% [10.3%]), and mean (SD) indexed LVESV (32.4 [14.1] vs 30.6 [11.7] mL/m^2^); 15 patients [5.9%] had a left ventricular ejection fraction <40%. The primary safety end point occurred in 0 RIM vs 2 SC participants (1.4%) (*P* = .50). The mean β-blocker and ACE-I/ARB dose intensity score at 6 months was 3.03 vs 2.91 (adjusted mean difference, 0.12 [95% CI, −0.02 to 0.26; *P* = .10]) and 2.96 vs 2.77 (adjusted mean difference, 0.19 [95% CI, −0.02 to 0.40; *P* = .07]), respectively. The 6-month indexed LVESV was 28.9 vs 29.7 mL/m^2^ (adjusted mean difference, −0.80 mL/m^2^ [95% CI, −3.20 to 1.60; *P* = .51]).

**Conclusions and Relevance:**

Among low-risk patients with revascularization after myocardial infarction, RIM by allied health care professionals was feasible and safe. There were no differences in achieved medication doses or indices of left ventricular remodeling. Further studies of RIM in higher-risk cohorts are warranted.

**Trial Registration:**

ClinicalTrials.gov Identifier: NCT02468349

## Key Points

**Question:**

Is remote postdischarge treatment of low-risk patients with acute myocardial infarction by a centralized nurse clinician team under physician supervision feasible and safe?

**Findings:**

In this multicenter randomized clinical trial of 301 participants, there were no significant differences in safety events, medication adjustment, or left ventricular reverse remodeling outcomes in low-risk patients with acute myocardial infarction treated for 6 months after discharge by a centralized nurse practitioner–led telehealth program compared with standard in-person care by a cardiologist.

**Meaning:**

Remote telehealth-enabled allied health care practitioner–led postdischarge management of low-risk patients with acute myocardial infarction is feasible and should be studied in higher-risk acute myocardial infarction cohorts.

## Teaser

This randomized clinical trial compares the safety and efficacy of nurse practitioner–led remote intensive management with cardiologist-led standard care for low-risk patients with acute myocardial infarction.

## Introduction

Acute myocardial infarction (AMI) is a leading cause of global morbidity and mortality.^1^ A key mechanism determining post-MI outcomes is myocardial injury leading to adverse remodeling of the left ventricle (LV), which increases the risk of heart failure and death.^2^

β-Blockers and angiotensin-converting enzyme inhibitors/angiotensin receptor blockers (ACE-I/ARBs) are beneficial after AMI,^3,4^ and adjustment of these medications to moderate to high doses is recommended in the setting of reduced LV ejection fraction (LVEF) or heart failure.^5,6,7^ Initiation and adjustment of these medications can be challenging during hospitalization, particularly among patients with borderline or low systemic blood pressure because of an emphasis on shortening length of stay and the challenges in organizing frequent face-to-face visits early after discharge.^8,9^

Telemedicine has enabled the transition from face-to-face care and is set to play a key role in the post–coronavirus disease-19 era.^10^ However, there are few randomized clinical trials on the remote management of AMI after discharge. Therefore, we evaluated the safety and efficacy of postdischarge telehealth-enabled, allied health care practitioner–led remote intensive management (RIM) of AMI.

## Methods

Improving Remodeling in Acute Myocardial Infarction Using Live and Asynchronous Telemedicine (IMMACULATE) was a multicenter randomized clinical trial of 6-month RIM compared with standard care (SC) among patients with recent AMI and who had a predischarge whole-blood N-terminal pro–b-type natriuretic peptide (NT-pro-BNP) concentration more than 300 pg/mL (trial protocol and statistical analysis plan are available in Supplement 1; eMethods in Supplement 2). The National Healthcare Group Domain Specific Review Board approved the study for all 3 hospitals (National University Heart Centre, National Heart Centre, and Tan Tock Seng Hospital in Singapore), and all participants gave written informed consent. Patients were enrolled from July 8, 2015, to March 29, 2019.

Eligible participants were randomized 1:1 to RIM or SC. Baseline cardiac magnetic resonance imaging was performed between 5 to 10 days of the index admission and repeated at 6 months (image acquisition and analysis are available in Supplement 3).

Participants randomized to RIM transmitted twice-daily blood pressure and heart rate measurements using a Bluetooth-enabled device immediately after the baseline cardiac magnetic resonance imaging (eFigure 1 in Supplement 2). Weekly consultations were conducted via telephone for 2 months and then every 2 weeks for 4 months by nurse practitioners (C.-Y Lee, J.G., C.-Y. Lo, and K.W.L.K.) who remotely adjusted ACE-I/ARBs and β-blockers according to a standardized algorithm (page 29 of the trial protocol in Supplement 1). The first measurements of serum creatinine and potassium concentration were performed at 30 days unless the nurse practitioners determined that earlier testing was required. Participants randomized to SC received regular face-to-face consultations with their cardiologists who would perform the medication adjustment.

The primary safety end point was a composite of hospitalization due to hypotension, bradycardia, hyperkalemia, or acute kidney injury. The primary efficacy end point was the indexed LV end-systolic volume (LVESV) at 6 months, adjusted for baseline LVESV. The secondary efficacy end points were LV ejection fraction and indexed LV mass at 6 months, reduction in NT-proBNP less than 20% from baseline to 6 months, difference in NT-proBNP concentration at 6 months, and β-blocker and ACE-I/ARB dose intensity at 1 month and 6 months (eMethods and eTable 1 in Supplement 2).

The mean difference in LVESV, LVEF, LV mass index, and NT-proBNP at 6 months was compared using the *t* test, and adjustment for the respective baseline measurements was made using the analysis of covariance test^11^ (Supplement 1). Stata version 16 (StataCorp) was used. Two-sided *P *values were significant at .05. Analysis began March 26, 2020.

## Results

Of 489 participants enrolled, 301 participants had NT-proBNP concentration more than 300 pg/mL and were randomized to RIM (149 [49.5%]; mean [SD] age, 55.3 [8.5] years) or SC (152 [50.5%]; mean [SD] age, 54.7 [9.1] years) (Figure). Baseline characteristics were balanced between groups with 15 patients (5.9%; 10 [7.5%] vs 5 [4.0%]) with an LVEF less than 40% (Table 1); 130 RIM participants (87.2%) and 124 SC participants (81.6%) completed both baseline and 6-month scans and had images of sufficient quality to be included in the primary efficacy analysis.

**Figure.**
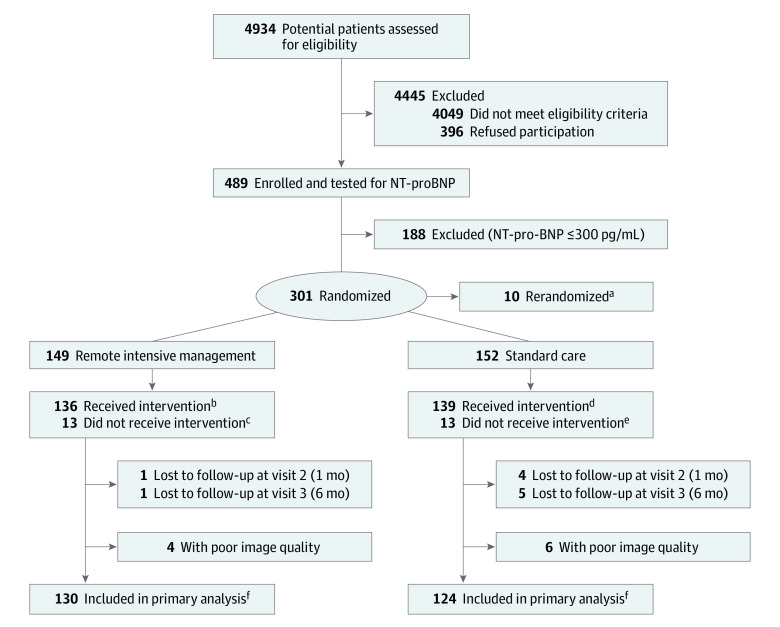
Enrollment, Randomization, and Follow-up NT-pro BNP indicates N-terminal pro–b-type natriuretic peptide. ^a^Rerandomized numbers and total randomized numbers are mutually exclusive. Ten participants had to be rerandomized because of carbon_11_–labeled acetate quality control failure in a position emission tomography substudy. ^b^A total of 136 participants successfully underwent remote intensive management after baseline cardiac magnetic resonance imaging. ^c^A total of 13 participants did not receive remote intensive management because 7 participants withdrew before baseline cardiac magnetic resonance imaging and 6 had claustrophobia. ^d^A total of 139 participants underwent standard care after baseline cardiac magnetic resonance imaging. ^e^A total of 13 participants did not receive standard care because 8 participants withdrew before baseline cardiac magnetic resonance imaging and 5 had claustrophobia. ^f^Primary analysis included participants who completed both baseline and follow-up cardiac magnetic resonance imaging scans and had images that were interpretable.

**Table 1.** Baseline Characteristics of IMMACULATE Trial Participants

Baseline characteristic	No. (%)		
	Remote intensive management (n = 149)^**a**^	Standard care (n = 152)^**a**^	Total (N = 301)^**a**^
Demographic			
Age, mean (SD), y	55.3 (8.5)	54.7 (9.1)	55.0 (8.8)
Female	8 (5.4)	8 (5.3)	16 (5.3)
Ethnicity			
Chinese	90 (60.4)	89 (58.6)	179 (59.5)
Malay	29 (19.5)	34 (22.4)	63 (20.9)
Indian	21 (14.1)	25 (16.5)	46 (15.3)
Other	9 (6.0)	4 (2.64)	13 (4.3)
BMI, mean (SD)	25.9 (4.1)	26.1 (3.9)	26.0 (4.0)
Body surface area, mean (SD), m^2^	1.8 (0.2)	1.8 (0.2)	1.8 (0.2)
Heart rate, mean (SD), bpm^b^	82.3 (16.9)	76.6 (15.1)	79.4 (16.3)
Blood pressure, mean (SD), mm Hg			
Systolic	135.2 (25.0)	132.3 (26.4)	133.7 (25.7)
Diastolic	81.9 (15.8)	79.5 (17.3)	80.7 (16.6)
Enrolling sites			
NUHCS	85 (57.1)	88 (57.9)	173 (57.5)
TTSH	42 (28.2)	42 (27.6)	84 (27.9)
NHCS	22 (14.8)	22 (14.5)	44 (14.6)
Index event			
STEMI	130 (87.3)	131 (86.2)	261 (86.7)
NT-proBNP, median (IQR), pg/mL^c^	807 (524-1360)	819 (485-1320)	808 (511-1360)
Length of stay, median (range), d	3 (1-9)	3 (1-8)	3 (1-9)
Medical history			
Myocardial infarction	9 (6.0)	13 (8.6)	22 (7.3)
CABG	0 (0.0)	1 (0.7)	1 (0.3)
PCI	9 (6.0)	13 (8.6)	22 (7.3)
Hypercholesterolemia	62 (41.6)	71 (46.7)	133 (44.2)
Diabetes	32 (21.5)	38 (25.0)	70 (23.3)
Hypertension	71 (47.7)	76 (50.0)	147 (48.8)
Current smoker	80 (53.7)	66 (43.4)	146 (48.5)
Discharge medications			
Dual antiplatelet	152 (100.0)	149 (100.0)	301 (100.0)
β-Blocker	129 (84.9)	130 (87.2)	259 (86.0)
Statin	149 (100.0)	149 (98.0)	298 (99.0)
Calcium channel blocker	4 (2.7)	3 (2.0)	7 (2.3)
ACE-I/ARB	113 (75.8)	118 (77.6)	231 (76.7)
Diuretics	6 (4.1)	5 (3.3)	11 (3.7)
MRA	12 (8.0)	14 (9.2)	26 (8.6)
Baseline CMR findings			
No.	130^d^	124^d^	254^d^
LVESVI, mean (SD), mL/m^2^	32.4 (14.1)	30.6 (11.7)	31.5 (13.0)
LVEF, mean (SD), %	57.4 (11.1)	58.1 (10.3)	57.8 (10.7)
LVEF <40%, mean (SD)	10 (7.6)	5 (4.0)	15 (5.9)
LV mass index, mean (SD), g/m^2^	74.1 (16.9)	71.6 (13.8)	72.9 (15.4)

Abbreviations: ACE-I, angiotensin-converting enzyme inhibitors; ARB, angiotensin receptor blockers; BMI, body mass index (calculated as weight in kilograms divided by height in meters squared); bpm, beats per minute; CABG, coronary artery bypass grafting; CMR, cardiac magnetic resonance; IMMACULATE, Improving Remodeling in Acute Myocardial Infarction Using Live and Asynchronous Telemedicine; IQR, interquartile range; LVEF, left ventricular ejection fraction; LVESVI, indexed left ventricular end-systolic volume; MRA, mineralocorticoid receptor antagonist; NHCS, National Heart Centre Singapore; NT-proBNP, N-terminal pro–b-type natriuretic peptide; NUHCS, National University Heart Centre Singapore; PCI, percutaneous coronary intervention; STEMI, ST-segment elevation myocardial infarction; TTSH, Tan Tock Seng Hospital.

^a^ Baseline characteristics of all randomized participants.

^b^ Three participants had missing information for heart rate.

^c^ NT-proBNP was drawn within 24 hours to 72 hours after admission but before discharge for the index acute myocardial infarction.

^d^ Baseline CMR findings of participants included in the primary analysis.

The primary safety end point occurred in 0 RIM participants and 2 SC participants (1.4%) (Table 2). Twenty-three participants experienced 23 adverse events in the RIM group, and 19 participants experienced 22 adverse events in the SC group (eTable 2 in Supplement 2). Twenty-one participants experienced 19 serious adverse events in the RIM group, and 21 participants experienced 24 serious adverse events in the SC group (eTable 3 in Supplement 2).

**Table 2.** Six-Month Effect (95% CI) of RIM vs SC on Primary and Secondary Outcomes

Variable	RIM (n = 136)^a^	SC (n = 139)^a^	Mean difference (95% CI)	*P* value
Safety end point				
Hospitalized, No. (%)				
Bradycardia	0	0	NA	NA
Hypotension	0	1 (0.7)	NA	NA
Hyperkalemia	0	0	NA	NA
Acute kidney injury	0	1 (0.7)	NA	NA
**Variable**	**RIM (n = 130)**^**b**^	**SC (n = 124)**^**b**^	**Mean difference (95% CI)**	***P *value**
**Dose intensity**				
Predictive margins for β-blocker^c^				
Month 1	2.91	2.79	0.12 (−0.02 to 0.26)	.10
Month 6	3.03	2.91		
Predictive margins for ACE-I/ARB^c^				
Month 1	2.83	2.64	0.19 (−0.02 to 0.40)	.07
Month 6	2.96	2.77		
**Efficacy end point**				
LVESVI, mean, mL/m^2^				
Unadjusted	29.8	28.7	1.05 (−2.72 to 4.81)	.58
Adjusted	28.9	29.7	−0.80 (−3.20 to 1.60)	.51
LVEF, mean, %				
Unadjusted	62.2	62.6	−0.39 (−3.21 to 2.42)	.78
Adjusted	62.5	62.1	0.40 (−1.49 to 2.29)	.68
LV mass index, mean, g/m^2^				
Unadjusted	66.9	66.3	0.54 (−3.20 to 4.27)	.78
Adjusted	65.6	67.7	−2.07 (−4.29 to 0.15)	.07
Reduction of NT-proBNP at 6 mo by at least 20% from baseline				
Unadjusted	96.3	95.4	1.25 (0.31 to 5.31)	.77
Adjusted	96.3	95.4	1.25 (0.37 to 4.22)	.72
Absolute difference in NT-proBNP at 6 mo				
Unadjusted	133.0	128.6	1.03 (0.79 to 1.35)	.80
Adjusted	131.5	130.6	1.01 (0.78 to 1.30)	.93

Abbreviations: ACE-I, angiotensin-converting enzyme inhibitors; ARB, angiotensin receptor blockers; LV, left ventricular; LVEF, left ventricular ejection fraction; LVESVI, indexed left ventricular systolic volume; NA, not applicable; NT-proBNP, N-terminal b–type natriuretic peptide; RIM; remote intensive management; SC, standard care.

^a^ Participants who were allocated to remote intensive management or standard care with any follow-up data available.

^b^ Participants who completed both cardiac magnetic resonance imaging studies and had interpretable images.

^c^ Predictive margins from mixed-effect model adjusting for baseline dose intensity score and follow-up visit.

There was no significant difference in β-blocker dose intensity at 1 and 6 months; the adjusted mean difference in β-blocker dose intensity over 6 months between RIM and SC groups was 0.12 (95% CI, −0.02 to 0.26; *P* = .10). There was a nonsignificant increase in ACE-I/ARB dose intensity with RIM over SC at 1 and 6 months; the adjusted mean difference in ACE-I/ARB dose intensity over 6 months was 0.19 (95% CI, −0.02 to 0.40; *P* = .07).

Comparing RIM vs SC, there was no significant difference in adjusted mean indexed LVESV at 6 months (28.9 mL/m^2^ vs 29.7 mL/m^2^; adjusted mean difference, −0.80 mL/m^2^ [95% CI, −3.20 to 1.60; *P* = .51]). The adjusted mean difference in 6-month LVEF and LV mass index was 0.40% (95% CI, −1.49 to 2.29; *P* = .68) and −2.07 g/m^2^ (95% CI, −4.29 to 0.15; *P* = .07), respectively. NT-proBNP reduction was not significantly different between RIM and SC groups (Table 2). Consistent findings were observed across subgroups (eFigure 2 in Supplement 2).

Remote intensive management compared with SC participants had a mean (range) of 0.67 (0-2) vs 2.70 (1-5) face-to-face visits and 17.8 (0-26) vs 0 teleconsults respectively over 6 months. The 6-month per-participant cost of RIM was 3.6-fold higher than SC ($631 vs $176), largely attributable to the high frequency of teleconsults (eMethods and eTable 4 in Supplement 2).

## Discussion

Among patients hospitalized for AMI with predischarge NT-proBNP concentration more than 300 pg/mL, RIM, consisting of frequent remote consultation and medication adjustment led by nurse practitioners, had similarly low safety events and achieved similar dose intensities of ACE-I/ARBs and β-blockers but did not improve the indexed LVESV at 6 months compared with face-to-face cardiologist-led SC.

Other trials have tested telemedicine strategies to follow up and adjust medications in patients after hospitalization for heart failure.^12^ Instead, the IMMACULATE trial tested remote intensive follow-up and drug adjustment for patients in the early post-MI period. The limited window for ameliorating adverse post-MI remodeling presents itself as a unique opportunity for more cost-effective telemedicine deployment^1^ in contrast with chronic heart failure, which requires potentially perpetual deployment of telemedicine services to prevent recurrent hospitalization over a patient’s health span.^11^ Possible explanations for the present trial’s neutral primary end point were a lower-than-expected risk of participants enrolled with relatively young age, early revascularization, and preserved LVEF.

### Limitations

Our study had several limitations. First, this trial was conducted at 3 tertiary cardiac centers, and our telemedicine unit was managed by nurse practitioners with a master’s degree in nursing and more than 10 years of nursing experience. As such, our findings may not be generalizable to allied health care professionals in other health care settings. Second, only 2 RIM participants were lost to follow-up compared with 9 SC participants, which could have biased the comparison of outcomes. Third, despite the reassuringly small number of adverse events attributable to RIM, these salutary safety signals need further validation in a larger trial of higher-risk patients with reduced LVEF or heart failure.

## Conclusions

Among patients hospitalized for AMI with elevated NT-proBNP concentration and preserved LVEF, a 6-month postdischarge multicenter RIM program by a centralized allied health care team had an equally low number of safety events and achieved similar β-blocker and ACE-I/ARB doses but did not improve LV remodeling outcomes compared with face-to-face SC by cardiologists. This feasibility study demonstrates the potential for RIM to be tested on a higher-risk AMI population with reduced LVEF or heart failure.

## Notes

Supplement 1. Trial protocol and statistical analysis plan

Supplement 2. eMethods eTable 1. Conversion of Different ACE/ARB and BB to Dose Intensity Scores eTable 2. Adverse Events of Remote Intensive Management versus Standard Care eTable 3. Serious Adverse Events of Remote Intensive Management versus Standard Care eTable 4. Per-participant Cost Analysis over 6 Months Comparing Remote Intensive Management and Standard Care eFigure 1. Telehealth Dashboard for Remote Intensive Management eFigure 2. Forest Plot for Subgroup Analysis of Primary Outcome eReferences

Supplement 3. Image acquisition and analysis

Supplement 4. Data sharing statement
