# Supplementary material for: Remote Postdischarge Treatment of Patients With Acute Myocardial Infarction by Allied Health Care Practitioners vs Standard Care: The IMMACULATE Randomized Clinical Trial
Source: JAMA Cardiol. 2020 Dec 30;6(7):1–6. doi: 10.1001/jamacardio.2020.6721 (PMC7774042; doi:10.1001/jamacardio.2020.6721)
Supplement: Supplement 1. — Trial protocol and statistical analysis plan [file jamacardiol-e206721-s001.pdf]

The IMMACULATE Trial

Improving re**M**odeling in **Ac**ute myo**C**ardial infarction **U**sing **L**ive and **A**synchronous **Te**lemedicine (IMMACULATE trial)

Principal Investigator:

Mark Y. Chan, MBBS, PhD. National University Heart Centre

Trial Statistician:

Tai Bee Choo, PhD, Saw Swee Hock School of Public Health, National University of Singapore

Study Chairman:

A. Mark Richards, MBBS, PhD, National University Heart Centre

Address: Mark Y. Chan, MBBS, PhD

Associate Professor of Medicine

Yong Loo Lin School of Medicine, National University of Singapore

Senior Consultant Cardiologist

National University Heart Centre, National University Health System

Tel: 6772 5538

Fax: 6872 2998 Email: mark_chan@nuhs.edu.sg

Summary

Our trial of a systems approach to post-MI management will test key concepts: first, whether telemedicine-based treatment intensification can minimise ventricular remodeling and hemodynamic stress through adherence to the appropriate doses of effective medications following AMI, second, if NT-pro-BNP can identify a high risk AMI subgroup that will benefit from intensified treatment. These data will serve as proof of principle for larger studies investigating the role of telemedicine-based intensified treatment in reducing death, MI and heart failure events among patients with AMI who have undergone risk stratification with NT-pro-BNP.

**Title**	**IM**proving re**M**odeling in **A**cute myo**C**ardial infarction **U**sing **L**ive and **A**synchronous **TE**lemedicine (IMMACULATE trial), a human sub study of the IMproving outcomes in acute MyocArdial infarction throUgh reversal of early and LATe cardiac remodelling (IMMACULATE) study.
**Purpose**	Comparing outcomes of ventricular remodeling and hemodynamic stress among patients with AMI and elevated NT-pro-B-type natriuretic peptide receiving telemedicine-guided post-MI treatment vs. non-telemedicine guided treatment.
**Condition**	ST-elevation and non-ST-elevation Acute Myocardial Infarction
**Principal Investigator**	**Mark Y Chan**, MBBS, PhD. National University Heart Centre, 1E Kent Ridge Road, Singapore 119228, Singapore (Overall study PI and NUH PI).
**Study sites**	**1.** National University Heart Centre<br>**2.** Tan Tock Seng Hospital<br>**3.** National Heart Centre
**Duration of the study**	~ 4 years after the approval of institutional review board (Enrollment: 2 year; Follow-up: 2years, Analysis and Publications: 1 year).
**Study Design**	**Prospective, randomized, open-labeled, blinded endpoint [PROBE] trial**.
**Eligibility**	**Inclusion criteria**<br>a. Clinically diagnosed AMI at high risk of ventricular remodeling<br>i). Clinically diagnosed STEMI or NSTEMI* within the last 7 days at high risk of ventricular remodeling<br>• Typical history of ischemic chest pain or angina equivalent symptoms (e.g. acute onset dyspnea)<br>• Typical rise or fall of cardiac enzymes with troponin I > 99th percentile (or hs Troponin T) from local clinical laboratory value<br>• ECG changes required for diagnosis of STEMI: ≥0.1mV ST segment elevation in two or more contiguous limb leads or precordial leads or presence of Q waves ≥0.02 sec in two or more contiguous limb leads or precordial leads, or new onset left bundle branch block (LBBB),<br>*The definition of STEMI and NSTEMI follows the 3rd universal definition of

	MI [19]<br><br>b. Pre-discharge NTproBNP ≥300 pg/mL for both STEMI and NSTEMI<br>c. Undergone Percutaneous Coronary Intervention (PCI) for the index event<br>d. Age =/>21 years and =/<85 years<br><br>**Exclusion criteria**<br>a. Hypersensitivity to Ticagrelor, aspirin or any excipients<br>b. Active pathological bleeding<br>c. History of intracranial haemorrhage<br>d. Bacterial Infection within 6 weeks preceding the primary angioplasty, HIV, active autoimmune disease (e.g. active SLE, active rheumatoid arthritis) or on immunosuppressive therapy (e.g. cyclophosphamide)<br>e. Women of child-bearing potential, known to be pregnant, breast-feeding, or intend to become pregnant during the study period<br>f. Malignancy within last 2 years<br>g. History of significant valvular heart disease (moderate or severe MS, MR, AS, AR, TR)<br>h. Planned CABG within the next 6 weeks<br>i. Unable to be weaned off inotropes or IABP<br>j. Active asthma or any other contraindications to beta-blockers<br>k. Arrhythmias precluding proper CMR image acquisition, such as atrial fibrillation and frequent atrial or ventricular ectopy of > 1 in 5 intrinsic QRS complexes<br>l. Contraindications to cardiac magnetic resonance imaging including claustrophobia, pacemaker or ICD implantation, mechanical valve or other metallic implants<br>m. Severe liver impairment due to chronic liver disease e.g. advanced alcoholic liver cirrhosis or primary biliary cirrhosis<br>n. Significant renal impairment (eGFR <50ml min^{-1}), end stage renal failure on renal replacement therapy<br>o. Anaemia (Hb <10 g/dL).<br>p. Psychosocial barriers to telemedicine adoption (screening for education level, dementia, substance abuse and other psychological disorders)<br>q. Participants who cannot be followed up<br>r. Participants not able or willing to consent for study.
**Experimental group and comparator**	TELEHEATH management with remote consultations X 6months (intervention) versus CONTROL (standard care) group

**Estimated enrollment**	•150 participants in the TELEHEALTH group<br>•150 participants in the CONTROL group.
**End points of the study**	**Primary efficacy endpoint**<br>- Difference in indexed left ventricular end systolic volume (LVESV) at 6 months<br>**Secondary efficacy endpoints**<br>1. CMR<br>- LVEF at 6 months<br>- LV mass index at 6 months<br>2. Frequency of participants with NTproBNP reduction of <20% over 6 months<br>3. Difference in NT-pro-BNP concentration at 6 months<br>4. BB and ACE-I/ARB dose intensity at 30 days and 6<br>5. Difference in the incidence of death, MI, stroke and heart failure at 24 months<br><br>***Primary safety endpoint***<br>- Composite of **rehospitalization** due to hypotension (systolic BP < 90 mm Hg + symptoms and signs of hypoperfusion), bradycardia (HR < 50 WITH symptoms and signs of hypoperfusion), hyperkalaemia (serum potassium >6.0mmol/L with need for cessation of ACE-I/ARB/aldosterone blockers or potassium-lowering treatment) or acute kidney injury (according to the RIFLE classification – serum creatinine increased > 2 times of baseline measurement or glomerular filtration rate decreased >50%).

Study Schedule of IMMACULATE

		V1	V2	V3	V4	F/U 5	F/U 6
Methods of assessment	Enrolment (inpt)	5-10 days (+5 days)	1 mo (± 2wks)	6mo (± 1 mo)	12mo (± 1 mo)	18 mo (± 2 mo)	24 mo (± 2 mo)
CMR/PET		T, C		T, C			
12-Lead ECG	T, C (inpt)			T, C			
Follow-up visit (Cardiologist)[1]			T, C	T, C	T, C		
Alere POC (NT-pro-BNP)	T, C		T, C	T, C	T, C		
HOMA-IR		T, C		T, C			
Research biomarkers		T, C	T, C	T, C	T, C		
Clinical bld tests[2]	T, C (inpt)		T, C (O/p)	T, C (O/p)	T, C (O/p)		
Questionnaires (self-administered)		T, C	T, C	T, C	T, C	T, C	T, C
Adverse Events Assessment		T, C	T, C	T, C	T, C	T, C	T, C
BARC Bleeding Assessment			T, C	T, C	T, C		

T = Telehealth,
C = Control

inpt = data from inpt hospital system

O/p = data from Outpatient lab tests

[1]Participants randomized to the TELEHEALTH group will have, in addition to regular visits to the cardiologist, weekly telehealth consultations with the nurse practitioner for 2 months (8 weeks), followed by fortnightly consultations for another 4 months.

[2]Fasting glucose, electrolytes, creatinine and lipid monitoring except visit 2.

[3]Ascertainment of outcomes and questionnaires will be self-administered to avoid interviewer bias. These questionnaires will be administered during the 1 week, 1 mo, 6mo and 12 mo follow-up visits. Several options will be made available to participants including web-based and mailed questionnaires (email or regular mail) for follow-up at 18- and 24-months.

TABLE OF CONTENT

1 BACKGROUND 9

1.1 The importance of Left Ventricular Remodelling in post-MI outcomes. 9

1.2 Challenges to optimal use of proven therapies in healthcare systems. 10

1.3 Life Style. 10

1.4 Intensified monitoring. 10

1.5 Telemedicine as an enabler to enhance the effectiveness of proven therapies in AMI. 11

1.6 Biomarker enrichment. 11

1.7 Biomarkers versus LVEF for initial risk stratification. 11

1.8 Summary. 12

2. STUDY HYPOTHESIS AND OBJECTIVES 12

2.1. IMMACULATE Study Objectives 12

2.2 IMMACULATE Study Hypothesis 12

2.3 Objectives and hypotheses of substudies 12

2.3.1. Effectiveness of Advanced Practice Nurse Led Telehealth on ReAdmissions and Health Related Outcomes amongst Patients Post Acute Myocardial Infarction: ALTRA Study (PI: Karen WL Koh) 12

2.3.2. Telemedicine based strategies to Increase Compliance to Antiplatelet Therapy: TICA substudy (P I: Mark Chan) 13

2.3.4. Cardiac Efficiency Substudy (PI: Grant Gullberg) 13

3. STUDY POPULATION AND PARTICIPANT SELECTION 13

3.1. Population 13

3.2. Inclusion and Exclusion Criteria 13

3.2.1. Inclusion Criteria 13

3.2.2. Exclusion Criteria 14

3.2.3. Rationale for inclusion criteria 14

3.2.4. Rationale for exclusion criteria 14

4. STUDY DESIGN AND RANDOMIZATION 15

4.1. Study Design 15

4.2. Randomization 16

4.3 Blinding. 16

5. STUDY ENDPOINTS 16

5.1 Primary efficacy endpoint 16

5.1.1 Rational for primary efficacy endpoint 16

5.2 Primary safety endpoint 17
5.2.1. Rational for primary safety endpoint 17

6 TREATMENT PROTOCOL FOR CONTROL (STANDARD CARE) AND INTERVENTION (T ELEHEALTH) GROUPS 17

6.1. Standard care (control) treatment protocol 17
6.1.1 Management of antiplatelet therapy. 17
6.1.2 Situations requiring combination antiplatelet and anticoagulant therapy 18
6.1.2.1 Left ventricular thrombus 18
6.1.2.2 Atrial fibrillation 19
6.1.3 Management of impaired left ventricular systolic function 20
6.1.4 Management of hypertension. 20
6.1.5 Management of lipids. 20

6.2. TELEHEALTH (Intervention) 20
6.2.1 Equipment and preparation: 21
6.2.1.1 TeleHealth Blood Pressure Monitoring Protocol 21
6.2.1.2 Guidelines for Setting Blood Pressure and Pulse Rate Limits 22
6.2.1.3 Termination of Blood Pressure Monitoring and Return of Devices 22
6.2.2 Telehealth remote consultation script 24

7. POST-INDEX PROCEDURE MANAGEMENT: FOLLOW-UP PHASE 34

7.1 Clinical and Laboratory follow-up 34

7.2 Study Visit procedures 34

7.3 Schedule of measurement 36

8. METHODS OF MEASUREMENT 37

8.1 Imaging 37
8.1.1 Cardiac Imaging 37
8.1.2. PET imaging (MR-PET substudy for NUH participants only) 37

9. CLINICAL EVENTS (AND DEFINITIONS) 38

10. DATA MANAGEMENT, IMAGING ANALYSIS, STATISTICAL ANALYSIS AND SAMPLE SIZE CALCULATION 38

10.1 Data Management 38

10.2 CMR Analysis 39

10.3 Statistical Analysis 39

10.4 Sample Size Calculations 39

11. SAFETY ASSESSMENT 40

11.1 Adverse Events (AE) ... 40

11.2. Serious Adverse Events (SAE) ... 40

12. EVENTS ADJUDICATION AND REPORTING ... 41

12.1. Investigator Responsibilities ... 41
12.1.1. Adverse events ... 41
12.1.2. Serious adverse events (SAE) ... 41

12.2. Sponsor or Designee's Responsibilities ... 42
12.2.1. Reporting responsibilities ... 42
12.2.2. Endpoint and SAE Adjudication ... 42

13. DISCONTINUATIONS ... 42

13.1 Participants Inadvertently Enrolled ... 42

13.2 Telehealth Intervention ... 42
13.2.1 Temporary discontinuation of Telehealth intervention ... 42
13.2.2 Permanent Discontinuation of Telehealth intervention ... 43

13.3. Ticagrelor ... 43
13.3.1 Temporary discontinuation of Ticagrelor ... 43

13.4 Discontinuation from the Trial ... 44

13.5 Participants Lost to Follow-Up ... 44

13.6 Discontinuation of Study Sites ... 45

14. STUDY OPERATIONS, MEDICAL OVERSIGHT AND RESEARCH GOVERNANCE ... 47

REFERENCES ... 48

1 Background

Acute Myocardial Infarction (AMI) accounts for more than 6,000 admissions (median length of stay 3.1 days) to Singapore hospitals each year. Contemporary treatment, including percutaneous intervention (angioplasty and stenting) and adjunctive drug therapy, has reduced early mortality from AMI. However, local 28-day AMI case-fatality in 2010 markedly exceeded the 2009 OECD average (12.7 versus 7.9%; Figs 1 and 2) despite exceptional adherence to guidelines-recommended treatment (Fig. 3) [1]. Ninety six % of patients with AMI were prescribed all 5 medications recommended by international guidelines for the management of AMI. Rates of coronary angiography and angioplasty use were also consistent with other high quality healthcare systems in economically developed countries.

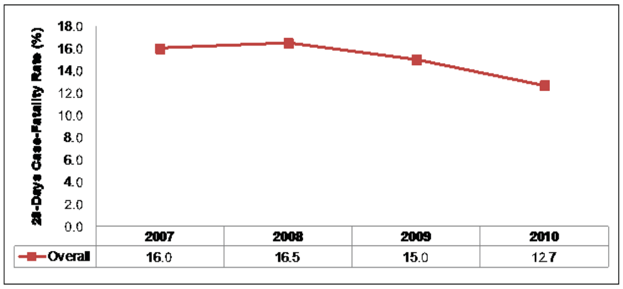

Figure 1. Singapore AMI 28-day case-fatality rates

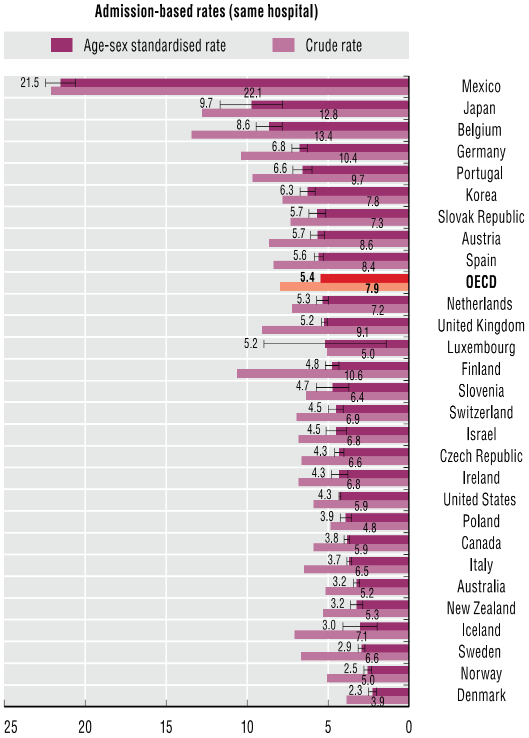

Figure 2. OECD case-fatality rates in 2009.

(Source: OECD=organization for economic cooperation and development) 2011 report.)

Secondary adverse events accrue beyond the early post-AMI period despite optimal use of procedural interventions and pharmacotherapy. Locally, the one-year incidence of cause-specific death following AMI was 15.9%, 13.9% and 10% in 2009, 2010 and 2011 respectively (*confidential privileged information, National Registry of Disease Office*). A more detailed analysis of one-year post-MI outcomes among patients hospitalized for AMI at National University Heart Centre (NUHC) in 2011-2012 revealed a one-year incidence of death and all-cause rehospitalisation of 28% with heart failure (HF) the top cause of death and rehospitalisation. In 2011-12 in ~ 800 AMI patients treated at NUHC, acute HF exceeded all other causes for re-admission in the 12 months after AMI (*confidential privileged information, National University Heart Centre, Singapore*). Explanations for this disconnect between excellent early management and worrying later outcomes in Singapore are unknown. Post-discharge adherence to treatment, life-style factors and inter-ethnic differences in the efficacy of treatment may be relevant.

1.1 The importance of Left Ventricular Remodelling in post-MI outcomes. Following infarction of the left ventricle (LV), fibroblast proliferation, increased cytokine expression, localised and systemic inflammation and increased deposition of collagen (fibrosis), lead to increased ventricular wall stress, LV dilatation and LV wall thinning (Fig.4), a process known as adverse ventricular remodeling. Using an increase in indexed LV end diastolic volume (LVEDVi) of > 20% as the definition of significant adverse remodeling, in ~30% of cases the LV remodels adversely after MI[2]. This subgroup of patients incurs disproportionately high rates of HF and death. Remodeling alters structural, metabolic and electrical elements of the heart, leading to a decline in both systolic and diastolic

function, heart valve dysfunction, arrhythmias, recurrent episodes of clinical HF and high rates of death. In vivo studies of post-MI LV remodeling in humans are limited by poor accessibility of myocardial tissue, so cardiologists must rely heavily on blood as a reporter tissue.

An unmet need exists for improved post-MI strategies to avoid LV remodeling and HF. Effective application of proven therapies is a first step. Angiotensin converting enzyme inhibitors (ACE-I), angiotensin receptor blockers (ARB), beta-blockers and mineralocorticoid receptor antagonists provide some amelioration of post-infarction LV remodeling and improve outcomes in post-MI HF[3]. Notably, early prescription of mineralcorticoid receptor antagonists for post MI LV dysfunction is supported by high quality evidence from randomized controlled trials and by authoritative guidelines which especially recommend their use in diabetic patients post-MI [4]. This is particularly relevant in Singapore where half of AMI cases have diabetes (*source: National Registry of Disease Office*). Sufficiently high doses of these drugs are necessary to maximize their anti-remodeling benefits because AMI induces an intense activation of the sympathetic and renin-angiotensin systems. However, patients often remain on the same low test doses that they are discharged with, even at 6 months post-MI [5].

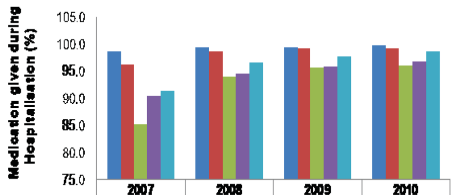

	2007	2008	2009	2010
Aspirin	98.6	99.4	99.4	99.7
Beta Blockers	96.3	98.7	99.1	99.2
Ace Inhibitors	85.2	93.9	95.6	95.9
Lipid Lowering Therapy	90.3	94.6	95.8	96.8
Other Anti-Platelet Agents	91.4	96.5	97.6	98.8

Figure 3. In-hospital prescription of guidelines recommended medications for AMI in Singapore

(Source: National Registry of Disease Office)

1.2 Challenges to optimal use of proven therapies in healthcare systems. Although we have efficacious drugs for the management of acute coronary syndrome (ACS), 3 barriers prevent us from using these drugs to effectively improve the clinical outcomes of our patients with ACS. These barriers include first, drug adherence, or patient compliance to these drugs, second, dose optimisation, or how we escalate patients to the most effective drug doses, and third, drug adverse effects management, or how we help patients deal with drug side effects. The extent of titration of ACE Inhibitors/ARBs and beta blockers to guideline-based doses and to resting heart rate may influence outcomes post-MI [5]. However, titration to effective high dose treatment is not possible during the in-hospital period because of our short average length of stay (median 4 days, interquartile range 3-5 days)[6]. Limited step-down care and other community-based resources in Singapore challenge discharge planning for acute MI. As such, current delivery of post-discharge care is episodic without consistent feedback to either patient or health care professionals (HCP) and follow-up clinic visits are scheduled months apart [7].

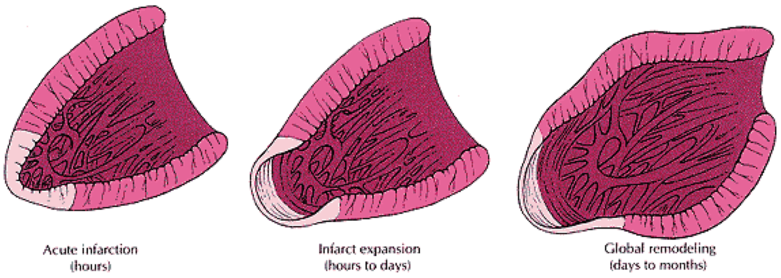

Figure 4. Temporal Evolution of Post-MI LV Remodelling

1.3 Life Style. Rates of post-MI smoking cessation, achievement of optimal exercise levels and ideal body weight are uncertain in Singapore.

1.4 Intensified monitoring. With early and recurrent contact between health care professionals and patients is in itself a powerful tool to foster adherence to therapies.

1.5 Telemedicine as an enabler to enhance the effectiveness of proven therapies in AMI. The American Medical Association has proposed telemedicine as an enabler overcoming these barriers to the effective use of otherwise efficacious drugs in our patients[8]. Many healthcare systems have adopted telemedicine to improve community outreach not only among patients in remote areas but also elderly patients in urbanized but highly congested cities (http://www.american telemed.org/about-telemedicine/what-is-telemedicine).

1.6 Biomarker enrichment. The conduct of clinical trials of patients with acute coronary syndrome (ACS) has become excessively expensive because of the large sample sizes required (http://www.manhattan-institute.org/html/fda_05.htm). Moreover, the marginal treatment effect sizes observed in these trials have led to many neutral trials in recent years. The use of biomarkers may aid patient selection so as to improve study power and enhance treatment effect size[9]. Essentially, biomarker-guided patient selection of participants for clinical trials may improve clinical trial efficiency, reduce the cost of conducting clinical trials and identify specific populations that are most likely to benefit from treatment with a novel intervention. This is aligned with the concept that a sustainable drug discovery ecosystem must shift from a blockbuster model to niche drugs personalized for specific populations.

There is a clear association between plasma peptide levels and cardiac structure/function, presaging prognosis after AMI[10]. Data from a carefully annotated and well-published AMI cohort recruited in the 1990's (the "Post Myocardial Infarction" Study; n=1,100) [11]. The median plasma concentration of NTproBNP at 30 days after symptom onset in AMI was approximately 660 pg/ml. The half of the AMI population with early levels above 660 pg/ml incurs 85% of all subsequent post-MI deaths and heart failure events post-MI. Furthermore, patients in whom early NTproBNP levels greater than 660 pg/ml fail to fall by 20% or more in the 4 months following AMI (~50%) incur 3-fold the 2 year rates of death and HF than observed in patients with falls of >20% in peptide concentrations (data on file). These powerful associations underpin our decision to use NTproBNP as a biomarker to identify post-MI patients at high risk of LV remodeling in our study.

In our own local series of patients with AMI, we observed that median NT-proBNP (whole blood Alere Triage assay), median NT-proBNP was ~300 pg/ml (data on file). We attribute the lower median NT-proBNP to the following reasons: better contemporary treatment, including more rapid reperfusion with PCI, whole blood assays showing generally lower values than plasma assays because of red blood cell volume effect and possible ethnic differences in NT-proBNP. Nonetheless, the NT-proBNP level of 300 pg/ml coincides with the diagnostic threshold of NT-proBNP for acute heart failure, giving us confidence that a NT-proBNP threshold of 300 pg/ml will significantly enrich our trial population for LV remodeling.

1.7 Biomarkers versus LVEF for initial risk stratification. NTproBNP has several advantages over LVEF for the initial risk stratification of AMI: 1. NTproBNP is a highly reproducible immunoassay while LVEF estimation is subjective because most echocardiographic laboratories assess LVEF visually[12]; 2. NTproBNP is highly quantitative with a far greater dynamic range than LVEF and 3.while LVEF only reflects left ventricular systolic function, NTproBNP compositely measures multiple high-risk features including left ventricular systolic function, left ventricular diastolic function, intraventricular pressure, renal function and systemic hemodynamic stress[13]. Presently, LVEF<40% is primary criteria for intensifying treatment with RAS and beta-blockers after an AMI[14-16].

Yet, echocardiographic assessment of LVEF requires skilled image acquisition and interpretation; LVEF is estimated visually in clinical practice, leaving much room for subjective error. In contrast, NT-pro-BNP is now measurable using whole blood point-of-care devices that remove the element of subjective interpretation. We hypothesize that point-of-care NT-pro-BNP measurements (http://www.alere.com/us/en/product-details/triage-nt-probnp.html) will successfully identify patients with AMI who will benefit from intensification of treatment with RAS blockers and beta-blockers, paving the way for a simple and objective alternative to assess the risk of future ventricular remodeling and heart failure.

1.8 Summary. Our trial of a systems approach to post-MI management will test several novel concepts: first, whether telemedicine can improve ventricular remodeling and hemodynamic stress through adherence to the appropriate doses of effective medications following AMI, second, if NT-pro-BNP can identify a high risk AMI subgroup that will benefit from intensified treatment. These data will serve as proof of principle for larger studies investigating the role of telemedicine-based intensified treatment in reducing death, MI and heart failure events among patients with AMI who have undergone risk stratification with NT-pro-BNP.

2. Study Hypothesis and Objectives

2.1. IMMACULATE Study Objectives

IMMACULATE is a randomised controlled trial to test the superiority of telemedicine-guided management (intervention, n=150) versus standard care (control, n=150) in improving outcomes of participants with AMI identified to be at a high risk of ventricular remodeling and heart failure by elevated plasma NT-pro-BNP. The trial will focus upon live and asynchronous strategies to achieve dose optimization and sustained implementation of proven pharmacotherapies and lifestyle measures in the post-discharge period. The primary efficacy endpoint will be LVESV at 6 months, as measured using cardiac magnetic resonance imaging. The secondary efficacy endpoints included the following: reduction in NT-pro-BNP <20% from baseline to 6 months, NT-pro-BNP concentration at 6 months, LVEF, indexed LV mass index at 6 months and BB and ACE-I/ARB dose intensity at 30 days and 6 months.

2.2 IMMACULATE Study Hypothesis

Hypothesis: Among participants with AMI identified to be at a high risk of left ventricular remodeling and heart failure by elevated plasma NTproBNP ≥ 300 pg/ml, 6 months of telemedicine-guided post-discharge management will minimize cardiac left-ventricular remodeling as compared with standard care. We secondarily hypothesize that a telemedicine-based algorithm will reduce hemodynamic stress, defined as a lower frequency of participants with NTproBNP reduction of <20% over 6 months, as compared with standard care.

2.3 Objectives and hypotheses of substudies

2.3.1. Effectiveness of Advanced Practice Nurse Led Telehealth on ReAdmissions and Health Related Outcomes amongst Patients Post Acute Myocardial Infarction: ALTRA Study (PI: Karen WL Koh)

Objectives: To assess the efficacy of a 6-month telemedicine-based program in reducing unplanned readmissions during the same 6-month period among participants discharged from hospitalization for a recent AMI at 30 days and 180 days timepoints. The end-points would be measured in readmission days per 1000 follow up days. Secondary endpoints include the assessment of cardiovascular versus non-cardiovascular readmissions for the duration of 12 months, emergency department visits and health-related outcomes such as cardiac self-efficacy,

cardiovascular risk factors, quality of life, anxiety and depression at various timepoints.
Hypothesis: A 6-month telemedicine-based program reduces the incidence of all-cause unplanned readmission among participants with a recent AMI, as compared with standard care.

2.3.2. Telemedicine based strategies to Increase Compliance to Antiplatelet Therapy: TICA substudy (PI: Mark Chan)

Objectives: To assess the efficacy of a telemedicine-based coaching program in improving adherence to dual antiplatelet therapy. Endpoints assessed will be longitudinal profiles of adenosine diphosphate–induced impedance aggregometry (http://www.cobas.com/home/product/hemostasis-testing/multiplate-analyzer.html), the 8-point Morisky questionnaire[17], pill counting (Ticagrelor only) and bleeding academic research consortium (BARC) bleeding endpoints[18]. To remove the cost of medication as an impediment to Ticagrelor adherence, we will supply 12 months of Ticagrelor at no cost to all patients in the study.
Hypothesis: Telemedicine-based coaching improves adherence to dual antiplatelet therapy leading to superior platelet reactivity outcomes, as compared with standard care.

2.3.3. Cardiac Efficiency Substudy (PI: Grant Gullberg)

We will assess the effectiveness of telemedicine-guided management, as compared with standard care, in improving myocardial oxygen utilization assessed using hybrid magnetic resonance imaging positron emission tomography.

3. Study Population and Participant Selection

3.1. Population

The study population will consist of approximately 300 to 340 participants with STEMI and NSTEMI at high risk of ventricular remodeling and heart failure.

3.2. Inclusion and Exclusion Criteria

3.2.1. Inclusion Criteria

a. Clinically diagnosed STEMI or NSTEMI* within the last 7 days at high risk of ventricular remodeling

- Typical history of ischemic chest pain or angina equivalent symptoms (e.g. acute onset dyspnea)
- Typical rise or fall of cardiac enzymes with troponin I > 99th percentile (or hs Troponin T) from local clinical laboratory value.
- ECG changes required for diagnosis of STEMI: ≥0.1mV ST segment elevation in two or more contiguous limb leads or precordial leads or presence of Q waves ≥0.02 sec in two or more contiguous limb leads or precordial leads, or new onset left bundle branch block (LBBB),

*The definition of STEMI and NSTEMI follows the 3rd universal definition of MI [19]

b. Pre-discharge NTproBNP ≥300 pg/mL for both STEMI and NSTEMI

c. Undergone PCI for the index event

d. Age =/>21 years and =/<85 years

3.2.2. Exclusion Criteria

a. Hypersensitivity to Ticagrelor, aspirin or any excipients
b. Active pathological bleeding
c. History of intracranial haemorrhage
d. Bacterial infection within 6 weeks preceding the primary angioplasty (e.g. community acquired pneumonia), HIV, active autoimmune disease (e.g. active SLE, active rheumatoid arthritis) or on immunosuppressive therapy (e.g. cyclophosphamide)
e. Women of child-bearing potential, known to be pregnant, breast-feeding, or intend to become pregnant during the study period
f. History of malignancy within the last 2 years
g. History of significant valvular heart disease (moderate or severe MS, MR, AS, AR, TR)
h. Planned CABG within the next 6 weeks
i. Unable to be weaned off inotropes or IABP
j. Active asthma or any other contraindications to beta-blockers
k. Arrhythmias precluding proper CMR image acquisition, such as atrial fibrillation and frequent atrial or ventricular ectopy of > 1 in 5 intrinsic QRS complexes
l. Contraindications to cardiac magnetic resonance imaging including claustrophobia, pacemaker or ICD implantation, mechanical valve or other metallic implants
m. Severe liver impairment due to chronic liver disease e.g. advanced alcoholic liver cirrhosis or primary biliary cirrhosis
n. Significant renal impairment (eGFR<50 ml min^{-1}), end stage renal failure on renal replacement therapy
o. Anaemia (Hb<10 g/dL).
p. Psychosocial barriers to telemedicine adoption (screening for education level, dementia, substance abuse and other psychological disorders)
q. Participants who cannot be followed up
r. Participants not able or willing to consent for study.

3.2.3. Rationale for inclusion criteria

A key inclusion criterion is an elevated whole blood NT-pro-BNP ≥300 pg/mL for STEMI and NSTEMI. We have found whole blood NT-proBNP measurements performed on the Alere Triage system to be 20-40% lower than plasma NT-proBNP measurements performed on the Cobas Eclsys system (data on file). In general NT-proBNP measurements at 24-96 hours are higher among patients with NSTEMI than STEMI, so a higher NT-proBNP threshold for NSTEMI would increase the likelihood of selecting NSTEMI patients at increased risk of ventricular remodeling. Data points to a far greater incidence of ventricular remodeling among patients with substantial elevations of both NT-proBNP and cardiac troponin in the early post-infarction period [20].

3.2.4. Rationale for exclusion criteria

Antiplatelet therapy with Ticagrelor reduced mortality as compared with Clopidogrel in a large randomized trial of patients with acute coronary syndrome[21].As such, Ticagrelor is now the preferred $P2Y_{12}$ antagonist for the management of acute coronary syndrome and will be included as part of excellent background therapy in the

IMMACULATE trial.

4. Study design and randomization

4.1. Study Design

Figure 5. Prospective, randomized, open-labeled, blinded endpoint [PROBE] trial

Participant with STEMI or NSTEMI

↓

Screened for eligibility

↓

Enrollment of Participant after obtaining Informed consent

↓

Pre-discharge NTproBNP ≥ 300 pg/mL for both STEMI and NSTEMI*

↓

Randomize

↓ ↓

TELEHEALTH **CONTROL**

↓ ↓

CMR at 5 -10 days post-PCI and 6 months from date of admission (primary endpoint). Study visits and up to 31 mls blood collected at 1 week (baseline upon enrolment during CMR), 1month, 6months, and 1 year. Event follow-up, Quality of Life, clinical review at 30 days,1 month, 6months, and 1 year. Clinical event follow up for all participants up to 2 years (survey only, no clinic visits). Adverse events will be assessed at every follow up.

4.2. Randomization

After confirmation of an NTproBNP (*Alere POC*) measurement ≥ 300 pg/mL, participants are randomized into the TELEHEALTH versus CONTROL group (1:1 sequential block randomization blocks of 4 and 6). Randomization will be done via emailing system, as the study coordinators are blinded from the randomization sequences.

Participants entered into the study may not be enrolled (that is, randomly assigned to a treatment group) if they:

- Are discovered to have not met all the inclusion criteria.
- Have one or more exclusion criteria present at the time of randomization.

Participants not enrolled (randomized) in the study should be discontinued from further participation in the study and will be classified as screen failures. No further follow-up is required. Non-enrolled Participants will not be included in the intent-to-treat (ITT) population.

4.3 Blinding.

Pre-allocation (randomisation master list) to Telehealth-guided management or control will be concealed from the PI and CRC at each site. However, blinding of participants, CRC, the Telehealth team and other healthcare workers is not possible in a telemedicine trial which is by default an open-label trial design. To protect against bias, we will implement the following measures: 1. Blinding to treatment allocation, of imaging team performing core analysis and interpretation of cardiac MRI images 2. Blinding of statistical team to treatment allocation and 3. Blinding of NT-pro-BNP results to the Telehealth team and healthcare workers.

5. Study Endpoints

5.1 Primary efficacy endpoint

- Difference in LVESV at 6 months as measured by CMR (ventricular remodeling endpoint)

Secondary efficacy endpoints

1. CMR
- LV remodeling index at 6 months
- Infarct size (grams and percentage of total LV mass) at 6 months
2. Frequency of participants with reduction in NTproBNP <20% from baseline to 6 months (hemodynamic stress endpoint)
3. Difference in NTproBNP level at 6 months
4. BB and ACE-I/ARB dose intensity at 30 days and 6 months (dose intensity scores will be calculated by converting equivalent doses of each BB and ACE-I/ARB to an ordinal scale from 0 to 5)
5. Difference in the incidence of death, MI, stroke and heart failure at 24 months.

Exploratory secondary endpoints

Other exploratory secondary endpoints include systolic BP (SBP) and diastolic BP (DBP), LDL-C and, among patients with diabetes mellitus, HbA1c

5.1.1 Rational for primary efficacy endpoint

The ultimate goal of introducing technology-driven strategies in healthcare is to improve clinical outcomes. However, a randomized trial with clinical outcomes as the primary endpoint will require a sample size in the thousands. As such, we have selected a more efficient surrogate endpoint, left ventricular remodeling, which has been long established as an independent predictor of clinical outcomes in AMI and often used as a surrogate efficacy outcome in clinical trials of AMI[22].

5.2 Primary safety endpoint

Composite of **rehospitalization** due to hypotension, (systolic BP < 90 mm Hg + symptoms and signs of hypoperfusion), bradycardia (HR < 50 WITH symptoms and signs of hypoperfusion), hyperkalaemia (serum potassium >6.0mmol/L with need for cessation of ACE-I/ARB/aldosterone blockers or potassium-lowering treatment) or acute kidney injury (according to the RIFLE classification – serum creatinine increased > 2 times of baseline measurement or glomerular filtration rate decreased >50%).

5.2.1. Rational for primary safety endpoint

A major concern with remote titration of drug therapy is inadequate patient monitoring leading to drug side effects. Both ACE-I/ARB and betablockers can lower blood pressure and/or heart rate, especially during the early post-infarct period. Moreover, ACE-I/ARB can increase serum potassium levels and impair renal function.

Note: Substudy endpoints are described in their individual protocols.

6 Treatment protocol for control (standard care) and intervention (telehealth) groups

Both control and intervention groups will receive a very high level of standard care: 1-year of dual antiplatelet therapy with aspirin and Ticagrelor, moderate/high dose statins, cardiac rehabilitation, smoking cessation and regular face-to-face clinic consultations. We have designated this level of care in the control group as excellent standard care that exceeds the standards of not only all the restructured hospitals in Singapore but also centres of excellence in other countries. In essence, this trial will delineate the true benefit of telemedicine-guided management, as we will be comparing telemedicine against excellent standard care.

6.1. Standard care (control) treatment protocol

The following interventions will be uniformly applied to both the standard care and telehealth groups. These interventions were developed following international guidelines for post-MI management [3, 23, 24].

6.1.1 Management of antiplatelet therapy. All participants will receive dual antiplatelet therapy with 1. Aspirin 300 mg loading dose followed by 100 mg daily and 2. Ticagrelor 180 mg loading dose followed by 90 mg twice a day (BD). Ticagrelor will be provided free-of-charge for 12 months for all study participants. If patients are on another $P2Y_{12}$ antagonist, either Clopidogrel or prasugrel, prior to receiving Ticagrelor, we will stop the preceding $P2Y_{12}$ antagonist and start Ticagrelor prior to the first CMR. Within 24 hours of stopping Clopiodgrel or Prasugrel, Ticagrelor 90 mg is served, without a loading dose and continued at 90 mg BD. If Clopidogrel or Prasugrel is stopped for > 24 hours, a loading dose of Ticagrelor 180 mg is served followed by Ticagrelor 90 mg BD.

Figure 6. Management of antiplatelet therapy in both standard care and telehealth groups

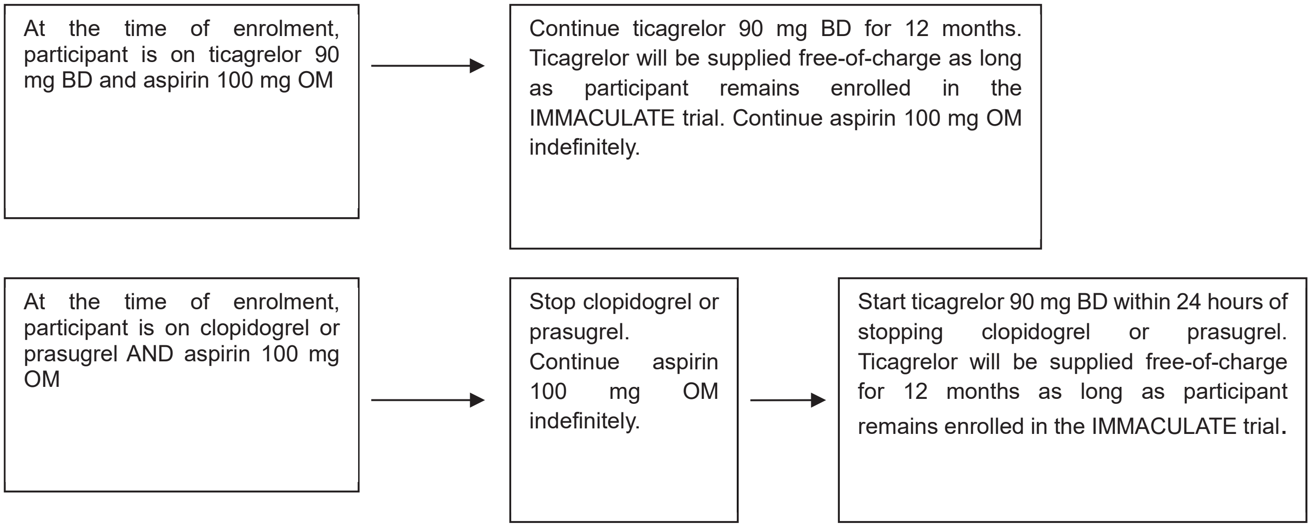

6.1.2 Situations requiring combination antiplatelet and anticoagulant therapy

Two groups of patients who may require combination therapy with antiplatelet and anticoagulant therapy are:

1. Participants who develop left ventricular thrombus after enrolment (figure 7)

2. Participants who develop atrial fibrillation after enrolment (figure 8)

In both situations, we strongly encourage that patients remain enrolled in the IMMACULATE trial. The following describes the protocol for managing participants with left ventricular thrombus and/or atrial fibrillation.

6.1.2.1 Left ventricular thrombus

For participants taking clopidogrel at the time that a left ventricular thrombus is detected, both aspirin and clopidogrel are continued (figure 7). For participants taking prasugrel or ticagrelor at the time that a left ventricular thrombus is detected, prasugrel or ticagrelor will be discontinued and aspirin continued; clopidogrel will then be initiated at a loading dose of 300 mg followed by 75 mg daily.

Patients will be prescribed low molecular weight heparin, adjusted to body weight and creatinine clearance, as per the managing physician's discretion in discussion with the site PI. Warfarin, targeting an international normalized ratio of 1.8-2.5 will be started and continued until the 2nd MRI at 6 months. Low molecular weight heparin will be stopped when the INR is > 1.8. After one month, aspirin is discontinued. At 6 months, CMR will be performed to assess resolution of left ventricular thrombus. If the CMR at 6 months shows resolution of left ventricular thrombus, warfarin is discontinued and the INR is repeated at 5-7 days after discontinuation of warfarin; if the INR is <1.8, aspirin and ticagrelor is initiated at a dose of 90 mg BD without a loading dose. If the CMR at 6 months shows persistence of left ventricular thrombus, longer-term anticoagulant therapy will be decided at the discretion of the site PI.

(Figure 7. Management of participants with LV thrombus)

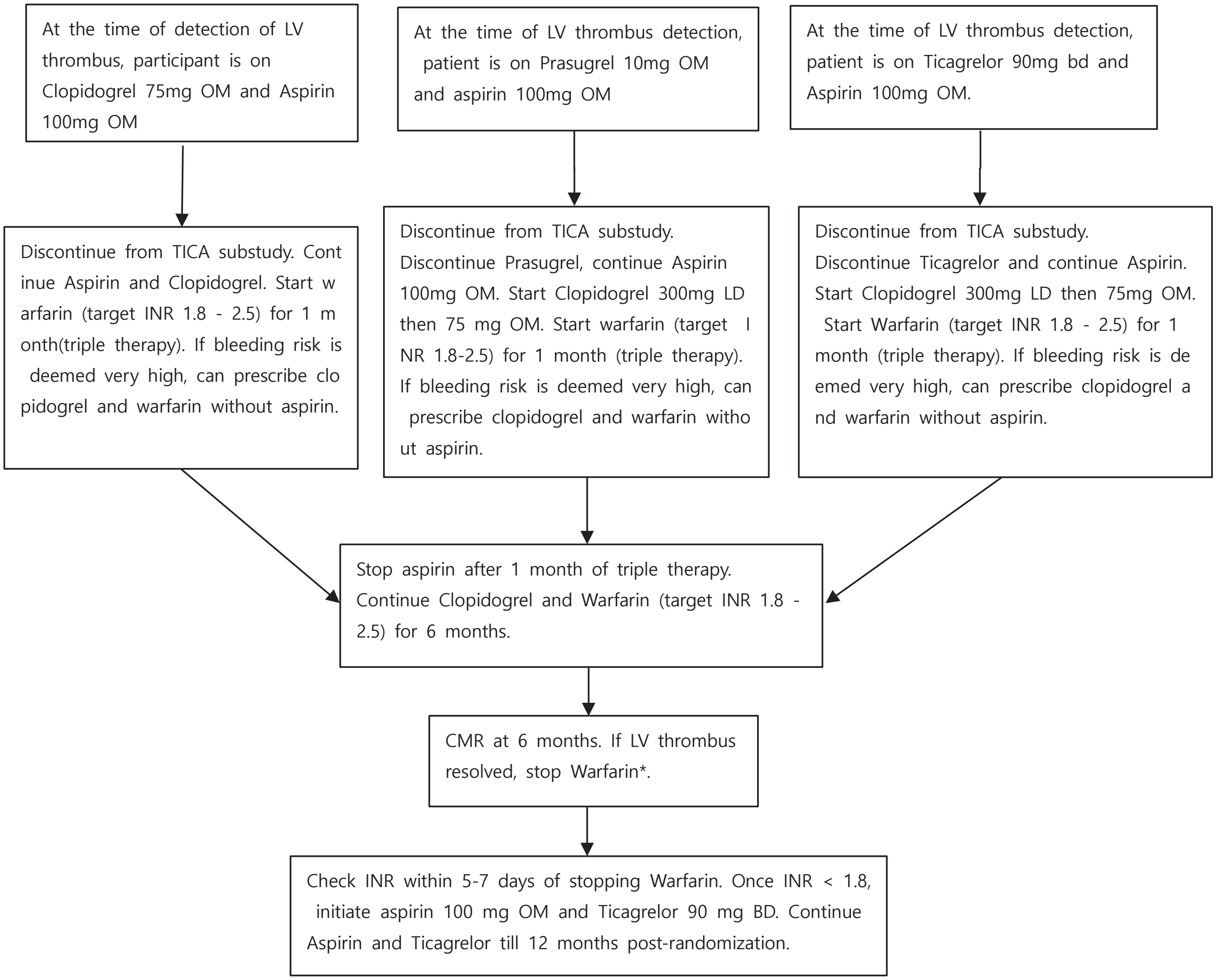

*Site PI and co-investigators have the option of discontinuing warfarin before six months, e.g. following an interim echocardiogram at 4 months that shows resolution of the LV thrombus. In such an instance, the INR should be checked upon stopping warfarin and when it is <1.8, clopidogrel is stopped and the patient is continued on aspirin 100 mg OM and ticagrelor 90 mg BD.

6.1.2.2 Atrial fibrillation

If the participant develops atrial fibrillation and deemed to require anticoagulation (i.e. CHA2D2-Vasc score > 1), ticagrelor is discontinued, clopidogrel is started (300 mg loading dose followed by 75 mg daily) and warfarin is started, targeting an international normalized ratio of 1.8-2.5 (figure 8). The site PI may or may not choose to overlap warfarin with low molecular weight heparin. The site PI may also elect to prescribe only clopidogrel + warfarin as in the WOEST trial, without aspirin, or prescribe rivaroxaban in combination with aspirin and/or clopidogrel as in the PIONEER-AF trial.

6.1.3 Management of impaired left ventricular systolic function

As per international guideline recommendations, patients in both standard care and telehealth groups will receive ACE inhibitors/ARBs and beta-blockers to ameliorate adverse left ventricular remodeling [3, 16].

6.1.4 Management of hypertension. The overall approach in managing hypertension will be to ensure that all participants are treated with a beta-blocker and/or an ACE inhibitor or if intolerant, an angiotensin receptor blocker (ARB) at adequate dose. Additional therapies will be added sequentially to achieve a BP less than 140/90.

(Figure 8. Management of participants with AF)

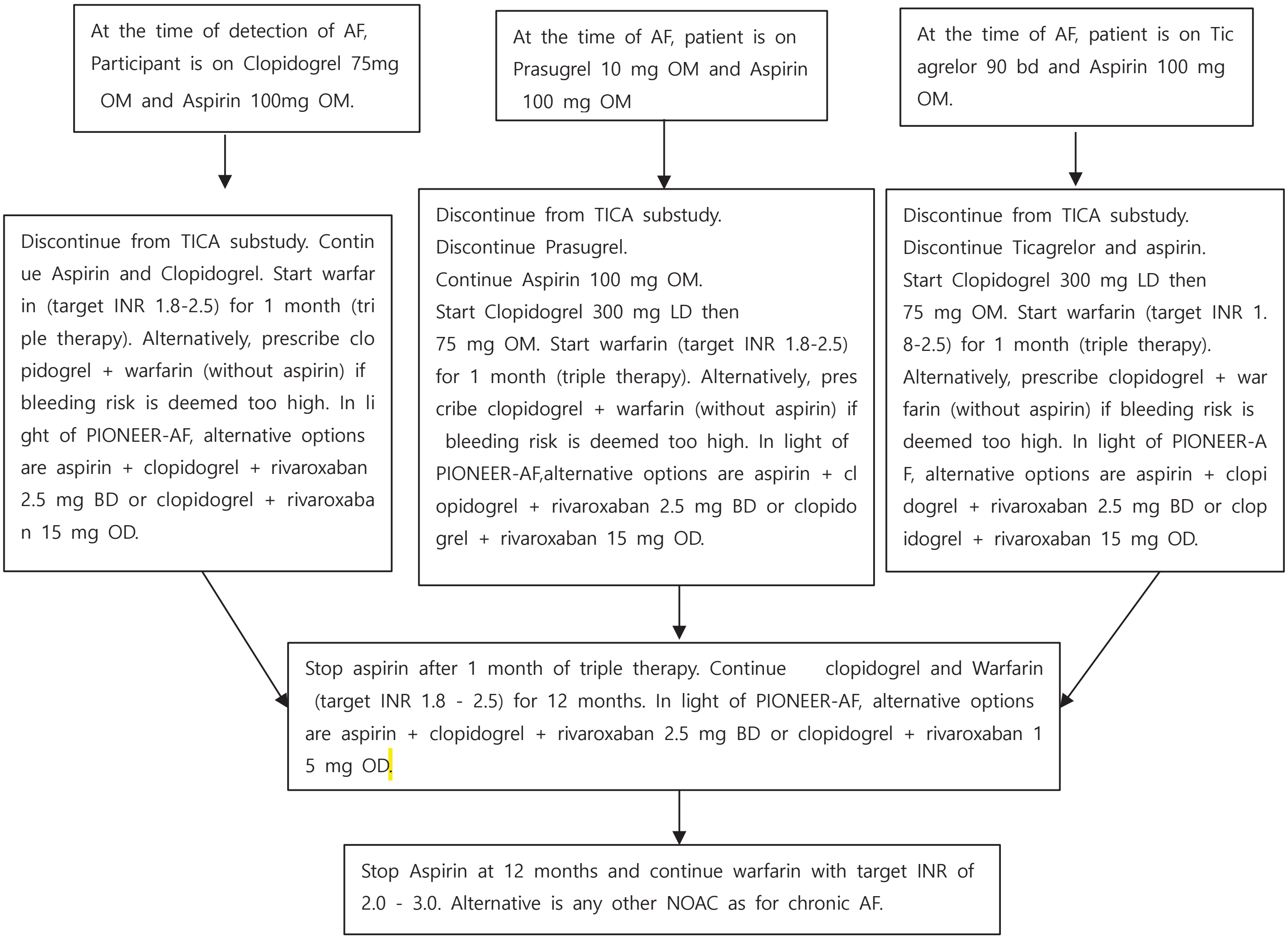

6.1.5 Management of lipids. The overall approach in lipid management will be to use moderate-to-high intensity statins in all participants (e.g. simvastatin 40 mg, atorvastatin 40 mg or rosuvastatin 20 mg). A secondary target will be to reach LDL cholesterol <80 mg/dl.

6.2. TELEHEALTH (Intervention)

All participants randomized to the TELEHEALTH arm will follow, in addition to standard care as described above,

a 6-month intervention protocol developed in accordance with the Singapore national telemedicine guidelines (https://www.moh.gov.sg/content/dam/moh_web/Publications/Guidelines/MOH%20Cir%2006_2015_30Jan15_Telemedicine%20Guidelines%20rev.pdf). The protocol comprises the following:

1. Daily blood pressure monitoring with the Blood pressure Meter, commencing at the point of discharge. Blood pressure and heart rate are closely monitored during the first 2 months (8weeks) of drug titration.
2. For the 1st and 2nd month, remote consultations will focus on titration of ACE-I/ARB and beta blockers according to the stepped care algorithm, monitoring of symptoms, blood pressure and heart rate, and coaching participant adherence to medication and lifestyle measures.
3. From the 3rd to 6th month, once patients have achieved optimal doses of ACE-I/ARB and beta blockers in the 1st 2 months of the intervention, remote consultations will focus on monitoring of symptoms and maintaining adherence to medication and lifestyle measures (adherence coaching).
4. The frequency of consultations will be weekly remote consultation for 8 weeks (1st and 2nd month) commencing immediately after the 1st CMR scan, then remote consultation every 2 weeks from months 3-6.
5. Throughout the entire 6 months, the TELEHEALTH team will manage abnormal symptoms and escalate abnormal symptoms appropriately to the cardiologist-in-charge.

6.2.1 Equipment and preparation:

1. Each enrolled participant randomized to the TELEHEALTH group will receive a blood pressure meter at the time of discharge. The study coordinator will train participants on proper use of the blood pressure system, with an onsite trial run of the participant's competency in measurement and transmission of data.
2. Heart Recovery manual will be given to the patient.
3. Following an initial run-in period with ACE-I and beta blockers, patients randomized to telehealth-guided management will be prescribed a full month of ACE-I and betablockers at the maximum dose but in the lowest dose denomination (e.g. 2.5 mg bisoprolol tablets instead of 5 or 10 mg bisoprolol tablets).

6.2.1.1 TeleHealth Blood Pressure Monitoring Protocol

- The service is provided only during office hours (9 am-6 pm Monday to Friday).
- Participants who meets the above inclusion criteria will be enrolled by the Study coordinator
- There must be a cardiologist in-charge of the participant; duties of the cardiologist are as follows:
  - Provide advice and guidance to the Advanced Practiced Nurse (APNs) and other clinical staff involved in the service
  - Be kept informed of alerts, interventions and participant symptoms
  - Make clinical management decisions for the participants
- Responsibility for participants enrolled into the Telehealth arm is indicated below:
  - Informed consent will be taken from the participants by the study coordinator before enrolment and the informed consent process will be documented in the case notes and/or CDOC
- Participants and/or their care-givers will be counselled on the following upon enrolment into the Telehealth arm:
  - How to use the BP device and how to measure and record the participants' BP
  - How to maintain the equipment
  - Under what circumstance an APN will call the participant
  - Duration of the service – TWO months
  - When and how to return the equipment (signing of device-loan form)

  - Liability in case of damage to the equipment
  - Follow-up appointments
- Patient Information Leaflet (PIL) will be given to all enrolled participants
- Participants and/or care-givers will also be provided with telephone numbers to call:
  - For technical assistance with the measuring devices [number:6632 1432], Mon to Fri, 8am to 8pm
  - In the event of new or worsening symptoms/signs [number: 97336307], Mon to Fri, 9am to 6pm

6.2.1.2 Guidelines for Setting Blood Pressure and Pulse Rate Limits

- For the purpose of drug titration to achieve optimal doses of ACE-I/ARB and betablockers, we have stipulated a minimum SBP of110mmHg for the blood pressure device.
- For **resting** pulse rates, we have stipulated a minimum of 55bpm and maximum of 100bpm

6.2.1.3 Termination of Blood Pressure Monitoring and Return of Devices

- Participant and/or care-giver will be informed of the monitoring termination date (following 8 weeks of drug titration) by the Telehealth team/ APNs
- Devices will be returned to coordinator at the participant’s stipulated clinic review date
- Participants will sign off device loan form upon returning device to study coordinator.
- Main site coordinator will de-allocate device in Telehealth System.
- Telehealth consultations will continue via telephone from months 3-6.

Figure 9. Algorithm for participants randomized to the TELEHEALTH arm

Patient identification

- Patient enrolled into IMMACULATE and randomized to TELEHEALTH arm
- NHC & TTSH clinical research coordinator informs central coordinator @ NUH

↓

Local coordinator responsible for enrolment & registration at each hospital

- Check BP machine, distribute equipment to patient/care-giver & teach them how to use/care for the devices
- Hand out patient information leaflet & educate patient about self-management using “Heart Recovery” and BP circumstances in which APN will call patient
- Patient to sign device-loan form
- Activate patient in Telehealth system (done by main site coordinator).
- Inform patient of return date and venue (after 8-weeks’ titration)
- Document the above in CDoc under patient education
- Study coordinator will HMS **APN @97336307** to inform of patient’s enrolment
- Document patient consent & enrolment procedure above in CDoc

↓

NUH APN responsible for all hospitals

Advanced Practice Nurse (Cardiology, NUH)

↓

Daily check on participants’ BP in Telehealth system. Call patient if no BP recorded for 3 consecutive scheduled readings at next available working day. Attend to unscheduled calls from patient about symptoms or equipment (9am-6 pm)

↓

Weekly remote consultations X 8weeks then every 2 weeks for 4 months following remote consultation script

↓

APN’s activities

Titration of ACE/ARB and Beta-blockers according to stepped care (dose titration) algorithm for 8 weeks. Increase ACE-I/ARB and beta blocker drug dose only from Monday to Thursday so as to allow a minimum of 24 hours of monitoring before the weekend. Document dose titration in electronic medical records. Inform cardiologist at respective sites to amend medications in electronic prescription. BP set is loaned to patients during this 8-week period, following which the BP set is returned to the clinical research coordinator. Further consultations are conducted via telephone.

↓ (loops back to daily check)

Appropriate management and escalation for abnormal symptoms according to symptom management and escalation protocol

*Alerts set in the TeleMetrix system will include:

1. No BP recorded for ≥3 consecutive readings at the next available working days
2. BP outside of set limits (individualized to each patient)

6.2.2 Telehealth remote consultation script

All participants randomized to the telehealth arm will receive weekly remote titrations and consultations X 2 months followed by remote consultations every 2 weeks for 4 months. A centralized telehealth team of NUH advanced nurse practitioners will provide this service to all participating patients to ensure a standardized intervention for all participants. All changes in medication or medication doses must be communicated to the respective hospital PIs [Mark Chan (NUH), Ho Hee Hwa (TTSH) and Derek Hausenloy (NHC)] or their co-investigators, who will document changes in the electronic prescription system. For matters outside the scope of the protocol, e.g. rescheduling of appointments, lost-and-found, patient bill, etc. participants will be gently referred to the appropriate service. Each telehealth consultation should last no more than 20-30 minutes.

Week 1 - 8

1. Introduce yourself

"Good _______ , I am nurse ______________ from National University Hospital Telehealth team. May I speak to _______________ (participant's name)?"

2. Tell the person purpose of call:

"I am calling to follow up on your heart condition after discharge and to help manage your medications."

a. "How are you feeling?" If well, proceed to step 3.

b. If unwell, take note of Participant's complaint and investigate cause where possible and note down for documentations – refer to "*Signs and Symptoms management*" protocol

c. If non-critical, "I will inform your doctor in charge and get back to you shortly."

d. If critical, "based on what you just informed me, I would like to advise you to go to the nearest clinic or to the nearest emergency department. In the mean time, I will inform your doctor. "

3. Assess medication non-adherence, side effects and manage non-adherence.

a. Assess medication non-adherence. Recognise that non-adherence is common and that most Participants are non-adherent sometimes. Routinely assess adherence in a non-judgemental way with each telemedicine consultation. Consider assessing non-adherence by asking the Participant if they have missed any doses of medicine recently. Make it easier for them to report non-adherence by:

- asking the question in a way that does not apportion blame. E.g. "Can I please check how you have been taking your medications?"
- explaining why you are asking the question e.g. "This is because some of our participants have trouble taking their medications regularly. The medications prescribed are very important as they help to heal your heart and prevent another heart attack".

- mentioning a specific time period e.g. 'in the past week'
- asking about medicine-taking behaviours such as reducing the dose, stopping and starting medicines.
- Consider using records of prescription re-ordering, pharmacy patient medication records and return of unused medicines to identify potential non-adherence and participants needing additional support.

b. Identify reasons for non-adherence, including medication side effects. If a participant is not taking their medicines, discuss with them whether this is because of beliefs and concerns or problems about the medicines, such as known or perceived side-effects (intentional non-adherence) or because of practical problems (unintentional non-adherence).
- e.g. of questioning to elicit personal beliefs “do you think that any of your medications are not helpful or perhaps even harmful to you?”
- e.g. of questioning to elicit medication side effect “Are you experiencing any shortness of breath that occurs even at rest and does not get worse with exertion (Ticagrelor)? Do you bruise easily (Ticagrelor)? Are you experiencing a troublesome dry cough (ACE-I)?

c. Develop a strategy with the participant to deal with non-adherence
- Address any beliefs and concerns that participants have that result in reduced adherence. E.g. “Ticagrelor can cause mild shortness of breath but it does not compromise your physical activities. The shortness of breath usually gets better with time and Ticagrelor is only taken for 12 months”
- Suggest using a multi-compartment medicines system.
- Walk the Participant through the adherence coaching app on mobile phone/computer.
- For non-adherent participants, to schedule a follow-up call in 2-3 days.

4. Provide feedback on BP and HR trend. E.g. “your BP and HR have been stable and in the healthy range. We are prescribing medications that heal your heart after a heart attack but these medications can lower your blood pressure and heart rate, which is why we are monitoring these regularly. This will ensure that you get the maximum healing benefit from the medications without compromising heart rate.”
   a. If the participant has been compliant and is taking the correct dose go to step b.
   b. “In this case, I would need to increase the medications that help with the healing of your heart. Research has shown that these medications can heal the heart more effectively if we get you up to the right dose quickly. Please increase your_ (medication name_dose to _ (new dose). I will call you again on __day at__time. Please continue to check your blood pressure reading from Monday to Sunday, twice a day; morning & evening like before.”
   c. “Before we finish the call, please repeat the names and doses of all medications that you are taking and record these medications in your medication booklet/smartphone app”
   d. Follow up with a phone call and update if there is any action ordered by doctor.

5. Enquire about adopting heart healthy lifestyle (limit time spend to no more than 15 min)

Reinforce the targets where necessary – refer to the “Heart Recovery” Manual

a. “Are you adapting well to your new heart healthy lifestyle?”

b. “Have you been eating well or sleeping well?”

c. “Have you been able to exercise?”

d. “Did you manage to stop smoking?” (If relevant)

e. “Is there anything that you would like to clarify about your new lifestyle?”

f. When exceeded 15 minutes (with no other significant concerns), “Shall I follow up on this matter the next time I call you?”

(Take note and document)

6. Closing

“Thank you for your time and I will call you again on _________.”

Missed Readings

1. Introduce yourself

“Good _______ , I am nurse ______________ from National University Hospital Telehealth team. May I speak to _______________ (participant’s name)?”

2. Tell the person purpose of the call

“We have not been receiving your BP readings”

a. “May I verify with you how you check your blood pressure? Please explain the steps you take your blood pressure.” (If participant verbalizes the correct technique - go to step b. If not, please reinforce correct technique before proceeding to step b.)

b. “Kindly check your blood pressure once more after 20 minutes and I will call later.”

After 20 minutes if reading is NOT received, repeat step 1-2.

After 20 minutes if reading is received, go to step 3.

3. Provide feedback on BP reading.

““Good _______, I am nurse ______________ from National University Hospital Telehealth team calling you about your blood pressure monitoring.”

High Readings

<u>For high readings</u>

"Your blood pressure readings have been high."

a) "Can I check how you have been taking your blood pressure medicine?"

If the participant has been compliant and is taking the correct dose go to step b.

b) "In this case, I would need to increase your medication. Please increase your_(medication name_dose to _(new dose). I will call you again after 1 week. Please continue to check your blood pressure reading from Monday to Sunday, twice a day; morning and evening like before."

c) Follow up with a phone call and update if there is any action ordered by doctor.

Low Readings

<u>For low systolic readings</u>

"Your blood pressure readings have been low."

a. "Can I check how you have been taking your blood pressure medicine?"

If the Participant has been compliant and is taking the correct dose go to step b.

b. "Have you been drinking enough water and eating well?" (Note the Participant's response. If the Participant is not eating or drinking well, advise to hydrate self accordingly but according to daily limits set by doctor if any. If no limits advise 8 glasses of water (200mls per glass), including other orally consumed fluids such as juices and soups. If hydration is not an issue, go to step c.

c. "In this case, I would need to advise some changes to your medications."

Refer to action plan in "*Drug Titration*" & "*Signs & Symptoms"* protocol to advise Participant.

"I will call you again on _______. Please continue to check your blood pressure reading from Monday to Sunday, twice a day as before."

d. Follow up with a phone call and update if there is any action ordered by doctor

4. Closing

"Thank you for your time and I will call you again on _________."

Transition to adherence coaching phase at end of month 2

1. Introduce yourself

“Good _______ , I am nurse ______________ from National University Hospital Telehealth team. May I speak to _______________ (participant’s name)?”

2. Inform of Transition

“Congratulations. With your hard work, we have achieved the right dose of medications that will heal your heart most effectively. You may return the meter and gateway to your respective research coordinator. For the next 4 months, we will now move on to calls every 2 weeks instead of every week. During these 4 months, I will continue to monitor your condition and help you achieve a healthy heart.”

Month 3 to 6

1. Discontinuation of BP/HR monitoring once participant reaches maximal doses of ACE-I/ARB and BB. Repeat step 1-6 under week 1-8 script but omit step 4.

Termination of Telehealth intervention at end of month 6

1. Introduce yourself

“Good _______ , I am nurse ______________ from National University Hospital Telehealth team. May I speak to _______________ (participant’s name)?”

2. “Congratulations. With your hard work, we have completed the 6-month Telehealth program to achieve good heart health. Please remember to go for your heart MRI scan to follow-up on your heart condition and your regular appointments with your cardiologist. Thank you and it has been a pleasure taking care of your heart.”

FAQ

1. Appointment related matters

NUH

“Kindly call the appointment line at 6772 2002 to check your appointment matters. Alternatively, you may also go to www.nuh.com.sg and check under appointment.”

TTSH

“Kindly call the appointment line at 6357 7000 to check your appointment matters. Alternatively, you may also go towww.ttsh.com.sg and check under 'Patient-guide' and click on 'appointments'.”

Figure 10. Telehealth stepped Care (dose titration) algorithm

Telemedicine blood pressure and pulse rate surveillance with drug titration protocol for Post-MI patients

- PI initiates post MI drugs to be titrated.
- Order is keyed into CPSS.
- Ensure patient has adequate dose drug for titration protocol.
- Recommendation is to prescribe daily target (maximum) dose of ACE-I/ARB and β−blocker at discharge for 4 weeks (till 30 day appointment with cardiologist).
- Clinical research coordinator (CRC) gives clear instructions to participant on drug titration protocol and symptoms and signs to be aware of.
- CRC issues Telemetrix+ BP/HR device to patient.

There will be run-in period of <10 days from discharge to first CMR during which participant will be started on twice daily BP and HR self-check (Mon-Sun) after discharge. Adherence to BP/HR measurements will be assessed during this period.

Once 1st CMR acquired, proceed with drug titration protocol.

- Weekly titration X 8 weeks
- Alternate between ACE-I/ARB and BB titration
- Measure BP and HR again in 2 hours if drugs are uptitrated
- Maintain SBP≥110 mm Hg
- Maintain HR≥55 bpm
- Timeline: Reach target dose within 6 weeks(±2 weeks)

Titrate ACE-I (or ARB) dose on discharge and maintain same dose of β–blocker → **Half** of target dose ACE-I (or ARB) → Titrate **β–blocker** → **Half** of target dose **β–blocker** → Alternate till Target dose for both drugs

ACE I	Starting dose	1st Titration	2nd Titration	3rd Titration (if SBP >140 mm Hg)	Target dose	Max dose
Ramipril	2.5 mg OM	5 mg OM	10 mg OM	-	10 mg OM	10 mg OM
Perindopril	2 mg OM	4 mg OM	8 mg OM	16 mg OM	8 mg OD	16 mg OM
Lisinopril	2.5mg OM	5 mg OM	10 mg OM	20 mg OM	10 mg OD	40 mg OM
Enalapril	2.5 mg BD	5 mg BD	10 mg BD	20 mg BD	10 mg BD	20 mg BD

If intolerant of ACE I because of cough

ARB	Starting dose	1st Titration	2nd Titration	3rd Titration	Target dose	Max dose
Losartan	25mg OD	50 mg OM	100 mg OM	-	100 mg OM	100mg OM
Valsartan	40 mg BD	80 mg BD	160 mg BD	-	160 mg BD	160 mg BD
Candesartan	4 mg BD	8 mg BD	16 mg BD	-	16 mg BD	16 mg BD

β–blocker	Starting dose	1st Titration	2nd Titration	3rd Titration	Target Dose	Max dose
Bisoprolol	1.25 mg OM	2.5 mgOM	5 mg OM	10 mg OM	10 mg OM	20 mg OM
Carvedilol	3.125mg BD	6.25 mg BD	12.5 mg BD	25 mg BD	25 mg BD	50 mg BD
Metoprolol	12.5 mg OM	12.5 mg tds	25 mg tds	50 mg tds	50mg tds	50 mg tds

SBP>110 mm Hg and HR ≥55 bpm AND no symptoms of hypotension or bradycardia

NO → Down titrate and maintain highest tolerated dose at which target BP and/or HR is maintained

Target dose achieved?

YES → Maintain Target Dose

NO → Maintain highest tolerated dose at which target BP and/or HR is maintained

Figure 11. Action Plan

(A)

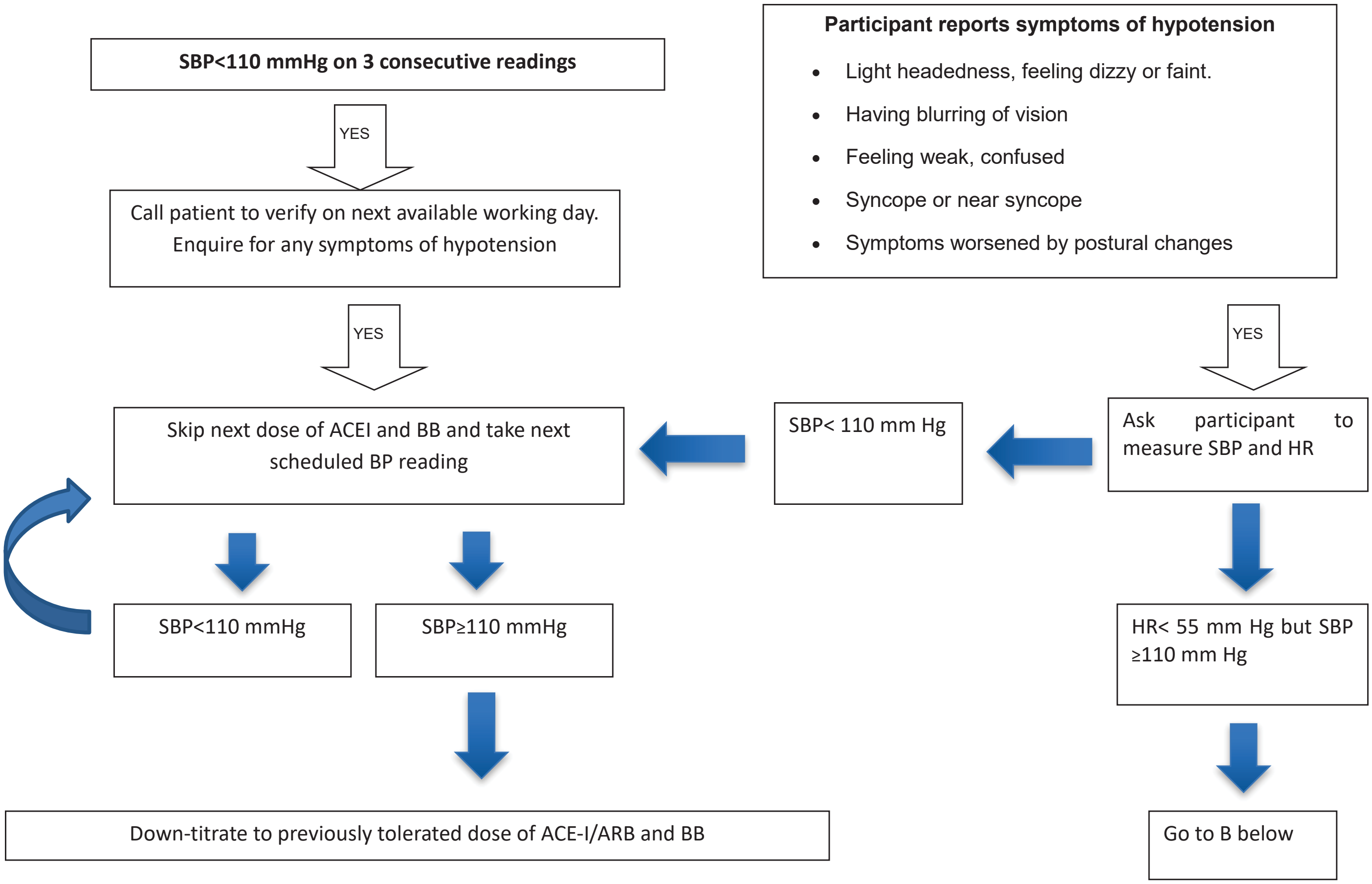

(B)

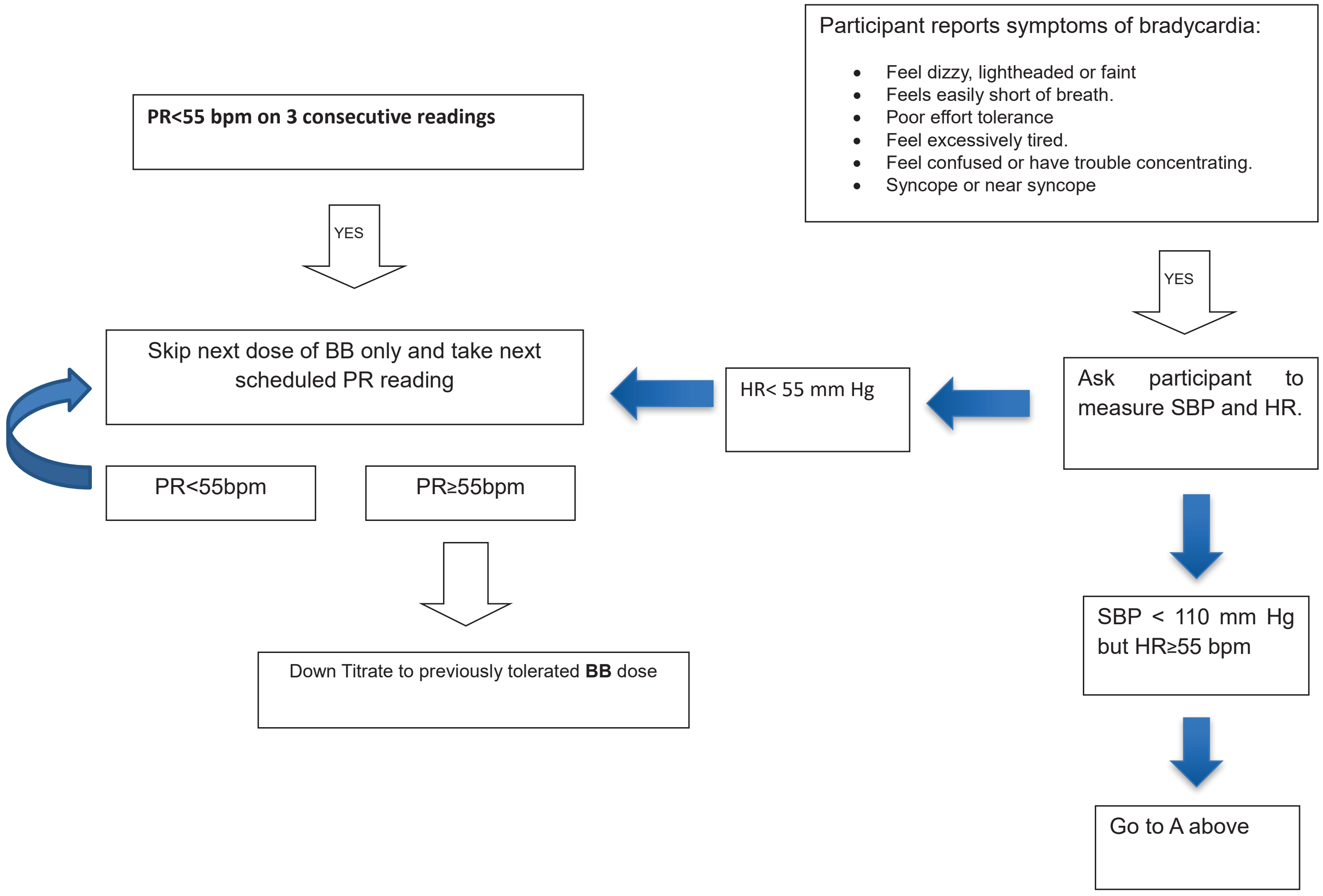
Participant reports symptoms of bradycardia:
Feel dizzy, lightheaded or faint
Feels easily short of breath.
Poor effort tolerance
Feel excessively tired.
Feel confused or have trouble concentrating.
Syncope or near syncope
YES
Ask participant to measure SBP and HR.
HR< 55 mm Hg
SBP < 110 mm Hg but HR≥55 bpm
Go to A above
PR<55 bpm on 3 consecutive readings
YES
Skip next dose of BB only and take next scheduled PR reading
PR<55bpm
PR≥55bpm
Down Titrate to previously tolerated BB dose

Table 1. Symptom management and escalation protocol

**Signs and Symptoms**	**Intervention/Action**
Chest discomfort / pain	• Assess nature of chest discomfort/pain whether is cardiac or non-cardiac origin<br>• Assess associated symptoms<br>• Provide relevant advice* / reassurance to participant<br>• Provide plan of care and inform cardiologist in-charge<br>• Review daily over the next 2 working days or until resolution of symptoms/signs<br>• Intervention/action taken recorded in CDoc<br>• If in severe pain or similar to admitting episode, advice to seek for immediate medical attention
Light headedness / Dizziness / Pre-syncope / Syncope	• Assess associated symptoms + BP, PR<br>• Assess for precipitating causes<br>• Titrate medications if needed<br>• Provide relevant advice* to participant<br>• Provide plan of care and inform cardiologist in-charge<br>• Review closely over next 2 working days or until resolution of symptoms/signs<br>• Intervention/action taken recorded in CDoc
Shortness of breath	• Assess associated symptoms and precipitating causes<br>• If acute heart failure is suspected, contact cardiologist to consider for a clinic review<br>• Otherwise, provide plan of care and inform cardiologist in-charge<br>• Review daily over the next 2 working days or until resolution of symptoms/signs<br>• Intervention/action taken recorded in CDoc
Other signs and symptoms	• Obtain history and review of symptoms<br>• If critical, advise* to seek immediate medical attention<br>• Inform cardiologist in-charge for next course of action<br>• Update participant plan of care<br>• Intervention/action taken recorded in CDoc
* Relevant advice may include:	

- Instructions on medication titrations
- Ensure adequate hydration and avoid sudden movements to reduce postural related lightheadedness / dizziness
- High BP – education on signs & symptoms of stroke eg. Slurring, weakness of limbs, severe headache
- Advice on any issues or deficits with self-care measures identified (such as non-adherence to medication or non-heart healthy lifestyle).
- Call healthcare personnel on telephone number provided during office hours if symptoms worsened or not improved by the following day.
- Immediately go to emergency department in event of sudden/acute deterioration (such as acute shortness of breath, chest pain, s/s of stroke, sustained palpitations or diaphoresis).

7. Post-Index Procedure Management: Follow-up phase

7.1 Clinical and Laboratory follow-up

Follow-up visits will take place at 1month, 6 months and 12 months, timed with the Participant's routine consultations with their cardiologist. The cardiac physician will interview patient for clinical and adverse events, performing a symptoms checklist as well as physical examination. Clinical events recorded will include death, MI, stroke, readmission for recurrent ischemia requiring unplanned revascularization and readmission for heart failure, as well as the primary safety endpoints. Original source documents must be submitted for any clinical events. If the participant is readmitted to a non-study hospital, all possible efforts should be made to obtain original source documents from that hospital.

To ensure that ascertainment of secondary outcomes is blinded to treatment arm assignment, follow-up will be by a web-based questionnaire or mailed questionnaire (both self-administered). Participants are reimbursed for completion of each CMR/PET scan and continue to receive all their standard medical care. Reimbursements are given for CMR scan as this is apart from their usual follow-up care.
If participants are found to have thrombus after enrolment and are prescribed warfarin therapy, INR value and warfarin dosage will be captured in the case report form.

7.2 Study Visit procedures

Enrolment

Participants will be enrolled based on inclusion/exclusion criteria of the study. Baseline medical information such as onset and presentation of chest pain, initial electrocardiogram (ECG), loading dose of antiplatelet therapy, angiographic datas and clinical blood results will be captured. Upon enrolment, P2Y12 antagonist will be changed to Ticagrelor if participant is not already prescribed. Suggested ace-i would be Lisinopril, ramipril, perindopril or enalapril. For ARB – candesartan, valsartan or losartan, and beta-blockers – metoprolol, bisoprolol or carvedilol.

(Blood pressure device will be dispensed to participants randomised toTelehealth arm and will be educated on the use and data transmission of the device before discharge from hospital. Instructions on twice a day measurement (before breakfast and before sleeping) and on daily basis will be advised. Caution: Participants must not share device with other family members).

Visit 1

Participants will be followed up 5-10 days post MI (from date of Index PCI), with window period of +5days for baseline MRI. Participants will be fasted overnight (plain water allowed during fasting) at this visit for both fasting glucose & Insulin under research blood tests. Research blood draw may be done at the time of cannulation during cardiac MRI scan. The exact time of last dose Ticagrelor consumed by the participant, as well as the exact time of blood draw will be documented each time when participant comes for follow-up.

During this visit, participant questionnaires will administered be completed by the participants. Participants are encouraged to increase fluid intake after MRI scan to enhance elimination of contrast agent in the body.

(Telehealth arm: Drug titrations for Ace-i/ARB and betablockers will commence after participants has completed baseline cardiac MRI. Blood pressures and titration will be monitored by Advance Practiced Nurses).

Visit 2 (1-month)

Participants will return to hospital for Cardiologist follow up in clinic for post-MI review. Participant's clinical blood tests and research bloods may be done at the same draw to prevent additional venipuncture. Safety endpoints, Study event checklist, Physical assessment and *BARC bleeding scores will be assessed by the cardiologist reviewing the participant during clinic follow-up. Ticagrelor pill-count will be done by the study coordinator during this visit for drug adherence and accountability. The exact time of last dose Ticagrelor consumed by the participant, as well as the exact time of blood draw will be documented. Participants will complete both participant questionnaire and cost-effective analysis questionnaire during this visit. Research bloods will include Multiplate ADP, NT-proBNP will be measured using Alere POC.

(Telehealth arm: Participants will complete drug titration after 2months (8-weeks) after which they will return the blood pressure device to the study coordinators. A post-telehealth intervention survey will be administered at this time. Telehealth consult will continue bi-weekly for next 4months for medication adherence and coaching by APNs).

Visit 3 (6-month)

During this visit, participants will have completed telehealth intervention program before returning for 6-month cardiac MRI scan. Participants will be fasted overnight (plain water allowed during fasting) at this visit for both fasting glucose & Insulin done under research blood tests. Research blood draw may be done at the time of cannulation during cardiac MRI scan. After completion of MRI scan, participant may be followed up in clinic for 6-month review. A 12-lead ECG will be done before Cardiologist review. During Cardiologist follow up, ECG, Safety endpoints, Study event checklist, Physical assessment and *BARC bleeding scores will be assessed by the physicians reviewing the participants during clinic follow-ups. Ticagrelor pill-counts will be done by the study coordinator during this visit for drug adherence. The exact time of last dose Ticagrelor consumed by the participant, as well as the exact time of blood draw will be documented. Participants will complete both participant questionnaires and cost-effective analysis questionnaires during this visit. Research bloods will include Multiplate ADP, and NT-proBNP will be measured using Alere POC.

Visit 4 (12-month)

This is final Cardiologist follow up in clinic for post-MI review. Participants may have clinical blood tests drawn together with research bloods to prevent additional venipuncture. Safety endpoints, Study event

checklist, Physical assessment and *BARC bleeding scores will be assessed by the physicians reviewing the participants during clinic follow-ups. Final Ticagrelor pill-counts will be done by the study coordinator during this visit for drug adherence. The exact time of last dose Ticagrelor consumed by the participant, as well as the exact time of blood draw will be documented. Participants will complete both participant questionnaires and cost-effective analysis questionnaires during this visit. Decision will be made by the consulting cardiologist whether or not to continue follow-up clinically. Research bloods will include Multiplate ADP, and NT-proBNP will be measured using Alere POC.

*To fill up adverse event forms if BARC bleeding scores is ≥2

Follow-up 5 (18-month) and 6 (24-month)

No face-to-face follow-up is required for this follow up. A Phone call follow-up will be done to determine any outcome events if participants no longer has any appointment visits to the hospital. Participants will complete questionnaires either online or by return-mail.

7.3 Schedule of measurement

Table 2.Study Schedule of IMMACULATE

		V1	V2	V3	V4	F/U 5	F/U 6
Methods of assessment	Enrolment	5-10 days (+5 days)	1 mo (± 2wks)	6mo (± 1 mo)	12mo (± 1 mo)	18 mo (± 2 mo)	24 mo (± 2 mo)
CMR/PET		T, C		T, C			
12-Lead ECG	T, C (inpt)			T, C			
Follow-up visit (Cardiologist)[1]			T, C	T, C	T, C		
Alere POC (NT-pro-BNP)	T, C		T, C	T, C	T, C		
HOMA-IR		T, C		T, C			
Research biomarkers		T, C	T, C	T, C	T, C		
Clinical bld tests[2]	T, C (inpt)		T, C (O/p)	T, C (O/p)	T, C (O/p)		
Questionnaires (self-administered)		T, C	T, C	T, C	T, C	T, C	T, C
Adverse Events Assessment		T, C	T, C	T, C	T, C	T, C	T, C
BARC Bleeding Assessment			T, C	T, C	T, C		

T = Telehealth, C = Control
inpt = data from inpt hospital system

O/p = data from outpatient lab tests

[1]Participants randomized to the TELEHEALTH group will have, in addition to regular visits to the cardiologist, weekly telehealth consultations with the nurse practitioner for 2 months (8weeks), followed by fortnightly consultations for another 4 months.
[2]Fasting glucose, electrolytes, creatinine and lipid monitoring except visit 2.
[3] Ascertainment of outcomes and questionnaires will be self-administered to avoid interviewer bias. These questionnaires will be administered during the 1-week, 1 mo, 6mo, 12 mo, 18 mo and 24 mo follow-up visits. Several options will be made available to participants including web-based and mailed questionnaires (email or regular mail) for follow-up at 18- and 24-months.

8. Methods of Measurement

8.1 Imaging

8.1.1 Cardiac Imaging

Eligible participants will have cardiac magnetic resonance (CMR) imaging scans.

A 1.5 or 3 Tesla magnetic resonance scanner will be used depending on availability at each of the centres.

The MRI protocol consists of:

- Cine sequences
- T2-weighted imaging
- First pass perfusion imaging
- Tagging
- Flow imaging
- Delayed Enhancement imaging
- Pre-contrast T1 MOLLI map (T1 mapping)

- **Cardiac MRI 1st Visit** (5-10days post PCI)
- **Cardiac MRI 2nd Visit** (6-month)

The detailed protocol can be found in the IMMACULATE imaging manual.

8.1.2. PET imaging (MR-PET substudy for NUH participants only)

The preference of glucose over fatty acids (FA) has significant cardioprotective benefit [8], including a reduction in toxic intermediates of incomplete FA metabolism [9] and **increased cardiac efficiency** [10]. However, a reduction in insulin action in cardiac myocytes can accelerate post-MI LV dysfunction [11] because of reduced substrate availability with decline in glucose transport capacity and a rapid decline in mitochondrial FA oxidative capacity. It has been shown that a possible decline in insulin action can be due to cardiac leptin signaling that results in inadequate glucose utilization in the face of cardiac stress after MI [8]. Our project proposes to use PET/MRI technology to develop methodology for assessing myocardial efficiency in post-MI participants. The goal is to develop the methodology for management of therapy for participants after MI. We propose to evaluate cardiac efficiency using MRI to calculate strain

(distance), finite element (FE) modeling to estimate stress (force), and PET to measure O_2 utilization (energy expenditure). *Our hypothesis is that LV efficiency derived from* ***PET (^{11}C-acetate)/MRI*** *has high predictive value for LV remodeling.* The detailed protocol can be found in the IMMACULATE imaging manual.

9. Clinical Events (and definitions)

Death: The primary endpoint includes death from any cause. In addition, the cause of death (cardiovascular vs. non-cardiovascular) will be adjudicated.

MI: The diagnosis of acute myocardial infarction is based on the universal definition of myocardial infarction. Myocardial infarction must be distinct from the index event and is defined by symptoms suggestive of ischemia/infarction, electrocardiographic data, cardiac biomarker, or pathologic evidence of infarction.

Stroke: Stroke is defined as the rapid onset of new, persistent, neurologic deficit lasting at least 24 hours (or resulting in death before 24 hours). Stroke, as detected by the occurrence of a new neurologic deficit, was confirmed by a neurologist and on imaging.

Readmission for recurrent ischaemia requiring unplanned revascularization: defined as any subsequent PCI procedure or CABG surgery, as determined by the participant's physician to be clinically indicated. A staged PCI procedure should not be confused with clinically driven repeat revascularization procedures on initial target lesion(s) or new lesions during or after the initial hospitalization, and should not be counted. Staged PCI procedures usually occur within one month of the initial procedure. A revascularization is considered clinically indicated if angiography at follow-up shows a percent diameter stenosis ≥50% AND if one of the following occurs: (1) a positive history of recurrent angina pectoris; (2) admission for unstable angina, MI or heart failure presumably due to the initial target lesion or new lesions.

Readmission for heart failure: Death due to heart failure, hospital admission, emergency department visit, or 24-hour observation stay with at least 2 signs and/or symptoms of HF among the following:

Shortness of breath/dyspnea on exertion, paroxysmal nocturnal dyspnea, orthopnea, fatigue/reduce exercise tolerance, pulmonary edema, JVD, rales, S3, hepatojugular reflux, altered hemodynamics, peripheral edema or cardiomegaly

AND

Treatment with or increase in dosage if previously prescribed for another cause (i.e. hypertension) of loop diuretics or treatment with IV vasoactive agents, specifically for the symptoms described above.

10. Data Management, Imaging Analysis, Statistical Analysis and Sample Size Calculation

10.1 Data Management

Relevant data will be collected and entered into a computerized database by specialized personnel at the Cardiovascular Research Institute (CVRI), Singapore. All data will be verified with the use of hospital records or telephone interviews, and all adverse clinical events will be adjudicated by an events committee whose members are unaware of participants' treatment assignments. Also, all analyses will be performed

blinded to treatment assignments. Data coordination and site management services will be performed by the CVRI.

10.2 CMR Analysis

An investigator unaware of treatment assignment will do all image analyses. Please refer to the IMMACULATE imaging manual for a detailed image acquisition, storage and analysis protocols.

10.3 Statistical Analysis

The primary analysis will be performed on all randomized participants who complete both baseline and 6-month CMR scans. In addition, an on-treatment analysis will be performed on participants who successfully complete >75% of remote consultations in the telehealth group and all patients in the standard care group who do not have any major protocol deviations.

Predefined major protocol violations/deviations are missing data for the primary efficacy end point, violation of inclusion criteria, and additional protocol violations that will be possibly defined during the blind data review. Missing participants will be censored at the last follow-up visit. Vital status will be specifically searched for all missing participants through linkage with national death registers (e.g. the National Births and Deaths Registry in Singapore).

Baseline characteristics will be tabulated and comparability/differences between the treatment groups will be examined by means of descriptive statistics.

We will compare mean LVESV at 6-month in the two study groups adjusting for baseline LVESV, study site and assigned treatment. Differences in least squares means and corresponding 95% CI will be calculated based on the ANCOVA model. While others have compared change in LVESV (final LVESV – baseline LVESV) between treatment groups, the most statistically efficient method is to compare final LVESV adjusted for baseline covariates [25]. All statistical tests will be two-sided with a significance level of 5%.

The frequency of secondary endpoints will be compared based on χ^2 test or Fisher’s exact test. In addition, rate of event-free survival analysis will be performed by the Kaplan-Meier method with log-rank test group comparison. Potentially important prognostic factors for the main efficacy/safety end point will be explored and used as covariates in the relative risk analyses by means of logistic regression or a Cox model. We will analyze secondary endpoints using a Bonferroni correction for the number of secondary endpoints assessed to adjust for multiple comparisons testing.

10.4 Sample Size Calculations

Sample size calculations demonstrated that 150 participants in each group are required to achieve a power of at least 90% to detect a difference in mean LVESV of 3 ml between study groups at 6 months, with one pre-treatment MRI at baseline and one post-treatment MRI at 6 months, assuming a two- sided significance level of $p<0{\cdot}05$, a common standard deviation of 10 ml within each group and a between-scan correlation of 0.7. To achieve 80% power, we will require 90 participants per group. Patients will be randomized 1:1 to the systems approach (n=150) or usual care (n=150), using randomized blocks of 4

and 6. The sample size calculation was performed in STATA 11.1 (College Station, Tx).

We have targeted an enrolment of 300 participants (maximum up to 340). Even after accounting for a 10% attrition rate (N=30), 300 participants will still give us study power in excess of 90%. This power calculation was done on STATA 11.1.

No sample size determination was performed for the other secondary endpoints as these are considered exploratory and hypothesis generating.

11. Safety Assessment

11.1 Adverse Events (AE)

Information about all adverse events, whether volunteered by the participants, discovered by investigator questioning, or detected through physical examination, laboratory test or other means, will be collected and followed as appropriate. An adverse event is any undesirable sign, symptom or medical condition occurring after initiation of the intervention, even if the event is not considered related to study intervention.

- Medical conditions/diseases present before starting study intervention are only considered adverse events if they worsen after starting study intervention. Abnormal laboratory values or test results constitute adverse events only if they induce clinical signs or symptoms that are considered clinically significant or require therapy, or lead to the discontinuation of the participant from the study. These are recorded on the electronic case report forms (CRF) under the signs, symptoms or diagnosis associated with them.
- As far as possible, each adverse event will also be described by:
    1) its duration (start and end dates),
    2) the severity grade (mild, moderate, severe),
    3) the action(s) taken.

11.2. Serious Adverse Events (SAE)

A serious adverse event is an undesirable sign, symptom or medical condition which:

1. is fatal or life-threatening.
2. required in-patient hospitalization or prolonged hospitalization.*
3. results in persistent or significant disability/incapacity.
4. constitutes a congenital anomaly or a birth defect.
5. is medically significant, in that it may jeopardize the participant and may require medical or surgical intervention to prevent one of the outcomes listed above.

- The investigator must report all SAEs to their institutional review board with the SAE Report Forms provided within 24 hours of learning of its occurrence.

* AEs that resulted in ER stay>24 hours are automatically classified as SAEs.

12. Events Adjudication and Reporting

12.1. Investigator Responsibilities

12.1.1. Adverse events

The investigator will assess all adverse events for the severity, seriousness, and the causal relationship to study procedures. All non-serious adverse events are to be reported in detail and in a timely manner to CVRI, on appropriate AE form and Case Report Form pages, whether or not they are believed to be serious or related to the study procedures. As this is not a registered drug/device trial, the PI and team have decided to capture only the following adverse events: myocardial infarction, stroke, bleeding (BARC II and above) and heart failure.

12.1.2. Serious adverse events (SAE)

- All events meeting the SAE criteria must be reported to the CVRI within 24 hours of becoming aware of the events. To be noted that all endpoint events fall into this category, and must be reported within the above timeframe.

- The Investigator must complete the SAE form and complete Case Report Form for each serious adverse event, whether related or not to study or procedure. The information provided must be sufficient to allow for independent medical assessment of the event. The Investigator should provide any additional follow-up information regarding the event to CVRI as soon as it becomes available. All adverse events should be followed until resolution or stabilization.

- The site IRB/EC must be notified by the Investigators within the timeframe specified by their local SOPs and the applicable regulations.

- Planned hospital admissions and/or planned surgical operations for an illness or disease which existed before the device was deployed or the participant was randomized in a clinical study are not to be considered adverse events. However, baseline conditions which deteriorate during a clinical study may be considered adverse events.

- It should be noted here that all clinical endpoints, including MI, Stroke, unscheduled revascularization and death will require central adjudication and are included here, even though they contribute to trial outcomes. The CVRI will serve as the data coordinating centre. The study investigators will be responsible to provide all applicable and available source documentation to the data coordinating centre in order to allow an independent assessment of these events by the Clinical Events Committee (CEC) members.

- Serious adverse events that do not require expedited reporting need to be reported to AstraZeneca quarterly as individual case reports or as line-listings. When reporting to AstraZeneca, a cover page should accompany the SAE form indicating the following:

  - External Sponsored Research (ESR)
  - The investigator's name and address
  - The trial name/title and AstraZeneca ESR reference number.
  - Investigative site must indicate, either in the SAE report or the cover page, the causality of events in relation to all study medications.

Send SAE report and accompanying cover page by way of fax to AstraZeneca's designated fax line: +46 31 776 37 34

12.2. Sponsor or Designee's Responsibilities

12.2.1. Reporting responsibilities

All SAEs will be reported to the local institutional review Board within 7 calendar days as required by local IRB, and the same report will concurrently be forwarded to AstraZeneca.

12.2.2. Endpoint and SAE Adjudication

- With the exception of all-cause mortality, most endpoints will require clear, prespecified criteria, and centralized review. These endpoints will be captured during Participant interview, supplemented by death certificates; hospital record abstracts and related reports (autopsy, biopsy, diagnostic output). These endpoints will be adjudicated using the same procedure as SAEs.
- From extensive experience, the following approach is proposed. First, all required documents, reports, hospital records will be identified, and copied to the CVRI by clinical staff. Second, the CVRI will check to ensure confidentiality and, if required, have the records centrally abstracted onto standard forms by trained CVRI staff. Central abstraction in large (>30) batches is recommended to reduce variability and secular drift and maintain adequate accuracy and completeness. Third, centrally prepared forms and documents will be circulated to CEC members for assessment. Anonymization is not necessary as CEC members are study team members.

13. Discontinuations

13.1 Participants Inadvertently Enrolled

The criteria for enrollment must be followed explicitly. In the rare case where a participant who does not meet enrollment criteria is inadvertently enrolled, the overall study PI, A/Prof Mark Chan and respective site PI must be contacted. If it is determined after discussion with the PI that, in considering participant safety, it is appropriate to continue the participant will continue on the Telehealth Intervention and be monitored for all visits and testing for the duration of the study. If after discussion with the PI, it is determined that the participant should not be continued in the study, the participant will be excluded from the study. In both instances, a protocol deviation must be submitted to the DSRB.

13.2 Telehealth Intervention

13.2.1 Temporary discontinuation of Telehealth intervention

There may be situations in which the Telehealth intervention may be temporarily discontinued, due to rehospitalization for efficacy events, safety events or elective coronary artery bypass surgery. All rehospitalizations should be informed within the next working day to the site CRC or project lead, Miss Poh Sock Cheng. Unless there are specific reasons for permanent discontinuation, the telehealth

intervention shall resume upon discharge.

13.2.2 Permanent Discontinuation of Telehealth intervention

Very rarely, it may be necessary for a participant to permanently discontinue the Telehealth intervention. Situations requiring permanent discontinuation of the Telehealth intervention may include sudden disability resulting in an inability to comply with Telehealth consultations, leaving Singapore permanently or death. Development of intolerance to either ACE-I/ARB or betablockers should not be reason for discontinuation of the Telehealth intervention, as monitoring and coaching can still continue even with omission of one of these medications. If the participant is unable to comply with Telehealth consultations, every effort should be made to engage a caregiver to take part in the Telehealth consultations.

Participants who permanently discontinue the Telehealth intervention prior to completing the study should have an on-site follow-up visit performed and will remain in the study to be evaluated for all tests, efficacy and safety endpoints. Adverse events, serious and non-serious, will be collected for 30 days after the most recent Telehealth consultation. Thereafter, serious adverse events will not be reported unless the investigator feels the events were related to the protocol procedure. If a participant is unwilling or unable to return for this on-site visit, sites should still attempt to collect as much visit information as possible, including through telephone contact.

Until the Final Visit, all Participants who permanently discontinued the Telehealth Intervention and did not withdraw participation from the study will be followed by telephone visits that will replace all future scheduled visits.

If discontinuation is due to an adverse event, the event is to be followed according to the procedures in Section 8 of this protocol. Some possible reasons that may lead to permanent discontinuation of the Telehealth Intervention include:

1. The participant was inadvertently randomized and in the opinion of the PI, continuation of the telehealth intervention is not advisable.
2. In the opinion of the PI, an adverse event or a significant change in a laboratory value warrants permanent discontinuation of the Telehealth Intervention.
3. Females who become pregnant during the maintenance phase of the study and have to be taken off ACE-I/ARB or betablockers.
4. The participant requests to stop the Telehealth intervention permanently.

13.3. Ticagrelor

13.3.1 Temporary discontinuation of Ticagrelor

There may be situations in which Ticagrelor may be temporarily discontinued, for example, due to bleeding or elective coronary artery bypass surgery. Ticagrelor should be stopped 5 days prior to elective coronary bypass surgery as recommended in the product information sheet.

In event of bleeding, Full attempts should be made to classify the bleeding event according to the BARC classification [18, 26]. As far as possible, Ticagrelor should not be discontinued during episodes of minor

bleeding (BARC type 1 and 2). Episodes of major bleeding (BARC type 3, 4 and 5) should be informed within 24 hours to the site study coordinator and by default, will be categorized as serious adverse events unless proven otherwise. Temporary discontinuation of Ticagrelor, and likely aspirin, is often required in event of a major bleed. As far as possible, Ticagrelor should be restarted, with or without a loading dose, upon resolution of the major bleeding episode. Such cases should be closely discussed with the site PI.

We discourage site investigators from discontinuing Ticagrelor due to dyspnea, as this has been shown to have no adverse consequence on clinical outcomes, nor does dyspnea negate the mortality benefit from treatment with Ticagrelor [27].

13.3.2 Permanent Discontinuation of Ticagrelor

Very rarely, it may be necessary for a participant to permanently discontinue Ticagrelor prior to the 12 months of designated treatment. Situations requiring permanent discontinuation of Ticagrelor include intracranial hemorrhage, symptomatic ventricular pauses and women who become pregnant during the course of the trial. Participants who permanently discontinue the Ticagrelor prior to completing the study should have an on-site follow-up visit performed and will remain in the study to be evaluated for all tests, efficacy and safety endpoints. Adverse events, serious and non-serious, will be collected for 30 days after the most recent dose of Ticagrelor. Thereafter, serious adverse events will not be reported unless the investigator feels the events were related to the protocol procedure. If a participant is unwilling or unable to return for this on-site visit, sites should still attempt to collect as much visit information as possible, including through telephone contact.

Until the Final Visit, all Participants who permanently discontinue Ticagrelor and did not withdraw participation from the study will be followed by telephone visits that will replace all future scheduled visits. If discontinuation is due to an adverse event, the event is to be followed according to the procedures in Section 8 of this protocol.

13.4 Discontinuation from the Trial

At any time during the trial, a participant, either from the Telehealth or control group, may withdraw their participation from the study. At the time of discontinuation from the study, the project lead, Ms Poh Sock Cheng, should be contacted and, if possible, a final follow-up visit will be scheduled and the self-administered questionnaire should be completed. During a 3-month close-out period at the completion of the trial, survival status will be collected within legal and ethical boundaries for all Participants randomized who withdrew participation from the study.

13.5 Participants Lost to Follow-Up

A participant would be considered lost to follow-up if he or she repeatedly fails to return for scheduled visits and is unable to be contacted by the study site. Site personnel are expected to make diligent attempts to contact participants who fail to return for a scheduled visit. Survival status will be collected within legal and ethical boundaries for all Participants randomized, including those who did not receive

the Teleheath Intervention. Vital status will be searched in public sources during a 3-month close-out period. If vital status is known at the study closure visit, the participant will not be considered lost to follow-up.

13.6 Discontinuation of Study Sites

Study site participation may be discontinued if the Domain Specific Review Board of the study site judges it necessary for any reason.

Table 3. Bleeding Academic Research Consortium Classification

Type 0: no bleeding

Type 1: bleeding that is not actionable and does not cause the patient to seek unscheduled performance of studies, hospitalization, or treatment by a healthcare professional; may include episodes leading to self-discontinuation of medical therapy by the patient without consulting a healthcare professional

Type 2: any overt, actionable sign of hemorrhage (eg, more bleeding than would be expected for a clinical circumstance, including bleeding found by imaging alone) that does not fit the criteria for type 3, 4, or 5 but does meet at least one of the following criteria: (1) requiring nonsurgical, medical intervention by a healthcare professional, (2) leading to hospitalization or increased level of care, or (3) prompting evaluation

Type 3

- Type 3a
  - Overt bleeding plus hemoglobin drop of 3 to <5 g/dL* (provided hemoglobin drop is related to bleed)
  - Any transfusion with overt bleeding
- Type 3b
  - Overt bleeding plus hemoglobin drop ≥5 g/dL* (provided hemoglobin drop is related to bleed)
  - Cardiac tamponade
  - Bleeding requiring surgical intervention for control (excluding dental/nasal/skin/hemorrhoid)
  - Bleeding requiring intravenous vasoactive agents
- Type 3c
  - Intracranial hemorrhage (does not include microbleeds or hemorrhagic transformation, does include intraspinal)
  - Subcategories confirmed by autopsy or imaging or lumbar puncture
  - Intraocular bleed compromising vision

Type 4: CABG-related bleeding

- Perioperative intracranial bleeding within 48 h
- Reoperation after closure of sternotomy for the purpose of controlling bleeding
- Transfusion of ≥5 U whole blood or packed red blood cells within a 48-h period†
- Chest tube output $\geq$2L within a 24-h period

Type 5: fatal bleeding

- Type 5a
  - Probable fatal bleeding; no autopsy or imaging confirmation but clinically suspicious
- Type 5b
  - Definite fatal bleeding; overt bleeding or autopsy or imaging confirmation

CABG indicates coronary artery bypass graft. Platelet transfusions should be recorded and reported but are not included in these definitions until further information is obtained about the relationship to outcomes. If a CABG-related bleed is not adjudicated as at least a type 3 severity event, it will be classified as not a bleeding event. If a bleeding event occurs with a clear temporal relationship to CABG (ie, within a 48-h time frame) but does not meet type 4 severity criteria, it will be classified as not a bleeding event.

*Corrected for transfusion (1 U packed red blood cells or 1 U whole blood=1 g/dL hemoglobin).

†Cell saver products are not counted.

Adapted from reference 18.

14. Study operations, medical oversight and research governance

Study operations will be the responsibility of the Cardiovascular Research Institute (CVRI) Singapore and led by the IMMACULATE trial project leader, Ms Poh Sock Cheng. The Telehealth team will be led by Ms Karen Koh. Associate Professor Mark Chan is the overall PI for the study and site PI for NUH while Dr Ho Hee Hwa is the back-up PI for the trial and site PI for TTSH, and Professor Derek Hausenloy for NHCS. Associate Professor Mark Chan, Dr Ho Hee Hwa, Professor Derek Hausenloy and Professor Mark Richards will provide medical oversight of the trial.

The National University Hospital, Singapore is the study sponsor and the study has been approved by the National Healthcare Group Domain Specific Review Board (ethics review board).

References

1. OECD, *Health at a Glance 2011*. OECD Publishing.
2. Burchfield, J.S., M. Xie, and J.A. Hill, *Pathological ventricular remodeling: mechanisms: part 1 of 2.* Circulation, 2013. **128**(4): p. 388-400.
3. Steg, P.G., et al., *ESC Guidelines for the management of acute myocardial infarction in patients presenting with ST-segment elevation.* Eur Heart J, 2012. **33**(20): p. 2569-619.
4. *Executive Summary: Standards of Medical Care in Diabetes—2012.* Diabetes Care, 2012. **35**: p. no. Supplement 1 S4-S10.
5. Arnold, S.V., et al., *Beyond medication prescription as performance measures: optimal secondary prevention medication dosing after acute myocardial infarction.* J Am Coll Cardiol, 2013. **62**(19): p. 1791-801.
6. *National Registry of Disease Office: Acute Myocardial Infarction Report.* 2010.
7. Lim *Using hospital readmission rates to track the quality of care in public hospitals in Singapore*. 2011. **11**, DOI: 10.1186/1472-6963-11-S1-A16.
8. *American Medical Association Telemedicine Policy.* http://www.ama-assn.org/ama/pub/news/news/2014/2014-06-11-policy-coverage-reimbursement-for-telemedicine.page. 2014.
9. Boessen, R., et al., *Improving clinical trial efficiency by biomarker-guided patient selection.* Trials, 2014. **15**: p. 103.
10. Morrow, D.A., et al., *Prognostic value of serial B-type natriuretic peptide testing during follow-up of patients with unstable coronary artery disease.* JAMA, 2005. **294**(22): p. 2866-71.
11. Richards, A.M., et al., *B-type natriuretic peptides and ejection fraction for prognosis after myocardial infarction.* Circulation, 2003. **107**(22): p. 2786-92.
12. Johri, A.M., et al., *Can a teaching intervention reduce interobserver variability in LVEF assessment: a quality control exercise in the echocardiography lab.* JACC Cardiovasc Imaging, 2011. **4**(8): p. 821-9.
13. Srisawasdi, P., et al., *The effect of renal dysfunction on BNP, NT-proBNP, and their ratio.* Am J Clin Pathol, 2010. **133**(1): p. 14-23.
14. Taylor, J., *2012 ESC Guidelines on acute myocardial infarction (STEMI).* Eur Heart J, 2012. **33**(20): p. 2501-2.
15. Amsterdam, E.A., et al., *2014 AHA/ACC guideline for the management of patients with non-ST-elevation acute coronary syndromes: a report of the American College of Cardiology/American Heart Association Task Force on Practice Guidelines.* Circulation, 2014. **130**(25): p. e344-426.
16. Anderson, J.L., et al., *2012 ACCF/AHA focused update incorporated into the ACCF/AHA 2007 guidelines for the management of patients with unstable angina/non-ST-elevation myocardial infarction: a report of the American College of Cardiology Foundation/American Heart Association Task Force on Practice Guidelines.* J Am Coll Cardiol, 2013. **61**(23): p. e179-347.
17. Morisky, D.E., L.W. Green, and D.M. Levine, *Concurrent and predictive validity of a self-reported measure of medication adherence.* Med Care, 1986. **24**(1): p. 67-74.

18. Mehran, R., et al., *Standardized bleeding definitions for cardiovascular clinical trials: a consensus report from the Bleeding Academic Research Consortium.* Circulation, 2011. **123**(23): p. 2736-47.
19. Thygesen, K., et al., *Third universal definition of myocardial infarction.* Eur Heart J, 2012. **33**(20): p. 2551-67.
20. Fertin, M., et al., *Usefulness of serial assessment of B-type natriuretic peptide, troponin I, and C-reactive protein to predict left ventricular remodeling after acute myocardial infarction (from the REVE-2 study).* Am J Cardiol, 2010. **106**(10): p. 1410-6.
21. Wallentin, L., et al., *Ticagrelor versus clopidogrel in patients with acute coronary syndromes.* N Engl J Med, 2009. **361**(11): p. 1045-57.
22. White, H.D., et al., *Left ventricular end-systolic volume as the major determinant of survival after recovery from myocardial infarction.* Circulation, 1987. **76**(1): p. 44-51.
23. Amsterdam, E.A., et al., *2014 AHA/ACC Guideline for the Management of Patients With Non-ST-Elevation Acute Coronary Syndromes: A Report of the American College of Cardiology/American Heart Association Task Force on Practice Guidelines.* J Am Coll Cardiol, 2014.
24. Tamis-Holland, J.E. and P. O'Gara, *Highlights from the 2013 ACCF/AHA guidelines for the management of ST-elevation myocardial infarction and beyond.* Clin Cardiol, 2014. **37**(4): p. 252-9.
25. Frison, L. and S.J. Pocock, *Repeated measures in clinical trials: analysing mean summary statistics and its implications for design. Statistics in Medicine (1992), 11, 1685- 1704*. 1992.
26. Vranckx, P., et al., *Prospective validation of the Bleeding Academic Research Consortium classification in the all-comer PRODIGY trial.* Eur Heart J, 2014. **35**(37): p. 2524-9.
27. Storey, R.F., et al., *Incidence of dyspnea and assessment of cardiac and pulmonary function in patients with stable coronary artery disease receiving Ticagrelor, clopidogrel, or placebo in the ONSET/OFFSET study.* J Am Coll Cardiol, 2010. **56**(3): p. 185-93.

Statistical Analysis Plan

Study Endpoints

1.0 Primary efficacy endpoint

- Difference in LVESV at 6 months as measured by CMR (ventricular remodeling endpoint)

2.0 Secondary efficacy endpoints

1. CMR

- LV remodeling index at 6 months
- Infarct size (grams and percentage of total LV mass) at 6 months

2. Frequency of participants with reduction in NTproBNP <20% from baseline to 6 months (hemodynamic stress endpoint)
3. Difference in NTproBNP level at 6 months
1. BB and ACE-I/ARB dose intensity at 30 days and 6 months (dose intensity scores will be calculated by converting equivalent doses of each BB and ACE-I/ARB to an ordinal scale from 0 to 5)
5. Difference in the incidence of death, MI, stroke and heart failure at 24 months.

3.0 Exploratory secondary endpoints

Other exploratory secondary endpoints include systolic BP (SBP) and diastolic BP (DBP), LDL-C and, among patients with diabetes mellitus, HbA1c

4.0 Primary safety endpoint

Composite of **rehospitalization** due to hypotension, (systolic BP < 90 mm Hg + symptoms and signs of hypoperfusion), bradycardia (HR < 50 WITH symptoms and signs of hypoperfusion), hyperkalaemia (serum potassium >6.0mmol/L with need for cessation of ACE-I/ARB/aldosterone blockers or potassium-lowering treatment) or acute kidney injury (according to the RIFLE classification – serum creatinine increased > 2 times of baseline measurement or glomerular filtration rate decreased >50%).

5.0 Statistical Analysis

The primary analysis will be performed on all randomized participants who complete both baseline and 6-month CMR scans. In addition, an on-treatment analysis will be performed on participants who successfully complete >75% of remote consultations in the telehealth group and all patients in the standard care group who do not have any major protocol deviations.

Predefined major protocol violations/deviations are missing data for the primary efficacy end point, violation of inclusion criteria, and additional protocol violations that will be possibly defined during the blind data review. Missing participants will be censored at the last follow-up visit. Vital status will be specifically searched for all missing participants through linkage with national death registers (e.g. the National Births and Deaths Registry in Singapore).

Baseline characteristics will be tabulated and comparability/differences between the treatment groups will be examined by means of descriptive statistics.

The mean difference in LVESV, LVEF, LV mass index, LVEDV, NT-pro BNP, LDL and HbA1c at 6 months will be compared between the treatment groups using the t-test. Additionally, adjustment for the respective baseline measurements will be made using the Analysis of Covariance (ANCOVA) test. We will compare mean LVESV at 6-month in the two study groups adjusting for baseline LVESV, study site and assigned treatment. Differences in least squares means and corresponding 95% CI will be calculated based on the ANCOVA model. While others have compared change in LVESV (final LVESV – baseline LVESV) between treatment groups, the most statistically efficient method is to compare final LVESV adjusted for baseline covariates. All statistical tests will be two-sided with a significance level of 5%.

The frequency of secondary endpoints will be compared based on χ^2 test or Fisher's exact test. We will analyze secondary endpoints using a Bonferroni correction for the number of secondary endpoints assessed to adjust for multiple comparisons testing. The difference in proportion with respect to the reduction of NT-pro-BNP by at least 20% at 6 months from baseline will be compared based on Fisher's exact test, with adjustment for baseline covariates made via logistic regression. For repeated secondary outcome measures such as BP and dose intensity of ACE-I/ARB and BB, the linear mixed effect model will be implemented to account for the effect of time, adjusting for the respective baseline covariates. All statistical tests will be 2-sided with a significance level of 5% and performed using STATA version 16 (College Station, TX).

6.0 Sample Size Calculations

Sample size calculations demonstrated that 120 participants in each group are required to achieve a power of at least 90% to detect a difference in mean LVESV of 3 ml between study groups at 6 months, with one pre-treatment MRI at baseline and one post-treatment MRI at 6 months, assuming a two- sided significance level of $p<0{\cdot}05$, a common standard deviation of 10 ml within each group and a between-scan correlation of 0.7. To achieve 80% power, we will require 90 participants per group. Patients will be randomized 1:1 to the systems approach (n=150) or usual care (n=150), using randomized blocks of 4 and 6. The sample size calculation was performed in STATA 11.1 (College Station, Tx).

We have targeted an enrolment of 300 participants. Even after accounting for a 10% attrition rate (N=30), 300 participants will still give us study power in excess of 90%. This power calculation was done on STATA 11.1.

No sample size determination was performed for the other secondary endpoints as these are considered exploratory and hypothesis generating.
