# Supplementary material for: Remote Postdischarge Treatment of Patients With Acute Myocardial Infarction by Allied Health Care Practitioners vs Standard Care: The IMMACULATE Randomized Clinical Trial
Source: JAMA Cardiol. 2020 Dec 30;6(7):1–6. doi: 10.1001/jamacardio.2020.6721 (PMC7774042; doi:10.1001/jamacardio.2020.6721)
Supplement: Supplement 2. — eMethods eTable 1. Conversion of Different ACE/ARB and BB to Dose Intensity Scores eTable 2. Adverse Events of Remote Intensive Management versus Standard Care eTable 3. Serious Adverse Events of Remote Intensive Management versus Standard Care eTable 4. Per-participant Cost Analysis over 6 Months Comparing Remote Intensive Management and Standard Care eFigure 1. Telehealth Dashboard for Remote Intensive Management eFigure 2. Forest Plot for Subgroup Analysis of Primary Outcome eReferences [file jamacardiol-e206721-s002.pdf]

Supplemental Online Content

Chan MY, Koh KWL, Poh S-C, et al; IMMACULATE Investigators. Remote postdischarge treatment of patients with acute myocardial infarction by allied health care practitioners vs standard care: the IMMACULATE Randomized Clinical Trial. *JAMA Cardiol*. Published online December 30, 2020. doi:10.1001/jamacardio.2020.6721

eMethods

eTable 1. Conversion of Different ACE/ARB and BB to Dose Intensity Scores

eTable 2. Adverse Events of Remote Intensive Management versus Standard Care

eTable 3. Serious Adverse Events of Remote Intensive Management versus Standard Care

eTable 4. Per-participant Cost Analysis over 6 Months Comparing Remote Intensive Management and Standard Care

eFigure 1. Telehealth Dashboard for Remote Intensive Management

eFigure 2. Forest Plot for Subgroup Analysis of Primary Outcome

eReferences

This supplemental material has been provided by the authors to give readers additional information about their work.

© 2020 Chan et al. *JAMA Cardiology.*

eMethods

Main Inclusion and Exclusion Criteria

The main inclusion criteria were patients aged between 21 and 85 years with clinically diagnosed ST-segment elevation myocardial infarction (STEMI) or non-ST-segment elevation myocardial infarction (NSTEMI) with ischemic chest pain or angina equivalent symptoms and a typical rise or fall of cardiac troponin I/T referenced to the 99th percentile of the local laboratory value. Patients were excluded if they had an NT-pro-BNP <300 pg/ml, significant valvular heart disease, were planned for coronary artery bypass grafting within 6 weeks, had contraindications to cardiovascular magnetic resonance (CMR) imaging or had psychosocial barriers to telemedicine adoption (see **Clinical Study Protocol** in this **Supplement**).

Enrichment with Point-of-Care NT-pro-BNP at Enrolment

After obtaining written informed consent, NT-pro-BNP was measured in whole blood on a point-of-care Alere Triage analyzer (Alere, San Diego, CA). Because elevated NT-pro-BNP in the initial few days after AMI is associated with worse left ventricular remodelling, heart failure and mortality, we only randomized participants if their point-of-care NT-pro-BNP was >300 pg/ml[1,2].

Randomization

Eligible participants were randomized 1:1 to RIM or SC. Random permuted blocks with randomly varying block sizes of 4 and 6 were implemented with randomization stratified by hospital. Ten participants had to be re-randomized because they were included in a hybrid magnetic resonance-positron emission tomography substudy using C_{11}-acetate but quality control for C11-acetate failed for these 10 participants.

Cardiovascular Magnetic Resonance Imaging

Baseline CMR imaging was performed between 5 to 10 days of the index admission date and repeated at 6 months in accordance with a standardized imaging protocol (**Imaging Acquisition and Analysis** Manual in this **Supplement**). All images were acquired on the following CMR scanners: 3T Siemens MAGNETOM Prisma or 3T Siemens Biograph mMR (Siemens Healthineers, Erlangen, Germany) at the National University Heart Centre, 1.5T Siemens MAGNETOM Aera (Siemens Healthineers, Erlangen, Germany) at the National Heart Centre, and 1.5T Phillip Ingenia (Philips Healthcare, Best,

© 2020 Chan et al. *JAMA Cardiology.*

Netherlands) at Tan Tock Seng Hospital. All 6-month CMR imaging was performed using the same CMR scanner as the baseline CMR scan for each participant. CMR cine sequences were analysed automatically using a machine learning pipeline that we have previously validated in several independent datasets[3]. Volumes were derived from the automatic segmentation of the left and right ventricles (U-Net deep learning network) and 10% of the images were manually verified to confirm the accuracy of the segmentations (see **Imaging Acquisition and Analysis Manual** in this **Supplement**).

Intervention

After the baseline CMR scan, participants randomized to RIM were instructed to begin transmitting twice daily blood pressure (BP) and heart rate (HR) measurements immediately using a Bluetooth-enabled portable device (FORA, Taipei, Taiwan or OMRON, Kyoto, Japan). All measurements were pushed to a remote monitoring dashboard (MyHealthSentinel, Singapore, Singapore)[4]. Weekly consultations were conducted via telephone for 2 months by nurse practitioners who remotely uptitrated ACE-I/ARB and BB according to a standardized algorithm (page 29 of **Clinical Study Protocol** in this **Supplement**). The nurse practitioners have a Masters of Nursing degree, have at least 10 years of nursing experience and have at least 3 years of experience running nursing-led physician-supported clinical cardiology services. They had to have demonstrated the required skills sets in providing care and education for participants with AMI in areas such as (1) disease process; (2) treatment modalities; (3) management of cardiovascular risk factors; (4) commonly cardiac drug pharmacology; (5) open communication skills to address psychosocial concerns. For safety reasons, the dashboard was programmed to flag low BP as <110 mm Hg systolic or low HR as <55 beats per minute, which are more conservative thresholds than those used in clinical trials of face-to-face drug titration[5-7]. The titration protocol also prevented nurse practitioners from uptitrating both BB and ACE-I/ARB at a single consultation. The first measurements of serum creatinine and potassium were performed at 30 days post-randomization, unless the nurse practitioners were of the opinion that earlier testing was required.

Each consultation included scripted coaching on smoking cessation, dietary modifications, exercise prescriptions and medication adherence. After 2 months, the frequency of remote consultations was reduced to once every 2 weeks over the next 4 months. The telehealth service was provided as a weekday (Monday-to-Friday), office hours (8 AM-6 PM) service and all participants were given instructions to seek alternative care for emergencies outside of these operating hours.

© 2020 Chan et al. *JAMA Cardiology.*

Standard Care

Participants randomized to SC received regular face-to-face consultations with their cardiologists who would perform medication uptitration according to each hospital's clinical pathway. The frequency of these consultations were determined by the participant's primary cardiologist. SC participants did not receive any telemonitoring equipment or teleconsultations.

Follow-up

Participants were scheduled to have follow-up visits with a trial coordinator and trial physician at 30 days, 6 months and 1 year. The responsibilities of the trial coordinator included recording the dose of all medications prescribed, administering a quality of life questionnaire and blood draws for NT-pro-BNP, fasting lipids, glycated haemoglobin A1C (HbA1c), creatinine and potassium. The responsibilities of the trial physician included interviewing the participant for adverse events, performing a symptoms checklist and physical examination. As far as possible, we avoided having the participants' primary cardiologist perform trial physician responsibilities. The trial physician was permitted to uptitrate or downtitrate a participant's ACE-I/ARB and BB if they were of the opinion that the current dose was inappropriately low or high but had to first seek permission from the participant's primary cardiologist.

Outcomes

The primary safety endpoint was a composite of hospitalization due to 1. hypotension (SBP <90 mm Hg with symptoms and signs of hypoperfusion), 2. bradycardia (HR <50 with symptoms and signs of hypoperfusion), 3. hyperkalaemia (serum potassium >6.0 mmol/L with need for cessation of ACE-I/ARB/aldosterone blockers or potassium-lowering treatment) or 4. acute kidney injury (serum creatinine increased >2 times of baseline measurement or glomerular filtration rate decreased >50% of baseline[10]). All participants were monitored for any additional adverse events (AEs) and serious adverse events (SAEs) that did not fulfil criteria of the primary safety endpoint.

Dose intensity of BB and ACE-I/ARB between RIM and SC were compared at 30 days and 6 months. A dose intensity score of BB and ACE-I/ARB was calculated by converting equivalent doses of each BB and ACE-I/ARB to an ordinal scale from 0 to 5[8,9]. For example, a total daily dose of either ramipril 2.5 mg, lisinopril 2.5 mg or enalapril 5 mg was assigned a dose intensity of 2 while the next uptitration

© 2020 Chan et al. *JAMA Cardiology.*

to a total daily dose of either ramipril 5 mg, lisinopril 5 mg or enalapril 10 mg was assigned a dose intensity of 3 (**eTable 1** in this **Supplement**).

The primary efficacy endpoint was indexed LVESV at 6 months. The secondary efficacy endpoints included the following: LVEF and indexed LV mass at 6 months, reduction in NT-pro-BNP <20% from baseline to 6 months, difference in NT-pro-BNP concentration at 6 months and

Cost Evaluation

We counted both scheduled and unscheduled face-to-face visits. Although no face-to-face visits were scheduled for RIM participants, the RIM protocol required nurse practitioners to escalate unexpected symptoms or events (e.g. new onset chest pain or bleeding) to the participant's primary cardiologist, which could then lead to an unscheduled face-to-face visit. The average cost per face-to-face visit with the participant's primary cardiologist across the 3 hospitals was 88 SGD (65 USD). Teleconsultation costs included both manpower costs (nurse practitioner) and technology costs (equipment costs, server and network maintenance costs). The cost per teleconsultation was 45 SGD (33 USD).

Statistical Analysis

Randomized participants who successfully completed both baseline and 6-month CMR scans and had interpretable images were included in the primary efficacy endpoint analysis. Analysis of the primary safety endpoint and AE/SAE included randomized participants who had any follow up data; this was to ensure that adverse events were captured to the fullest extent even among participants with even nominal exposure to RIM. Participants who were lost-to-follow-up were censored at the last follow-up visit. The mean difference in LVESV, LVEF, LV mass index and NT-pro BNP at 6 months was compared between the treatment groups using the t-test and adjustment for the respective baseline measurements was made using the Analysis of Covariance (ANCOVA) test[11]. The difference in proportion with respect to the reduction of NT-pro-BNP by at least 20% at 6 months from baseline was compared based on Fisher's exact test, with adjustment for baseline covariates made via logistic regression. For repeated secondary outcome measures such dose intensity of ACE-I/ARB and BB, the linear mixed effect model was implemented to account for the effect of time, adjusting for the respective baseline covariates. All statistical tests were 2-sided with a significance level of 5% and performed using STATA version 16 (College Station, TX).

© 2020 Chan et al. *JAMA Cardiology.*

Power Calculations

Sample size calculations demonstrated that 120 participants in each group were required to achieve a power of at least 90% to detect a difference in mean indexed LVESV of 3 ml between study groups at 6 months, with one pre-treatment MRI at baseline and one post-treatment MRI at 6 months, assuming a common standard deviation of 10 ml for both groups, an intra-subject correlation of 0.7 for the repeated scans and a 2-sided significance level of 5%[12]. The complete **Statistical Analysis Plan** can be found in **Supplement 1**.

Reporting of Ethnicity

Ethnicity was self-reported with the following options made available to the participants: Chinese, Malay, Indian and Others. Ethnicity was captured because of the higher incidence of AMI among Malay and Indian ethnicities[13]. The IMMACULATE investigators sought to have fair representation of Malay and Indian participants in the trial.

© 2020 Chan et al. *JAMA Cardiology.*

eTable 1. Conversion of Different ACE/ARB and BB to Dose Intensity Scores

Dose intensity	Very low intensity	Low intensity	Moderate intensity	High intensity	Maximum intensity	Target dose
**ACE-I I**	**ACEI/ARB 1**	**ACE-I/ARB 2**	**ACE-I/ARB 3**	**ACE-I/ARB 4**	**ACE-I /ARB 5**	
Ramipril	2.5 mg	2.5 mg	5 mg	10 mg	10 mg	10 mg
Perindopril	2 mg	2 mg	4 mg	8 mg	16 mg	8 mg
Lisinopril	2.5mg	2.5mg	5 mg	10 mg OM	20 mg	10 mg
Enalapril	2.5 mg	5 mg	10 mg	20 mg	40 mg	20 mg
Captopril	25 mg	50 mg	75 mg	150 mg	150 mg	150 mg
**ARB**	**ACEI/ARB 1**	**ACEI/ARB 2**	**ACEI/ARB 3**	**ACE-I/ARB 4**	**ACE-I/ARB 5**	
Losartan	25mg	25mg	50 mg	100 mg	100 mg	100 mg
Valsartan	40 mg	80 mg	160 mg	320 mg	320 mg	320 mg
Candesartan	4 mg	8 mg	16 mg	32 mg	32 mg	32 mg
Telmisartan	20 mg	40 mg	80 mg	160 mg	160 mg	160 mg
**Beta-blocker**	**BB 1**	**BB 2**	**BB3**	**BB 4**	**BB 5**	
Bisoprolol	1.25 mg	1.25 mg	2.5 mg	5 mg	10 mg	5 mg
Carvedilol	3.125 mg	6.25 mg	12.5 mg	25 mg	50 mg	25 mg
Metoprolol	12.5 mg	37.5 mg	75 mg	150 mg	150 mg	150 mg
Atenolol	12.5 mg	12.5 mg	25 mg	50 mg	100 mg	50 mg

Abbreviations: ACE, angiotensin converting enzyme inhibitors; ARB, angiotensin receptor blockers; BB, beta-blockers.

Comparable doses of different angiotensin converting enzyme inhibitors and angiotensin receptor blockers were converted to dose intensity scores of ACE/ARB 1-5. Comparable doses of beta blockers were converted to dose intensity scores of BB 1-5.

© 2020 Chan et al. *JAMA Cardiology.*

eTable 2. Adverse Events of Remote Intensive Management versus Standard Care

Adverse Events				
**Adverse Events (AE)**	**Remote intensive management[a] (n=136)**	**Standard Care[a] (n=139)**	**Total Events**	**Total Participants**
**Bleeding (BARC≤II)**	3	8	11	9
**Chest discomfort**	4	5	9	8
**Cough**	4	2	6	6
**Giddiness**	2	2	4	4
**Dyspnoea**	1	0	1	1
**Transient weakness**	1	0	1	1
**Ureteric colic**	1	0	1	1
**Urinary tract infection**	0	1	1	1
**Other minor ailments (e.g. headaches, myalgia, URTI, skin conditions, sprains)**	7	4	11	11
**Total Events[b]**	23	22	45	-
**Total Participants**	23	19	-	42

Abbreviations: BARC, Bleeding Academic Research Consortium; URTI, upper respiratory tract infection

[a] All randomized participants with any follow-up data available.

[b] Recurrent events in same participant treated as new event if previous event resolved and new event occurred >30 days after previous event.

© 2020 Chan et al. *JAMA Cardiology.*

eTable 3. Serious Adverse Events of Remote Intensive Management versus Standard Care

Serious Adverse Events				
**Serious Adverse Events (SAE)**	**Remote intensive management[a] (n=136)**	**Standard Care[a] (n=139)**	**Total Events**	**Total Participants**
**Heart Failure**	7	6	13	10
**Angina/Atypical chest pain[b]**	4	3	7	7
**LV thrombus**	2	4	6	6
**AKI[c]**	1	2	3	3
**Bleeding (BARC≥III)**	0	3	3	2
**Stroke/TIA**	3	0	3	2
**Hypotension[c]**	1	1	2	2
**Hyperkalemia[c]**	1	0	1	1
**Bradycardia[c]**	0	1	1	1
**Acute Pericarditis**	1	0	1	1
**Alcohol intoxication**	0	1	1	1
**Gastritis[b]**	1	0	1	1
**Hodgkin's Lymphoma**	0	1	1	1
**Abnormal ECG[b]**	0	1	1	1
**Palpitations[b]**	0	1	1	1
**Total Events[d]**	21	24	45	-
**Total Participants**	19	21	-	40

Abbreviations: LV, Left Ventricular; AKI, Acute Kidney Injury; BARC, Bleeding Academic Research Consortium; TIA, transient ischemic attack; ECG, electrocardiogram

[a] All randomized participants with any follow-up data available.

[b] AEs that resulted in hospitalization or ER stay>24 hours are automatically classified as SAEs

[c] Excludes primary safety endpoint events. Note that episodes of hypotension, bradycardia, AKI and hyperkalemia not resulting in hospitalization are classified as SAEs but not counted as primary safety events.

[d] Recurrent events in same participant treated as new event if previous event resolved and new event occurred >30 days after previous event.

© 2020 Chan et al. *JAMA Cardiology.*

eTable 4. Per-participant Cost Analysis over 6 Months Comparing Remote Intensive Management and Standard Care

	**Face-to-face clinic visits per participant over 6 months[a]**	**Cost per face-to-face clinic visit[b]**	**Teleconsults per participant over 6 months**	**Cost per teleconsult[c]**	**Total cost per participant over 6 months**
**RIM**	0.67	65 USD	17.8	33 USD	631 USD
**SC**	2.70	65 USD	0	33 USD	176 USD

Abbreviations: RIM, Remote Intensive Management; SC, Standard Care; USD, United States Dollars.

[a] We counted both scheduled and unscheduled face-to-face visits. Although no face-to-face visits were scheduled for RIM participants, the RIM protocol required nurse practitioners to escalate unexpected symptoms or events (e.g. new onset chest pain or bleeding) to the participant's primary cardiologist, which could then lead to an unscheduled face-to-face visit.

[b] Cost per face-to-face visit with the participant's primary cardiologist

[c] Teleconsult costs included both manpower costs (nurse practitioner) and technology costs (equipment costs, server and network maintenance costs). The nurse practitioners have a Masters of Nursing degree, have at least 10 years of nursing experience and have at least 3 years of experience running nursing-led physician-supported clinical cardiology services. They have demonstrated the required skills sets in providing care and education for participants with AMI in areas such as (1) disease process; (2) treatment modalities; (3) management of cardiovascular risk factors; (4) commonly cardiac drug pharmacology; (5) open communication skills to address psychosocial concerns.

© 2020 Chan et al. *JAMA Cardiology.*

eFigure 1. Telehealth Dashboard for Remote Intensive Management

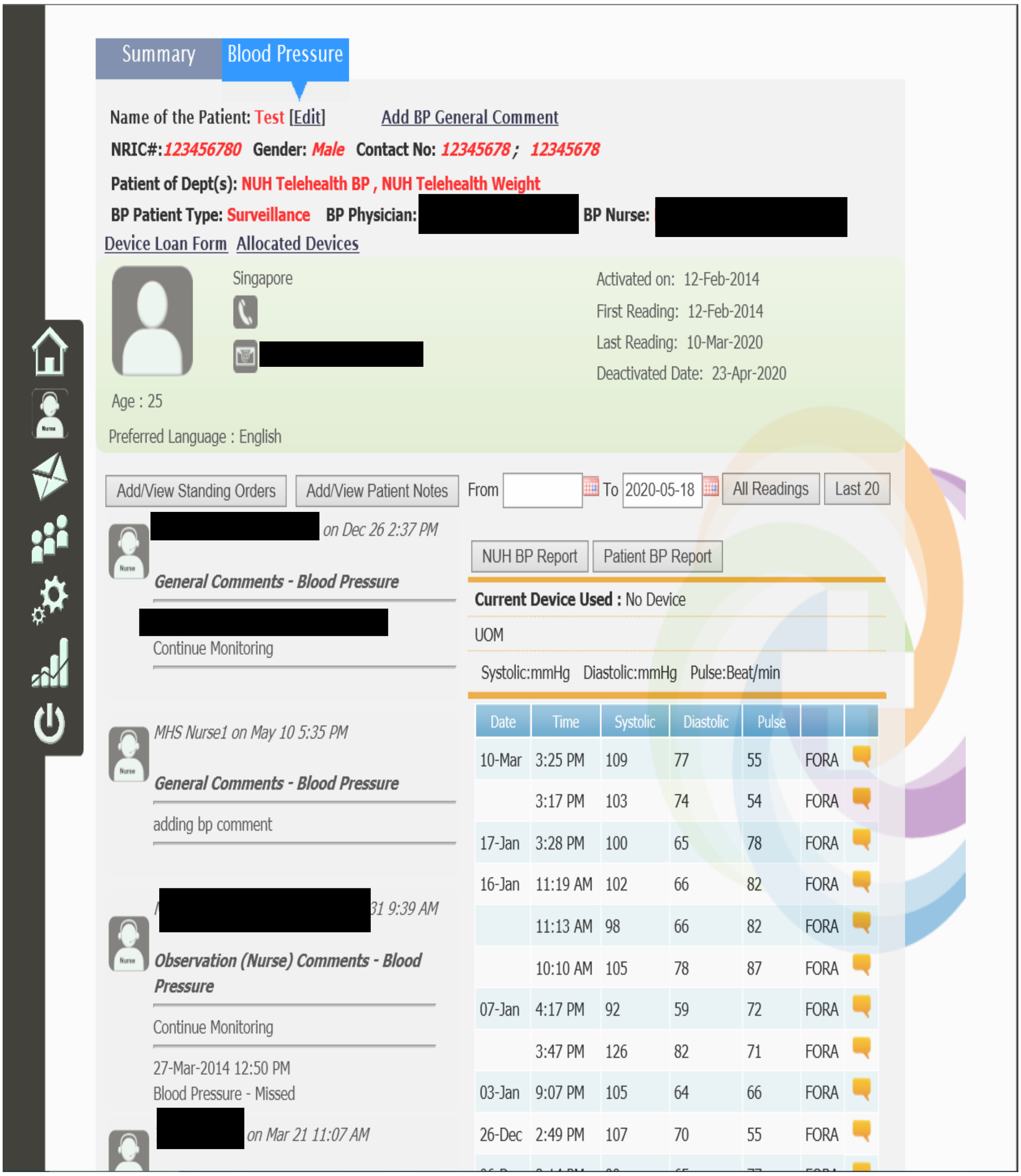

© 2020 Chan et al. *JAMA Cardiology.*

eFigure 2. Forest Plot for Subgroup Analysis of Primary Outcome[a]

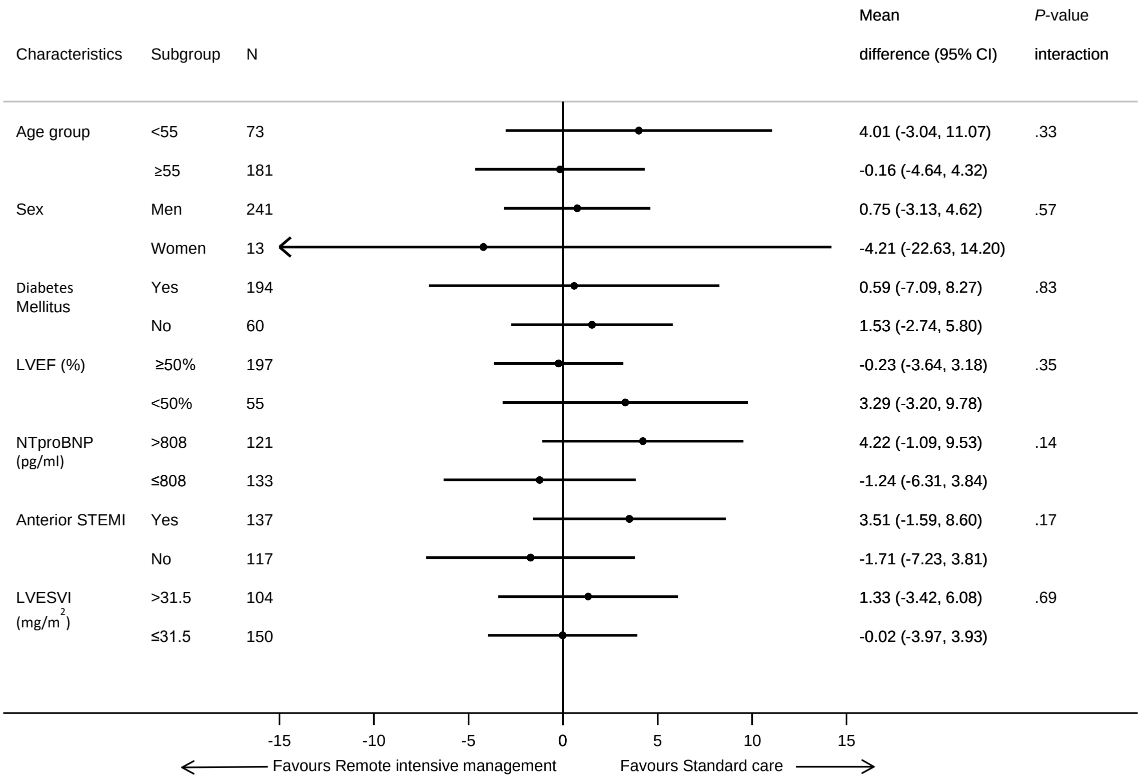

Abbreviation: LVESVI, indexed left ventricular systolic volume; LVEF, left ventricular ejection fraction; NT-pro-BNP, N-Terminal B-type Natriuretic Peptide; CI, confidence interval

[a] Mean difference in LVESVI in ml/m^2 at 6 months between remote intensive management (*n* = 130) and standard care (*n* = 124)

© 2020 Chan et al. *JAMA Cardiology.*

eReferences

1. Richards AM, Nicholls MG, Espiner EA, et al. B-type natriuretic peptides and ejection fraction for prognosis after myocardial infarction. *Circulation.* 2003;107(22):2786-2792.
2. Chan MY, Neely ML, Roe MT, et al. Temporal Biomarker Profiling Reveals Longitudinal Changes in Risk of Death or Myocardial Infarction in Non-ST-Segment Elevation Acute Coronary Syndrome. *Clin Chem.* 2017;63(7):1214-1226.
3. Hakim Fadil JJT, Derek Hausenloy, Hee-Hwa Ho, Prabath Joseph, Adrian Fatt-Hoe Low, A. Mark Richards, Mark Y. Chan and Stephanie Marchesseau. A Deep Learning Pipeline for Automatic Analysis of Multi-Scan Cardiac MRI. *Journal of Cardiovascular Magnetic Resonance.* 2020 (accepted for publication).
4. https://www.myhealthsentinel.com/products-solutions/telemetrix/. Accessed.
5. AIRE Investigators. Effect of ramipril myocardial infarction with clinical evidence of heart failure. *The Lancet.* 1993;342:821-828.
6. Swedberg K, Held P, Kjekshus J, Rasmussen K, Ryden L, Wedel H. Effects of the early administration of enalapril on mortality in patients with acute myocardial infarction. Results of the Cooperative New Scandinavian Enalapril Survival Study II (CONSENSUS II). *N Engl J Med.* 1992;327(10):678-684.
7. TRACE Investigators. A clinical trial of the angiotensin-converting-enzyme inhibitor trandolapril in patients with left ventricular dysfunction after myocardial infarction. *New England Journal of Medicine.* 1995;333:1670-1676.
8. https://www.medscape.org/viewarticle/569559_2. Accessed on 23 Aug 2020.
9. https://www2.gov.bc.ca/assets/gov/health/about-bc-s-health-care-system/bc-guidelines/heart_failure_appendix_b.pdf. Accessed on 23 Aug 2020.
10. Van Biesen W, Vanholder R, Lameire N. Defining acute renal failure: RIFLE and beyond. *Clin J Am Soc Nephrol.* 2006;1(6):1314-1319.
11. Frison L, Pocock SJ. Repeated measures in clinical trials: analysis using mean summary statistics and its implications for design. *Statistics in medicine.* 1992;11(13):1685-1704.
12. Suinesiaputra A, Bluemke DA, Cowan BR, et al. Quantification of LV function and mass by cardiovascular magnetic resonance: multi-center variability and consensus contours. *J Cardiovasc Magn Reson.* 2015;17:63.

© 2020 Chan et al. *JAMA Cardiology.*

13. Chan MY, Shah BR, Gao F, et al. Recalibration of the Global Registry of Acute Coronary Events risk score in a multiethnic Asian population. *Am Heart J.* 2011;162(2):291-299.

© 2020 Chan et al. *JAMA Cardiology.*
