# Supplementary material for: Remote Postdischarge Treatment of Patients With Acute Myocardial Infarction by Allied Health Care Practitioners vs Standard Care: The IMMACULATE Randomized Clinical Trial
Source: JAMA Cardiol. 2020 Dec 30;6(7):1–6. doi: 10.1001/jamacardio.2020.6721 (PMC7774042; doi:10.1001/jamacardio.2020.6721)
Supplement: Supplement 3. — Image acquisition and analysis [file jamacardiol-e206721-s003.pdf]

IMMACULATE MRI Imaging Acquisition and Analysis Manual

1.1 MRI Image Acquisition Protocol (Cardiac Imaging Parameters)

The minimum standards (requirements) for the MRI acquisition are as follows:

Details of compliance to the proposed protocol to be detailed in the Site Evaluation Form SEF.

Requirements for all scans and sequences:

- **Proper Cardiac Shimming**
- **Ensure same number of slices and parameters as previous scans of same Subject**

Plane	Sequence	Name extension	Parameters	Take Note
3 plane	Localizer		ECG trigger and capture cycle for diastolic gating<br>ISO table mode	- Ensure that heart is in the isocenter of image. Otherwise, re-center and repeat
Dark blood Short axis	Dark blood haste<br><br>*(Black Blood SSFSE on GE)*		8 mm slices, 20% gap , straight axials<br>Multiple breath-holds, ECG triggered, **capture cycle**	- Clear above aortic arch to apex<br>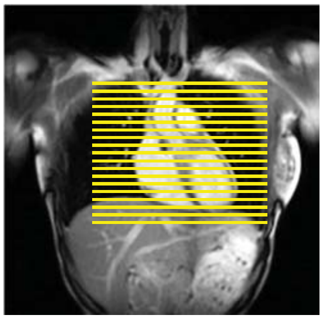 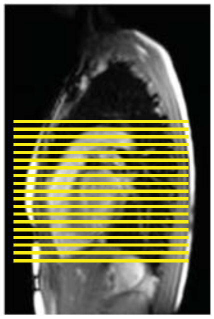
2 chamber	Cines	-2ch	8mm slices	

	(Fiesta Cine on GE)		Iso mode<br>Shim box<br>ECG triggered	
5 slice localizer			**capture cycle**	
4 and 3 chamber	Cines	-4ch, 3ch respectively	8mm slices<br>Iso mode<br>Shim box<br>ECG triggered	
Left ventricular outflow tract (LVOT)	Cines	-3ch cor		
Pre-contrast T1 Map	MOLLI	-sax	~13 x 8 mm slices each<br>Multiple breath-holds	- Clear above ventricles and apex<br>- Ensure same slices are scanned for ventricular short axis an MOLLI<br>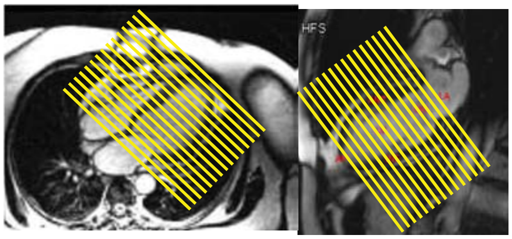
Ventricular Short axis	Cines			
Grid Tagging	FLASH 2D	-mid<br>-base	3 x 8mm slices,<br>Breathhold sequence	- Place 5 equidistant slices with first slice through the valves and the last through the apex

		-apex<br>respectively		- Delete the first and last slice to get 3 relatively reproducible slices
Perfusion test run	TurboFLASH<br>*(FGRE-Time Course or FGRE-Echo Train<br>(IR Prep.) on GE)*	-test	3 x 8mm slices - apex, mid, base<br>FOV:40 cm X 40<br>Matrix: 128 X 128<br>measurements: 50	- Place 5 equidistant slices with first slice through the valves and the last through the apex<br>- Delete the first and last slice to get 3 relatively reproducible slices<br>- Check for artefacts (aliasing etc)<br>- Ensure absence of artefacts before appending
First pass perfusion	TurboFLASH		3 x 8mm slices - apex, mid, base<br>FOV:40 cm X 40<br>Matrix: 128 X 128<br>measurements: 50	- Append from above, change no. of measurements to 50<br>- Inject 5mls of Gadovist @ 5mls/s on the 10th measurement
Injection	Inject remaining calculated dose (0.2 mmol/kg) @ 3mls/s followed by at least 30 ml saline flush after dynamic sequence. Run TI scout after 10 mins.			
Flow studies	FLASH 2D	-AO flow	Venc: ~250	- ensure no aliasing is present otherwise adjust VENC accordingly and edit in sequence name the change in new VENC value
TI scout	IR-prepared cine TrueFISP<br>*(Cine IR on GE)*		Single slice breath-hold<br>**capture cycle**	- Mid-ventricular short axis, where the myocardium is thickest
Phase sensitive	Psir<br>*(Phase sensitive MDE on GE)*	-2ch<br>-4ch<br>-3ch	FOV:35 x 35cm<br>8mm slices @ 20% gap<br>Matrix: 256 x 256	- Optimal TI time should be the TT of last image before appearance of indian ink artefact in TI scout<br>- Ensure that image position corresponds with

		-sax<br>respectively	**capture cycle**<br>In-plane resolution, ~1.4-1.8 mm	respective pre-contrast image<br>- Equal number of slices for sax as ventricular sax<br>- Acquisition duration per R-R interval below 200 ms, but should be less in the setting of tachycardia to avoid image blurring.

Important Reminder: Please ensure proper shimming is done for all scans. A repeat scan will be requested if the above acquisition parameters are not met.

Demonstration of planes

2 Chamber

**Use: Dark blood SAX,**	**Resultant image**
Scroll through, bisect slice through mitral valve and apex,<br>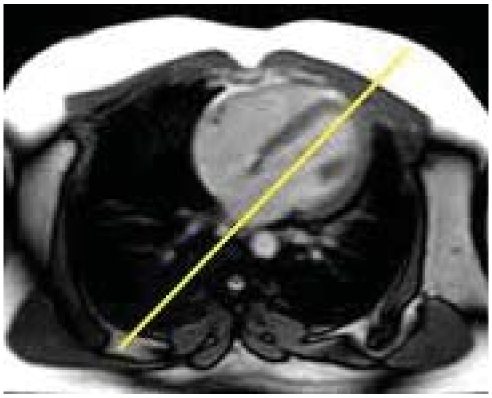	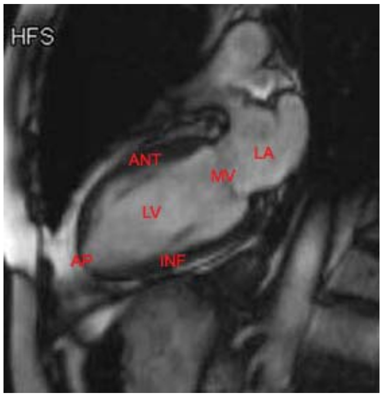

5 slice localizer

**Use: 2 chamber**
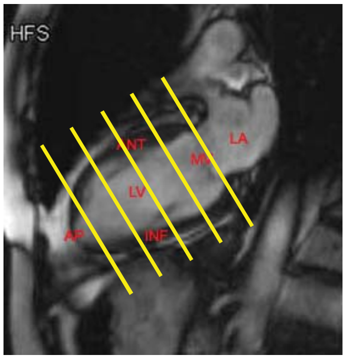

4 chamber

Use: 5 slice localiser	Use: 2 chamber	Resultant image
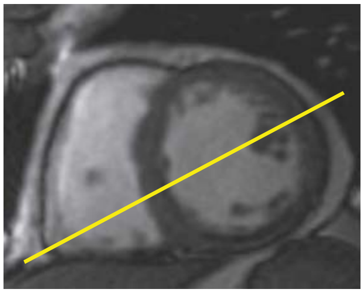	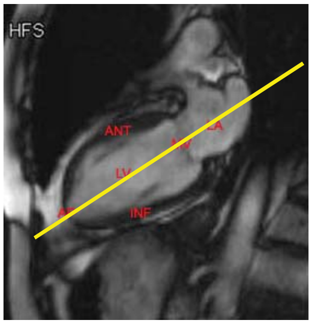	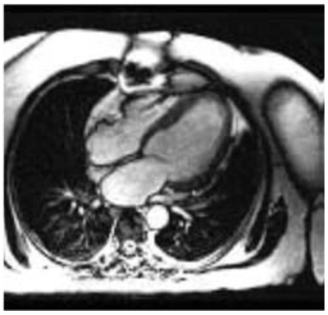

3 chamber

Use: 5 slice localiser	Use: 4 chamber	Resultant image
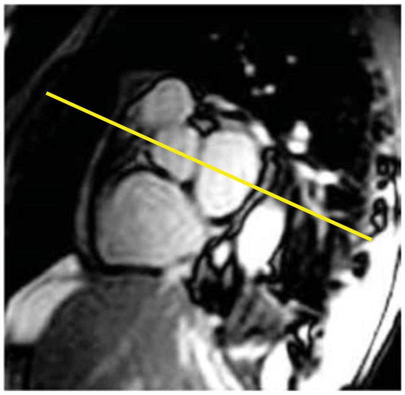	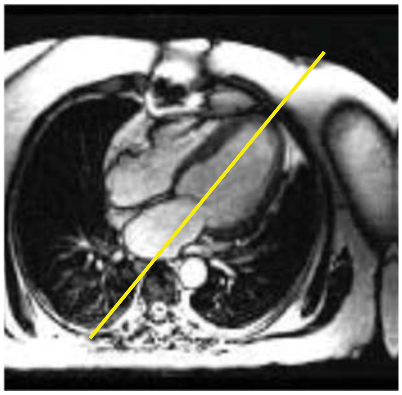	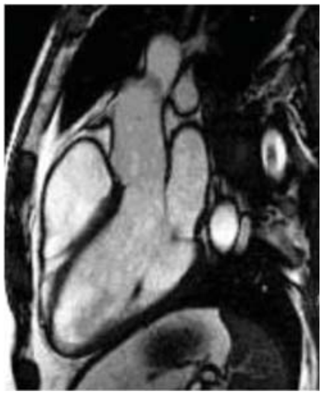

3 chamber coronal

Use: 3 chamber	Resultant image
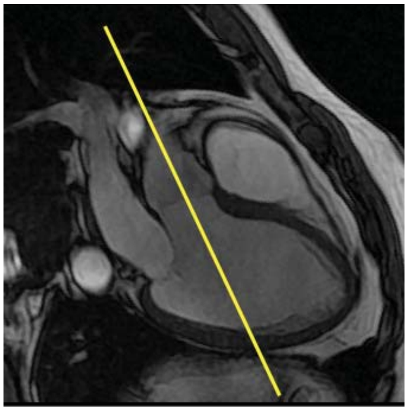	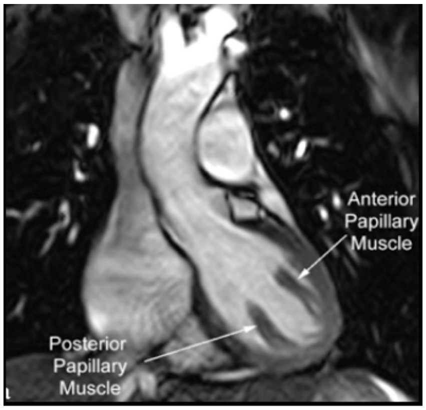

Cardiac Short Axis

Use: 2chamber	Use:4 chamber	Resultant image planes
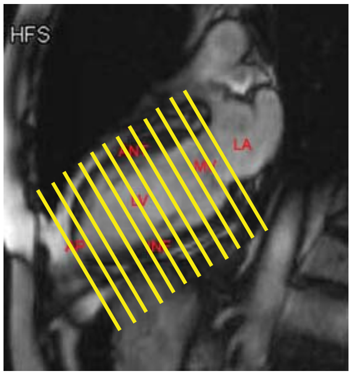	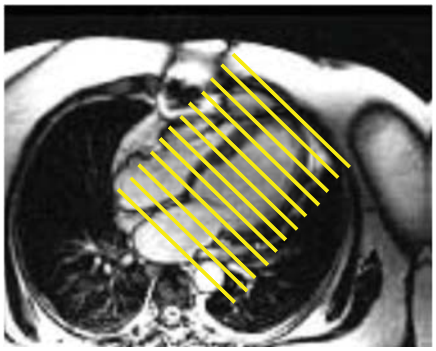	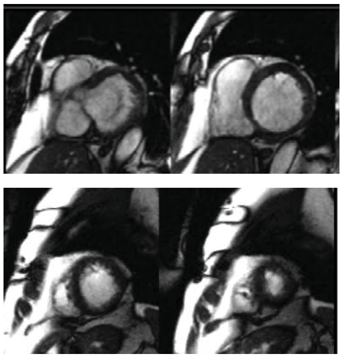

Dynamic & tagging

Use: 4 ch	Use:2 ch
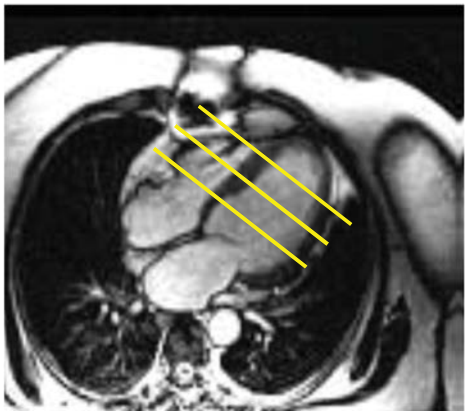	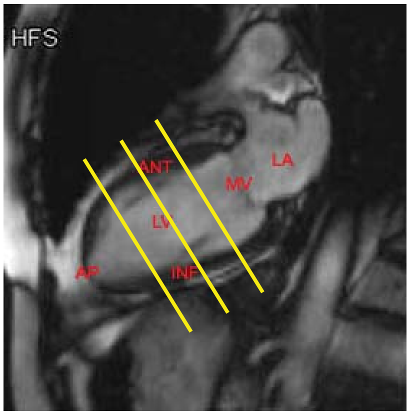
- Place 5 equidistant slices with first slice through the valves and the last through the apex<br>- Delete the first and last slice to get 3 relatively reproducible slices	

AO Flow

Use: 3 Chamber	Use: 3 Chamber Coronal	Resultant image
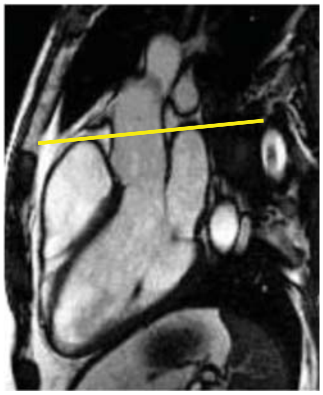	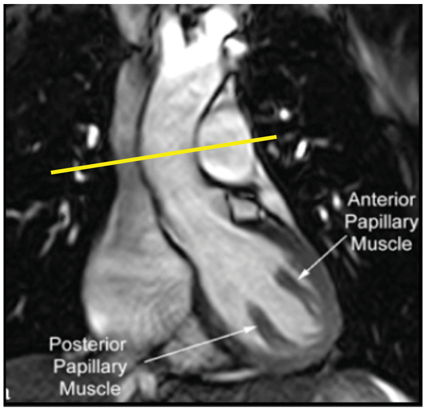 	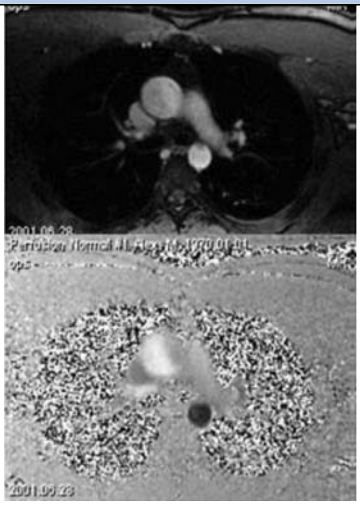

1.2.DATA ANONYMISATION

CIRC will strictly maintain subject confidentiality at all times. Subject name, address, and other personal identifiers will be obscured but without losing the traceability of the data to the study subject identifiers. **Subject name, subject address, subject initials, institution name, medical record number and any other personal information identifying the subject MUST be removed before submission to CIRC.** The technologist must replace the personal information removed with a unique identifier (e.g., 2 letter country code, 3- digit site and 3- digit subject number assigned as part of the clinical study) for subject verification at CIRC.

Personal identifiers should be removed from both the images and the DICOM header file before submission to CIRC. If changing the header information, the technologist must replace the personal information removed with a unique identifier (e.g., 2 letter country code, 3- digit site and 2- digit subject number assigned as part of the clinical study) for subject verification at CIRC. Exact procedures for accessing header information will vary depending on the scanner or system. It is crucial to avoid maskingany of the imaging parameters that may be embedded in the digital files.

Important: Film will NOT be accepted, digital only.

1.3. IMAGE SUBMISSION

General

The acquired images are submitted along with the corresponding documentation to CIRC for Image QC and subsequent processing. The following steps outline the process for submitting data to CIRC for this trial.

The required method of transfer of imaging data from site to CIRC is digital DICOM data on DVD via courier. NOTE: Film will not be accepted.

Before sending the imaging data to CIRC:

1. Fill out the appropriate pre-printed labels on the storage media received.
2. **Complete a single Data Transmittal Form (DTF) for each subject, time point, and imaging modality.** Only one subject should be listed on each DTF. Each DTF must be filled out completely—do not leave any of the DTF fields blank. If the requested information is unknown or not applicable, indicate as such by recording “UNK” or “N/A”.
3. If an error is made while completing the DTF, **do not erase or obliterate the error in any way** (e.g., using correction fluid). Draw a single line through the error and clearly print the correction as close as possible to error, then initial and date the correction.

4. Forward the appropriate DTF along with the corresponding imaging data to CIRC.
5. Retain the site copy of the DTF in the Imaging Binder provided by CIRC in the section marked "Completed DTFs."
6. Complete the sender information and sign the courier waybill provided for this trial. Attach the waybill to a mailer containing the imaging data and DTF(s). Retain the sender's copy of the waybill with the other clinical trial records.

****Note: Each Investigator, along with the Study Coordinator and the designated Radiologist/Technologist, must determine the site-specific procedures regarding the location and method of storing all study-related documentation and supplies (in Radiology area vs. Investigator office/clinic). It is strongly recommended that this is agreed upon prior to the commencement of any imaging evaluations on study subjects.**

Data Transmittal Form (DTF)

For shipping data via courier, the DTF is a non-carbon required (NCR) form used to identify image data and provide specific subject and imaging information. The information is critical for optimal image processing. **One DTF must be completed and forwarded with every imaging study or modality submitted to CIRC.**

The comments section on the DTF is provided to document any imaging-specific details that may provide additional information for CIRC.

Labels

All imaging data submitted to CIRC must contain a completed study-specific label. The label must be attached to the digital media. Discrepancies will result in a query from CIRC to the site requiring resolution before the images can be processed at CIRC.

When completing the labels, care must be taken to ensure that the data entered on the label is consistent with data entered on all corresponding forms (DTFs, CRFs).

Courier Air Waybills

All image data must be sent to CIRC the specific overnight courier selected for this study.

Pre-printed courier air waybills have been provided for the submission of image data to CIRC via the specific overnight courier selected for this trial. It is recommended that a copy of the air waybills be kept in the Imaging Binder for tracking purposes.

Digital Image

All digital images should be stored in duplicate on two separate media in an **uncompressed** or

lossless compressed DICOM format. One will serve as the **source media** to be stored at the site, and one will serve as the identical copy to be submitted to CIRC according to the instructions in section 5.1. See section 5.2 for detailed storage regulations.

Imaging data submitted to CIRC will be retained for the duration of the study.

1.4. IMAGE SCREENING AND STORAGE

Source Data Storage Regulations

The **source** data will always remain at the site and will serve as the original clinical data or source documentation for the IMMACULATE trial (in accordance with ICH/GCP for record retention for research in human subjects).

The research records and primary trial documentation (electronic source data) must be retained 15 years in order to protect the rights of the subjects enrolled in clinical trials. Following closure of the study, the Principal Investigator must maintain all site study records in a safe and secure location. The records must be maintained to allow easy and timely retrieval, when needed (e.g., audit or inspection), and, whenever feasible, to allow any subsequent review of data in conjunction with assessment of the facility, supporting systems, and staff. Where permitted by local laws/regulations or institutional policy, some or all of these records can be maintained in a format other than hard copy (e.g., microfiche, scanned, electronic); however, caution needs to be exercised before such action is taken. The investigator must assure that all reproductions are legible, are a true and accurate copy of the original, and meet accessibility and retrieval standards—including re-generating a hard copy if required. Furthermore, the investigator must ensure there is an acceptable back-up of these reproductions with an acceptable QC process in place for making these reproductions.

CIRC will ensure 5 years storage of the source data in its electronic format and of the documentation but will destroy the DVDs. Additional storage must be negotiated.

The documentation that should be retained for the imaging component of this clinical study includes:

- **Data Transmittal Queries (DTFs):** All copies of DTFs that were completed and sent to CIRC.
- **Data Clarification Forms (DCFs):** A photocopy of any DCFs that were completed and returned to CIRC.
- **Quality Notification Forms (QNFs):** All copies of QNFs as received from CIRC.
- **Correspondence:** Copies of any correspondence received from or sent to CIRC throughout the course of the trial.

Incidental Finding on Images

All scan images at CIRC will be screened for gross abnormality by a radiologist on a regular basis. Any incidental abnormalities will be reported to the respective study Physician.

1.5. QUALITY CONTROL

During the course of the study, there may be instances where CIRC will need to communicate to the sites in reference to the imaging data that is submitted. There are two types of queries that CIRC may employ to communicate to the site: Data Clarification Queries and Quality Notification Queries.

Data Clarification Queries

The query could request information, such as a clarification of a scan date, or a request for additional data (e.g., when the images are not acquired according to the study-specific Imaging Guidelines).The query will request specific action to correct or clarify the outstanding issue. **These are for clarification and require a response by the clinical site**. The issues indicated on the query must be addressed immediately. A description of the resolution of the problem/issue is to be completed by the site, and each query must be responded to. The site is required to print a copy of the query and provide a response to CIRC along with the requested information/data. The printed copy of the query must be retained by the site in the Imaging Binder in the section marked — Completed Data Clarification Queries.

Quality Notification Queries

CIRC will issue a Quality Notification Query to the attention of the clinical site in the event minor issues are noted with submitted imaging data. This query does not require a response, but will serve as a reminder of the protocol guidelines and suggest corrective action for a particular aspect of the imaging procedure the next time that subject is imaged. These queries are sent to ensure that sites are made aware of quality issues and that study data is of the highest quality possible. **These are for informational purposes and do not require a response by the clinical site**. The individual at the site who is responsible for maintaining the study correspondence needs to retain a copy of the query for their records in the Imaging Binder section marked “Quality Notification Queries‖ “.

Below is a table of general query reasons and actions that can be taken to eliminate these queries that will require a response. **Please note that these are not inclusive of queries that may be issued for the trial; additional reasons may arise that result in a query being sent.**

Table 1

Query Reason/Topic	Comments	Site Actions to avoid Query or comment on returned Query
DTF incomplete or not received	DTF identifies the subject, time point, and possibly imaging parameters for the data being submitted	Check shipment package prior to sending; review DTF
DTF and data discrepancies	Information on DTF does not match imaging data (e.g., subject initials, date of scan)	Site review should compare DTF and imaging data prior to submission if possible
Data not received	Data either missing on digital media was not included with DTF	Confirm data are stored on digital media prior to shipment
Incomplete data set	Imaging protocol anatomical regions are not submitted or are missing	If not performed, needs to be documented on DCF or comment section of DTF
Technically inadequate imaging – repeat scan	Based on the imaging parameters for the clinical trial, exceptional quality may be required	If unable to improve, document this on DCF or in the comment section of DTF
Follow-up time point does not match screening	Consistency is critical for clinical trials to perform evaluations	Refer to the screening scan for consistency; if not possible, document on DCF or in the comment section of DTF
Missing time point	Tracker received from client indicates a time point was performed, but CIRC has not received the image	Ensure that as subjects are scanned, the data is immediately forwarded to CIRC

2.1 PET imaging (hybrid MR-PET substudy for NUHC subjects only)

The preference of glucose over FA has significant cardioprotective benefit [8], including a reduction in toxic intermediates of incomplete FA metabolism [9] and **increased cardiac efficiency** [10]. However, a reduction in insulin action in cardiac myocytes can accelerate post-MI LV dysfunction [11] because of reduced substrate availability with decline in glucose transport capacity and the rapid decline in mitochondrial FA oxidative capacity. It has been shown that a possible decline in insulin action can be due to cardiac leptin signaling that results in inadequate glucose utilization in the face of cardiac stress after MI [8]. Our project proposes to use PET/MRI technology to develop methodology for assessing myocardial efficiency in post-MI patients. The goal is to develop the methodology for management of therapy for patients after MI. We propose to evaluate cardiac efficiency using MRI to calculate strain (distance), finite element (FE) modeling to estimate stress (force), and PET to measure O_2 utilization (energy expenditure). *Our hypothesis is that LV efficiency derived from* ***PET(^{11}C-acetate)/MRI*** *has high predictive value for LV remodeling.*

Cardiac tissue efficiency (Fig. 1) is measured as the ratio of cardiac tissue work to measurements of myocardial oxygen consumption (MVO_2) using ^{11}C-acetate kinetics as a surrogate for energy input [13,14,15]. Strain is determined from tagged or DENSE MRI data [16]. This measurement and estimates of stress are used to calculate myocardial external minute work (MEMW) for a *region of tissue* in the left ventricle (LV) wall using the expression:

$$MEMW = (HR/\gamma)\int_{ED}^{ES} T d\varepsilon \quad (\mathrm{N \cdot m \times g^{-1} \times min^{-1}}),$$

where T is the stress tensor, ε is the strain tensor, *ES* is end-systole, *ED* is end-diastole, *HR* is heart rate, and γ is the specific mass of the myocardium (1055 kg/m^3). From dynamic PET data, blood and myocardial time-activity curves (TACs) are used with a 1-compartment kinetic model to estimate the rate k_2(min^{-1}) at which ^{11}C-acetate is converted to $^{11}CO_2$. Values for MVO_2 (μmole x g^{-1} x min^{-1}) are determined using a relationship between k_2 and MVO_2 (MVO_2=135(k_2)-96 ml/100gm/min) [17]. The myocardial oxygen consumption is converted to an energy equivalent assuming 1 ml oxygen = 21 joules.

We propose to perform simultaneous PET and MRI imaging and to use nonlinear tissue mechanical models [18, 19] to estimate cardiac work over the cardiac cycle for individual tissue regions of the LV. This will provide *regional maps* of efficiency (~ 5 mm resolution) rather than global estimates and using our previous FE mechanical models of the border zone [18] will allow determination of efficiency heterogeneity as a function of severity of tissue damage in the peri-infarct zone.

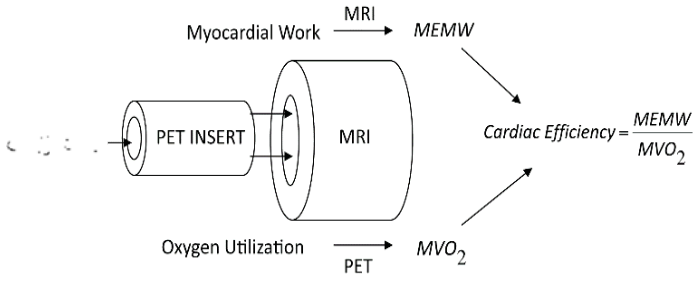

Figure 1 – A strategy to study changes in myocardial efficiency in post-MI studies in humans using hybrid PET/MRI. MEMW is determined using MRI and nonlinear FE models of stress. Oxygen utilization is measured using PET imaging of ^{11}C-acetate. This provides a measure of cardiac efficiency that can be correlated to the metabolism of other energetic substrates such as glucose and fatty acids.

The goal of the proposed project is to use PET/MRI in patients to simultaneously calculate cardiac oxygen utilization using ^{11}C-acetate and cardiac work (ratio=cardiac efficiency) using DENSE MRI [29-41] to study the change in cardiac efficiency with remodeling after MI.

All patient imaging will be performed at the A*STAR-NUS Clinical Imaging Research Centre (CIRC) using the existing combined PET/MR scanner (Siemens mMR). A PET/MRI study will be obtained at baseline at 10-14 days. The study will require an hour's scan of 8 mCi of ^{11}C-acetate and a DENSE MRI study. Each patient will undergo one scan lasting one hour using the Siemens hybrid PET/MRI scanner at CIRC during the 10-14 day visit. Note that 8 mCi of ^{11}C-acetate equates to 296 MBq/scan X 0.0049 mSv/MBq = 1.45 mSv/scan [47]. A 10% error margin will take this to a maximum of 1.6 mSv/scan. Because CMR will be performed at both 10-14 days and 6 months, we will be able to assess the ability of global and regional LV cardiac efficiency measured at baseline to predict LV ventricular remodeling at 6 months.

3.1 Cardiac MR Analysis Protocol (Analysis of CMR Cine Sequences)

The end-diastolic image correspond to the image with the largest LV blood pool while the end-systolic image should be chosen as the image with the smallest blood pool. For their identification, the full image stack has to be evaluated and one phase has to be identified as end-diastole for all the short axis locations and one phase as end-systole for all short axis locations. Deviations may occur and extra care should be taken in the setting of LV dyssynchrony or severe mitral regurgitation. Aortic valve closure defines end-systole. Automatic contour delineation algorithms must be checked for appropriateness by the reader.

a) To compute the LV volumes, contours of endocardial and epicardial borders at end-diastole and end-systole will be segmented according to the following guidelines:

- *Epicardial borders:* should be drawn on the middle of the chemical shift artefact line (when present).
- *Papillary muscles:*are myocardial tissue and thus ideally should be included with the myocardium. As not all evaluation tools allow for their inclusion without manual drawing of contours, they are however often included in the volume in clinical practice, which is acceptable. Reference ranges that use the same approach should be used and the inclusion or exclusion of papillary muscles should be mentioned in the report.
- *Outflow tract:* The LV outflow tract is included as part of the LV blood volume. When aortic valve cusps are identified on the basal slice(s) the contour is drawn to include the outflow tract to the level of the aortic valve cusps.
- *Basal descent:* As a result of systolic motion of the mitral valve toward the apex (basal descent) care must be taken with the one or two most basal slices. A slice that contains blood volume at end-diastole may include only left atrium (LA) without LV blood volume at end-systole. The LA can be identified when less than 50% of the blood volume is surrounded by myocardium and the blood volume cavity is seen to be expanding during systole. Some software packages automatically adjust for systolic atrioventricular ring descent using cross-referencing from long- axis locations.

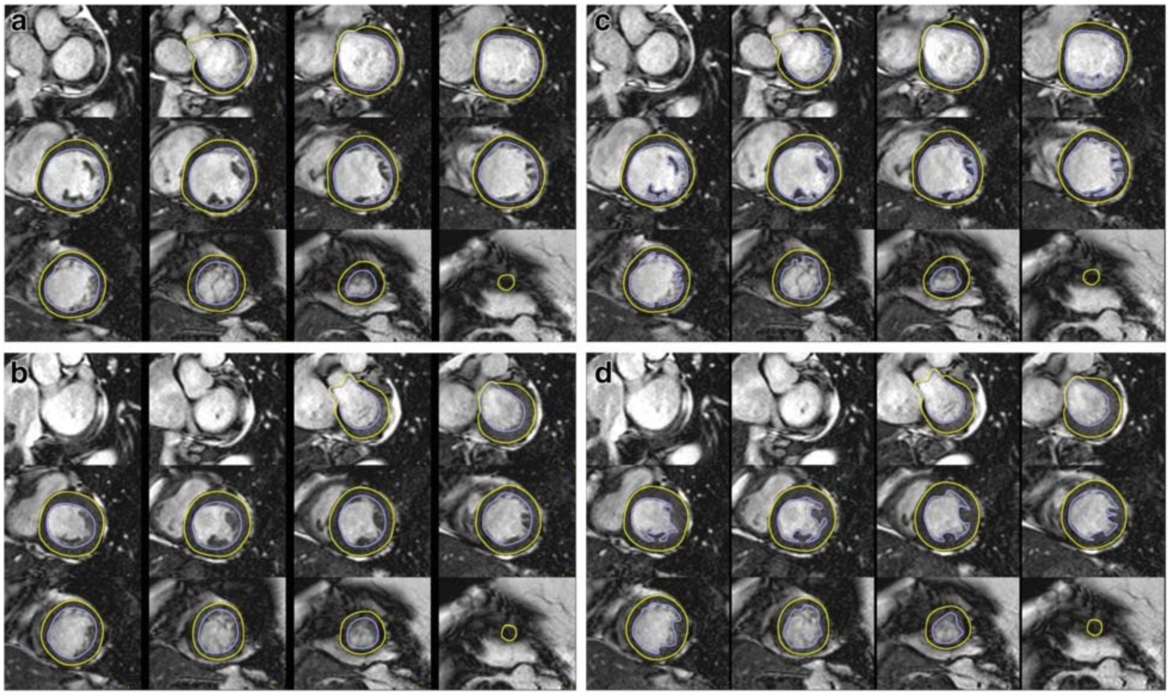

Figure 2: Left ventricular chamber segmentation.*In blue the endocardial contours, in yellow the epicardial contours at (a) diastole and (b) systole in a stack of short axis slices that*

cover the whole ventricle. a) and b) Illustrates the approach with inclusion of the papillary muscles as part of the LV volume. c) and d) Shows the approach with exclusion of the papillary muscles from the LV volume. (Image from [1]).

Volumes at end-diastole and end-systole can then be computed from the endocardial contours using the Simpson's algorithm, and the ejection fraction calculated as:

$$EF = \frac{(EDV - ESV)}{EDV} * 100$$

b) The LV mass is then calculated as the difference between the total epicardial volume (sum of epicardial cross- sectional areas multiplied by the sum of the slice thickness and interslice gap) minus the total endocardial volume (sum of endocardial cross- sectional areas multiplied by the sum of the slice thickness and interslice gap), which is then multiplied by the specific density of myocardium (1.05 g/ml).

3.2 Analysis of the myocardial perfusion imaging (Analysis of perfusion defect)

Perfusion defect can be detected from the first pass perfusion images. Criteria for an inducible perfusion defect are (see Figure 3):

- Occurs first when contrast arrives in LV myocardium
- Persists beyond peak myocardial enhancement and for several RR intervals (usually >4)
- Is more than one pixel wide
- Is usually most prominent in the subendocardial portion of the myocardium
- Often manifests as a transmural gradient across the wall thickness of the segment involved densest in the endocardium and gradually becoming less dense towards the epicardium
- Over time, defect regresses towards the subendocardium
- Is present at stress but not at rest
- Conforms to the distribution territory of one or more coronary arteries

Following the SCMR guidelines, a visual analysis is sufficient and the outputs are the interpretation of the location and extent of inducible perfusion defects using AHA segment model (on a bull's eye) with an estimate number of segments involved, a comment on transmurality of perfusion defect and an indication of the extent of perfusion defect relative to scar on LGE.

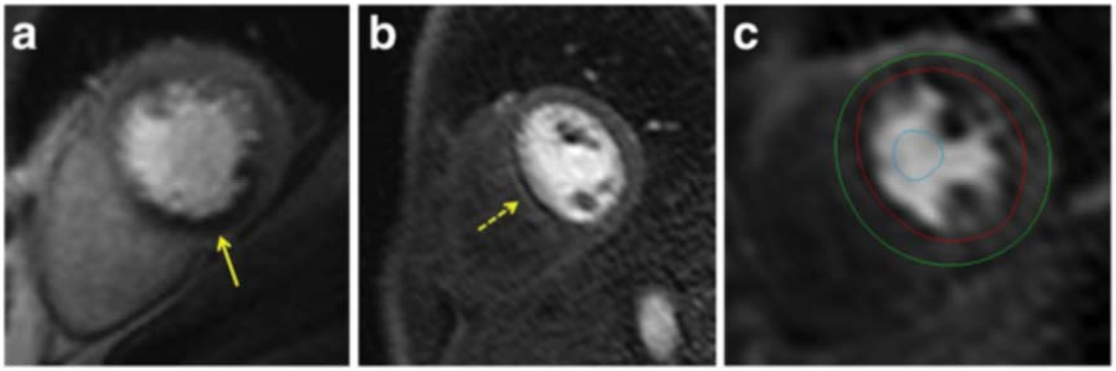

Figure 3: Perfusion imaging.a) Perfusion defect in the inferior segments (yellow arrow). Note defect is predominantly subendocardial, has a physiologically credible distribution (right coronary artery territory) and is more than one pixel wide. b) Dark banding artifact (yellow arrow). Note defect is very dark, occurs already before contrast reaches the myocardium, is seen in the phase encoding direction (right-left in this case), and is approximately one pixel wide. c) Positioning of endocardial (red) and epicardial (green) contours and a ROI in the LV blood pool (blue) for semiquantitative or quantitative analysis of perfusion data. (Image from [1])

However, an objective description of signal intensity change can be performed and several methods have been described for this purpose. This type of quantitative studies are still not validated and no method is recommended by SCMR.

3.3 Analysis of the Delayed Enhancement sequences (Quantification of the Infarct size)

Visual assessment of LGE images is usually sufficient according to the SCMR. The presence of LGE is defined as a high signal intensity that may be as bright as the blood pool. An assessment of the pattern of LGE should be made (Coronary artery disease CAD type or non-CAD type) as well as the interpretation of the location and extent of inducible perfusion defects using AHA segment model (on a bull's eyes) with an estimate average transmural extent within each segment.

However, quantitative analysis can be performed to determine the infarct size expressed in grams and as percentage of total LV mass. The hyperenhanced myocardium is delineated on the LGE image as shown on Figure 4.

Several method exist to delineate the LGE extend as described in the literature including: manual planimetry, the "n"-SD technique and the full width half maximum (FWHM) technique. The SCMR refrains from making a dedicated statement regarding the optimal method.

In each case, total LGE area is given by the summation of these regions for all slices. Multiplication of total LGE area with slice thickness plus interslice gap as well as specific gravity of myocardium provides the approximate LGE weight or infarct weight. We will divide this weight by the total LV mass to compute the ratio to normal myocardium.

3.4 Analysis of the T2-weighted sequences (Quantification of the AAR size)

T2-weighted MRI is performed in the cardiac short-axis direction using a dark-blood T2-weighted inversion-recovery fast-spin echo sequence.

The myocardial edema in the acute phase of myocardial infarction will be visualized in the T2-weighted image as a bright signal, which can distinguish between chronic myocardial infarction and acute myocardial infarction.

In order to assess the area-at-risk, defined as a hyperintense area on T2-weighted images, the signal intensity (SI) in the normal (remote) myocardium will be determined in each slice by tracing a region of interest (ROI) of at least 10 pixels within the visually normal myocardium.
To avoid including areas of low SI (artefacts), several areas (three) will be traced in the visually normal myocardium and the area with the highest SI will be chosen to define normal SI. Epicardium and endocardium will be excluded from these ROIs.

A myocardial area will be regarded as hyperintensive, when the SI is more than 2 standard deviations above the SI in the normal myocardium. The image window will be adjusted to this threshold (normal SI + 2 standard deviations) and the volume of the hyperintensive areas will manually traced and added up. Small areas of hyperintensity scattered throughout the normal myocardium will not be considered a part of AAR.

For each patient AAR will be expressed as a percent of total LV myocardial volume (%) and as absolute mass (g) using a density of 1.05 g/ml. Hypointensive areas within the AAR (haemorrhage or microvascular obstruction) will be considered a part of the AAR. Hyperintensity in the blood pool from slow flowing blood adjacent to the endocardium will be excluded.

The myocardial salvage index can then be calculated as follows:

$$\frac{AAR(g) - Infarct\ Size\ (g)}{AAR\ (g)}$$

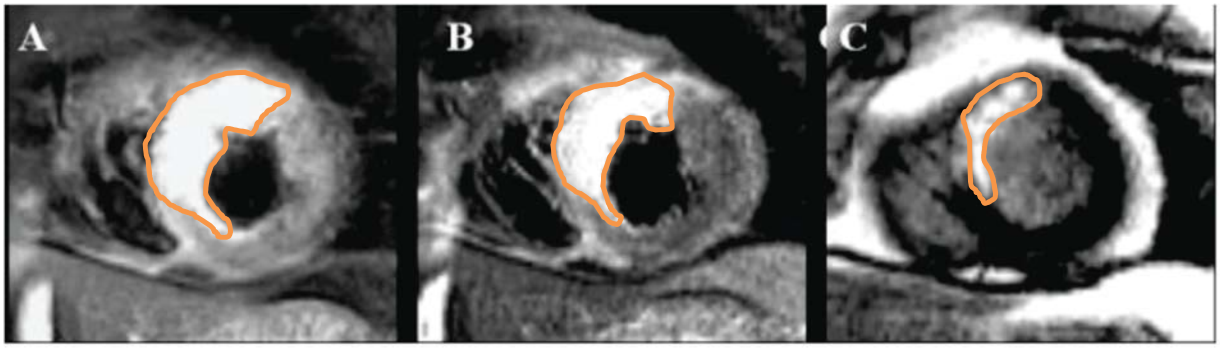

Figure 4: T2-weighted imaging ***(A)*** *T2-weighted CMR image acquired with echo time 65msec, giving an area-at-risk of 31%LV.* ***(B)*** *T2-weighted CMR image with echo time 100ms, giving an area-at-risk of 28%LV.* ***(C)*** *LGE CMR giving an infarct size of 9%LV. (Image from [3] with manual delineation of the areas)*

3.5 Analysis of the Flow imaging (Analysis of stenosis, regurgitations and shunts)

Following the SCMR guidelines for the processing of flow images, the borders of the vessels must be delineated on the magnitude image and reported on the phase image which contains the encoded information (see Figure 5). From this parameters such as the antegrade volume, the retrograde volume, the peak velocity and the mean velocity can be directly calculated.

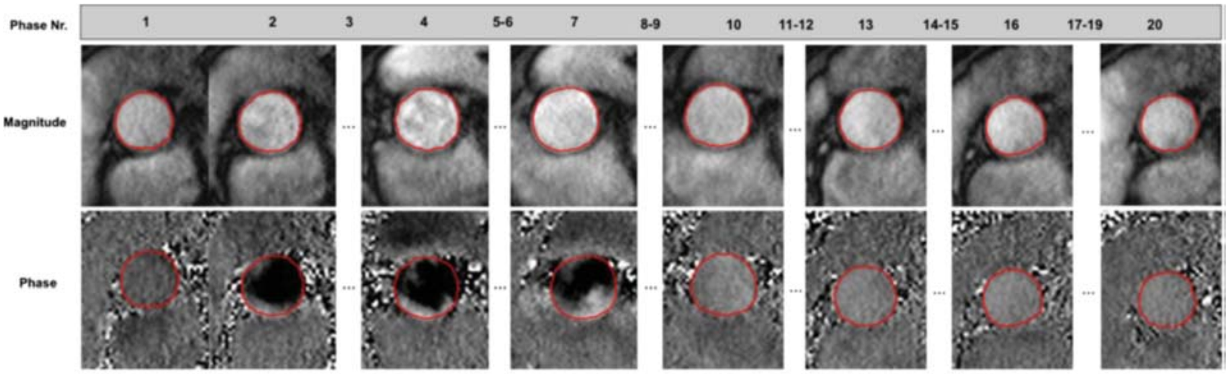

Figure 5:Quantification of blood flow. Contours were drawn delineating the aortic lumen at the sinotubular level during all 20 phases of the cardiac cycle to assess aortic flow. (Image from [1])

We derive from these parameters the cardiac output, the regurgitation ratio and assess the existence of stenosis or shunts.

3.6 Analysis of the T1 MOLLI map (Analysis of myocardial fibrosis)

T1 mapping enables direct myocardial signal quantification (in milliseconds) on a standardized scale. There is no SCMR recommendations for the processing of T1 MOLLI map. This imaging allows a better characterization of myocardial tissue composition on a global or regional level. Myocardial areas of delayed enhancement can be measured in terms of their spatial extent, but also in terms of the magnitude of their signal intensity: The composition of each myocardial slice can be analysed as a T1 distribution histogram, which gives a more accurate description of the myocardial tissue composition (Figure 6). We could use this T1 distribution (mean T1 peak value, distribution scatter) to identify specific myocardial patterns such as myocardial diffuse fibrosis.

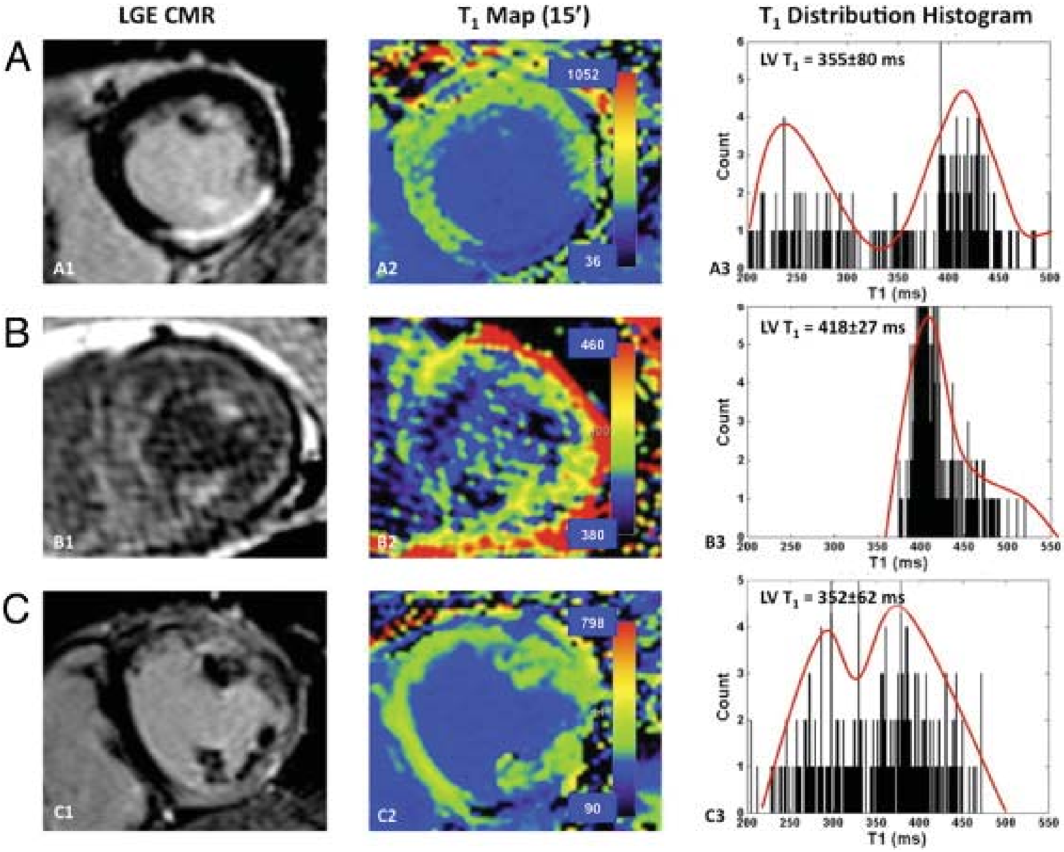

Figure 6: T1-weighted imaging. Comparison of LGE Studies with Corresponding T1 Maps and T1 Values Distribution Histograms in Different Cardiomyopathies : (A) Chronic inferior myocardial infarction; (B) cardiac amyloidosis; (C) nonischemic dilated cardiomyopathy. In each example, the short-axis late gadolinium images show images with different patterns of enhancement, transmural localized in the case of a myocardial infarction scar (A1), sub-endocardial diffuse in the case of cardiac amyloidosis (B1), or sub-epicardial and heterogeneous in the case of dilated cardiomyopathy (C1). In the middle panel are the

corresponding T1 maps (A2, B2, C2) obtained after MOLLI acquisitions. From those T1 maps, a mean left-ventricular (LV) T1 value can be obtained. This information can also be processed more precisely through the analysis of the distribution histogram of the LV T1 values. (Image from [4])

3.7 Analysis of the Tagged MRI sequence (Analysis of myocardial stunning)

Registering the Tagged MRI sequence allows to compute the strain of each myocardial region in order to detect zones with abnormal motion. There is no SCMR recommendations for the processing of Tagged MRI, we will therefore use in-house software to automatically track the left ventricle and compute the strain values related to the local contractile function. We can then determine stunned myocardium which is the result of an ischemic insult leading to contractile dysfunction despite adequate reperfusion.

3.8 IMAGE PROCESSING

An overview of the processing pipeline that will be applied to the data is provided in the figure below:

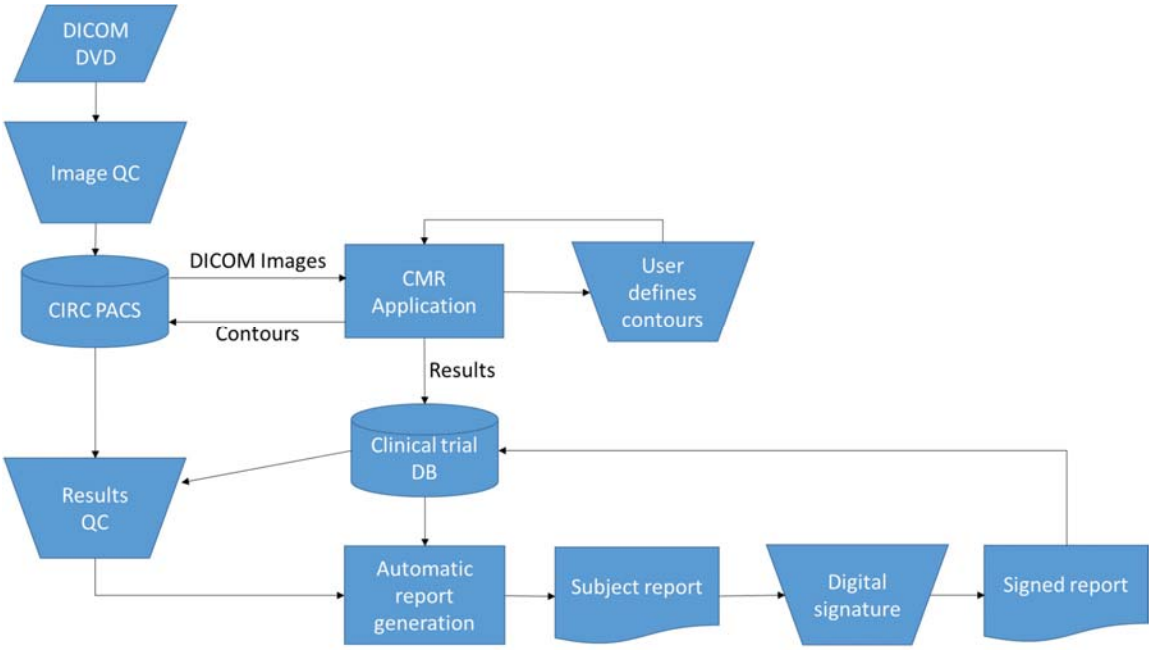

Figure 7.Overview of the image processing steps involved from receipt of subject DVD through to subject analysis report.

3.9 Data Uploading

When the DVD is received by CIRC, the DICOM imaging data is put through the quality control (QC) procedure as described in section 5.1. Data that successfully passes the *Image*

QC is then loaded onto the CIRC PACS. The imaging data stored on PACS is source for all subsequent image processing, the subject DVD is achieved, but no longer used as part of the pipeline.

Note that while the imaging data will be stored at CIRC for the duration of the trial, each scanning site is responsible for storage and archiving of the subject data.

4.0 Image Analysis Overview

The imaging data will be analysed in the following manner:

- The original DICOM data will be pulled from PACS into the CMR application.
- The images will be analysed according to the protocol described in section 3.
- The results are then sent to CIRC's clinical trial database.
- The contours defined during analysis and will be sent back to PACS for QC purposes.
- The results, along with the contours and original DICOM data are put through QC.
- Upon successful completion of the *results QC* process, a report is automatically generated from the clinical trial database.
- The analysis report is digitally signed by a member of the image analysis team. This digital signature is applied to the report in order to conform to FDA Code of Federal Regulations Title 21 Part 11 (CFR 21 Part 11) [6].
- The report and signature are inserted into the clinical trial database, to be held along with the generated results.
- The clinical trial database is capable of producing audit trials that conform to CFR 21 Part 11.

5.0 Machine Learning Pipeline

The deep learning pipeline consists of 4 automatic steps as illustrated in Fig. 1 (adapted from Hakim Fadil JJT et al: A Deep Learning Pipeline for Automatic Analysis of Multi-Scan Cardiac MRI. *Journal of Cardiovascular Magnetic Resonance.* 2020 accepted for publication)

1)Pre-processing For each sequence (Cine, LGE, native T1, post-contrast T1, native T2, AO Flow), the 2D images are resized to 212 x 212 pixels of 1.37mm x 1.37mm resolution, with normalized intensity to deal with the possible variability in sizes, resolution and intensity.

2)Deep Learning Segmentation Each 2D image is then propagated through a sequence specific U-Net 2D model [16] that has been trained on the respective data. All models are trained to predict the anatomical structures of interest present on their sequence images. The cine, LGE, native T1, post-contrast T1, and native T2 models are trained to segment the left ventricular endocardium, and myocardium. Additionally, the Cine model is also trained to segment the right ventricular endocardium and the LGE model to predict the scar tissue in the myocardium. Similarly, the AO Flow model predicts the aorta contour. All U-Net 2D models were trained using the Adam optimizer (learning rate of 0.01, $\beta_1 = 0.9$, $\beta_2 = 0.999$, batch size = 5) to maximize the foreground Dice with the exception of the cine model that minimize a weighted cross entropy loss [18]. The training was performed using a GPU NVIDIA K40 for approximately 24 hours.

3)Post-processing Finally, the U-Net 2D model generates a softmax prediction containing the probabilities of each pixel to belong to a certain region (endocardium, myocardium, ...). The region with the highest probability is selected for every pixel. The 2D predictions are rescaled to the original size and resolutions, and stacked to obtain a 3D mask. The largest connected component for each region is kept to remove isolated pixels. To guarantee the convexity of the endocardium, myocardium and aorta contours, the convex hulls of their pixels are defined and chosen as the final segmentations. Moreover, in the case of the LGE, native T1, post-contrast T1, and T2, the U-Net 2D models tend to over-segment around the basal and apical slices, as shown in Fig. 2. To cope with this issue, Random Forests (RF) classifiers have been trained to identify these segmentations and discard them, improving the overall 3D segmentation. We employed Random Forests with T=6 trees and a maximum depth of D=6, while the features used were the mean softmax of the predicted myocardium, its mean intensity and the normalized slice position within the 3D stack. The full automatic segmentation pipeline takes less than 0.2 seconds for a 2D image, 50 seconds for a full CINE stack (~250 images), 2 seconds for a stack of structural images (~ 10 images), and 5 seconds for an AO sequence (~25 images) on a GPU (Nvidia GTX 1050).

4)Parameters extraction From the segmentation of the anatomical structures, several main functional parameters are extracted depending on the analyzed sequence and clinical need. For example among others, the left and right ejection fraction and stroke volumes are extracted from Cine

images; the scar percentage within the myocardium is measured from LGE; from the native T1, post-contrast T1 and T2 maps, the mean relaxation time within the myocardium is obtained; finally, the net and backward flow amplitudes are retrieved from the segmentation of the AO Flow phase-contrast image.

5)Full study automatic reporting Finally, the parameters extracted for each patient in the study are reported in a large statistical file where outliers are automatically extracted using expected physiological ranges. These outliers are further processed by clinicians to evaluate the quality of the automatic segmentation and to correct them if necessary. Using a subset of the dataset, mean errors can also be measured by comparing the automatic to the corrected segmentation, as an indicator of the global confidence in the automatic measurements.

Figure 1 Fully automatic multi-scan CMR analysis pipeline.

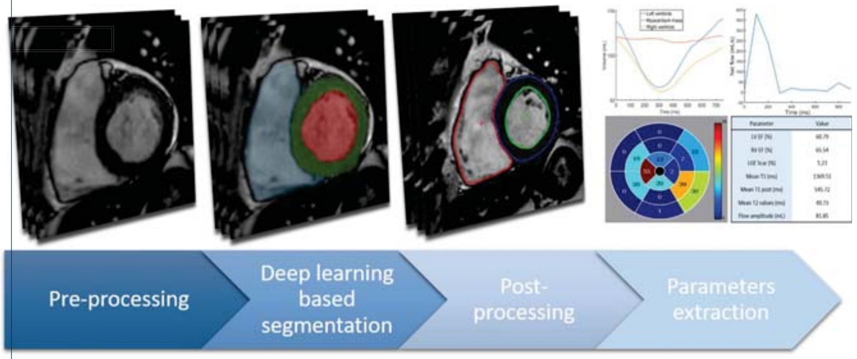

Figure 2 An example of over-segmentation around the basal slice (a). (b) The blood pool and the muscle is segmented by the U-Net 2D model as the endocardium (red) and myocardium (green).
