# Supplementary material for: Remote Postdischarge Treatment of Patients With Acute Myocardial Infarction by Allied Health Care Practitioners vs Standard Care: The IMMACULATE Randomized Clinical Trial
Source: JAMA Cardiol. 2020 Dec 30;6(7):1–6. doi: 10.1001/jamacardio.2020.6721 (PMC7774042; doi:10.1001/jamacardio.2020.6721)
Supplement: Supplement 4. — Data sharing statement [file jamacardiol-e206721-s004.pdf]

Data Sharing Statement

Chan. Remote Postdischarge Treatment of Patients With Acute Myocardial Infarction by Allied Health Care Practitioners vs Standard Care. *JAMA Cardiol*. Published December 30, 2020. doi:10.1001/jamacardio.2020.6721

Data
Data available: Yes
Data types: Deidentified participant data
How to access data: Deidentified data will be made available upon reasonable request. All data requests to be sent to mark.chan@nus.edu.sgm.
When available: With publication

Supporting Documents
Document types: Informed consent form, Statistical/analytic code
How to access documents: All requests to be sent to mark.chan@nus.edu.sgm.
When available: With publication

Additional Information
Who can access the data: Researchers whose proposed use of the data has been approved
Types of analyses: For metanalyses.
Mechanisms of data availability: Signed data access agreement
Any additional restrictions: None.
